# Functional and molecular characterisation of EO771.LMB tumours, a new C57BL/6-mouse-derived model of spontaneously metastatic mammary cancer

**DOI:** 10.1242/dmm.017830

**Published:** 2015-01-29

**Authors:** Cameron N. Johnstone, Yvonne E. Smith, Yuan Cao, Allan D. Burrows, Ryan S. N. Cross, Xiawei Ling, Richard P. Redvers, Judy P. Doherty, Bedrich L. Eckhardt, Anthony L. Natoli, Christina M. Restall, Erin Lucas, Helen B. Pearson, Siddhartha Deb, Kara L. Britt, Alexandra Rizzitelli, Jason Li, Judith H. Harmey, Normand Pouliot, Robin L. Anderson

**Affiliations:** 1 Research Division, Peter MacCallum Cancer Centre, St Andrew’s Place, East Melbourne, VIC 3002, Australia; 2 Sir Peter MacCallum Department of Oncology, University of Melbourne, Parkville, VIC 3010, Australia; 3 Department of Pathology, University of Melbourne, Parkville, VIC 3010, Australia; 4 Department of Pharmacology & Therapeutics, University of Melbourne, Parkville, VIC 3010, Australia; 5 Angiogenesis and Metastasis Research, Royal College of Surgeons in Ireland, 123 St Stephen’s Green, Dublin 2, Ireland; 6 Morgan Welch Inflammatory Breast Cancer Research and Clinic, Department of Breast Medical Oncology, The University of Texas, MD Anderson Cancer Center, Houston, TX 77030, USA; 7 Department of Anatomical Pathology, Royal Melbourne Hospital, Parkville, VIC 2010, Australia

**Keywords:** Breast cancer, Syngeneic preclinical models, Metastasis, Tumour subtyping, Estrogen receptor alpha

## Abstract

The translation of basic research into improved therapies for breast cancer patients requires relevant preclinical models that incorporate spontaneous metastasis. We have completed a functional and molecular characterisation of a new isogenic C57BL/6 mouse model of breast cancer metastasis, comparing and contrasting it with the established BALB/c 4T1 model. Metastatic EO771.LMB tumours were derived from poorly metastatic parental EO771 mammary tumours. Functional differences were evaluated using both *in vitro* assays and spontaneous metastasis assays in mice. Results were compared to non-metastatic 67NR and metastatic 4T1.2 tumours of the 4T1 model. Protein and transcript levels of markers of human breast cancer molecular subtypes were measured in the four tumour lines, as well as *p53* (*Tp53*) tumour-suppressor gene status and responses to tamoxifen *in vivo* and *in vitro*. Array-based expression profiling of whole tumours identified genes and pathways that were deregulated in metastatic tumours. EO771.LMB cells metastasised spontaneously to lung in C57BL/6 mice and displayed increased invasive capacity compared with parental EO771. By immunohistochemical assessment, EO771 and EO771.LMB were basal-like, as was the 4T1.2 tumour, whereas 67NR had a luminal phenotype. Primary tumours from all lines were negative for progesterone receptor, Erb-b2/Neu and cytokeratin 5/6, but positive for epidermal growth factor receptor (EGFR). Only 67NR displayed nuclear estrogen receptor alpha (ERα) positivity. EO771 and EO771.LMB expressed mutant p53, whereas 67NR and 4T1.2 were *p53*-null. Integrated molecular analysis of both the EO771/EO771.LMB and 67NR/4T1.2 pairs indicated that upregulation of matrix metalloproteinase-3 (MMP-3), parathyroid hormone-like hormone (Pthlh) and S100 calcium binding protein A8 (S100a8) and downregulation of the thrombospondin receptor (Cd36) might be causally involved in metastatic dissemination of breast cancer.

## INTRODUCTION

The high mortality rate associated with advanced breast cancer is due primarily to the growth of metastases, mainly targeting liver, lung, bone and brain. Treating metastatic disease poses many challenges owing to a lack of knowledge of the critical molecular targets for therapy and to the acquisition of resistance both to standard chemotherapy and targeted therapies such as selective estrogen receptor modulators (SERMs) and the anti-HER2 (human epidermal growth factor receptor 2; also known as Erb-b2) antibody, trastuzumab (Eckhardt et al., 2012). The genetic diversity between different tumours and the inherent heterogeneity of individual breast lesions poses additional challenges (Russnes et al., 2011; Stephens et al., 2012).

Gene expression profiling of breast tumours has led to the identification of up to ten subtypes in humans (Curtis et al., 2012; The Cancer Genome Atlas Network, 2012; Lehmann et al., 2011; Perou et al., 2000; Sørlie et al., 2001) and many subtypes in transgenic mouse models of breast cancer (Herschkowitz et al., 2007; Pfefferle et al., 2013). These include the well-accepted luminal A, luminal B, basal-like and HER2-enriched subtypes (Herschkowitz et al., 2007; Perou et al., 2000; Sørlie et al., 2001).

Immunohistochemical phenotyping of tumours has identified the minimum set of biomarkers required to distinguish these groups; in particular, basal-like and triple-negative (ER-, PR- and HER2-negative) tumours (Blows et al., 2010; Cheang et al., 2008; Nielsen et al., 2004). However, more relevant prognostic biomarkers and therapeutic targets are now required within each of the molecular subtypes of human breast cancer, but especially for basal-like and triple-negative tumours, for which no targeted therapies currently exist.

Mouse models of breast cancer include those in which xenografts, either from human cell lines or directly from patients, are transplanted into immunocompromised hosts (DeRose et al., 2011). However, it is becoming increasingly evident that faithful tumour-stromal interactions are very important for clinically relevant tumour progression and metastasis. Although xenografts are of value for the assessment of intrinsic events within the tumour cells, they lack the orthologous extracellular matrix (ECM), the species-matched stromal-tumour interactions and a functional immune system. Syngeneic mouse models of breast cancer, either transgenic or transplantable, overcome these limitations. Transgenic mouse mammary tumour models are of great value to preclinical studies because they incorporate the initiation of the primary tumour. However, with some exceptions, including the murine mammary tumour virus (MMTV)-polyoma middle T antigen (PyMT) (Guy et al., 1992a) and the MMTVneuNT transgenic mice (Guy et al., 1992b; Moody et al., 2002; Muller et al., 1988), the timeframe for tumour development is often months, and metastasis is generally limited to a modest number of lung nodules (Varticovski et al., 2007).

**TRANSLATIONAL IMPACT**

**Clinical issue**

Metastatic disease is the most common cause of major morbidity and death in cancer patients. Approximately 20% of individuals with breast cancer ultimately die from the disease, nearly all owing to the onset of incurable metastatic disease. Hence, research efforts should focus on the identification of metastasis-regulating genes and on the development of new therapies that could prevent the expansion of secondary metastatic lesions. An essential component of development and testing of new pharmacological agents is the assessment of their efficacy in preclinical settings prior to clinical trials. However, very few preclinical models that incorporate the relevant features of human metastatic disease are available. To be relevant to human metastatic breast cancer, these preclinical models should incorporate tumours that are derived from syngeneic (genetically identical) animals – so that they can retain an intact immune system – and tumours should be implanted orthotopically (in the area in which the cancer typically arises). Because breast cancer comprises different tumour subtypes, each requiring different clinical management, it is necessary to develop a range of preclinical models that can reflect this heterogeneity.

**Results**

In this work, the authors describe a new mouse model of metastatic breast cancer based on a spontaneous mammary tumour that arose in a C57BL/6 mouse. From this line, designated EO771, they have derived a tumour variant that is metastatic to the lung (called EO771.LMB). The functional characteristics of EO771 and EO771.LMB tumour lines were compared to those of a pre-existing and widely used mouse model of metastatic mammary cancer, using immunohistochemistry and molecular analyses. By immunohistochemistry, EO771 and EO771.LMB lines were classified as a basal-like tumour, a subtype with poor prognosis in humans. Integrated molecular analysis of these tumours revealed important genes – including those that encode matrix metalloproteinase-3 (MMP-3) and parathyroid hormone-like hormone (Pthlh) – whose dysregulation might be causally involved in metastatic dissemination of breast cancer.

**Implications and future directions**

This new EO771 metastasis model will provide a valuable option for assessing genes that regulate metastasis and for developing new therapies that might be beneficial for individuals with poor-outcome basal-like tumours, who have a higher probability of developing metastatic disease. A further advantage of this model is that it grows in the C57BL/6 mouse strain, which is commonly used to generate transgenic and knockout mice. Hence, tumours derived from this new model will be valuable for analysing the contribution of specific host-derived genes to the metastatic process.

For transplantable models, the tumour cells can be inoculated into the mammary gland as an allograft with genetic and immunological compatibility and the resulting tumours often progress extensively beyond localised growth within a timeframe of weeks rather than months (Varticovski et al., 2007). Genetic manipulation of the cells prior to tumour establishment is also easily achievable. Thus, for studies relating to spontaneous metastasis, transplantable models offer a valuable and cost-efficient alternative. These models are generally established from tumour cells isolated from spontaneous mammary tumours in mice (Aslakson and Miller, 1992; Casey et al., 1951; Lelekakis et al., 1999; Rockwell and Kallman, 1973). The BALB/c isogenic series of mammary tumour lines collectively known as ‘the 4T1 model’ has been the principal transplantable mouse model used to study both tumour- and host-derived factors involved in spontaneous metastasis (Aslakson and Miller, 1992; Eckhardt et al., 2005; Kusuma et al., 2012).

Studies of host-derived factors in oncogenesis are facilitated by inoculating cells into mice that are deficient for the gene under study. Because the majority of genetically ‘pure’ knockout mice have been generated on, or backcrossed onto, the C57BL/6 background, it is desirable to have metastatic transplantation models in this strain. However, C57BL/6 mice are more resistant than BALB/c mice to mammary tumorigenesis induced by p53 loss (Kuperwasser et al., 2000) and are more resistant to metastasis (Hunter, 2012; Lifsted et al., 1998). Moreover, the role of specific host factors in tumour progression can vary depending on the mouse strain used (Martin et al., 2008).

We have now developed and characterised a new syngeneic mouse model of metastatic breast cancer in C57BL/6 mice. EO771.LMB cells were isolated from a rare metastatic lung nodule that had disseminated from an orthotopic primary tumour of the original EO771 mammary adenocarcinoma cell line, derived from a spontaneous mammary tumour in a female C57BL/6 mouse (Casey et al., 1951). Herein, we have characterised the *in vitro* and *in vivo* phenotypes of the EO771 and EO771.LMB lines. In addition, immunohistochemical profiles of EO771 and EO771.LMB tumours were obtained and compared to those of non-metastatic (67NR) and highly metastatic (4T1.2) tumours from the 4T1 BALB/c model. Array-based gene expression profiling was conducted to identify genes commonly dysregulated in metastatic tumours from both isogenic pairs.

Based on immunohistochemical analyses using a five-marker panel and array-based gene expression profiling, we show that these four tumours display features of both luminal and basal-like subtypes. Expression profiling of the isogenic non-metastatic/metastatic pairs identified the genes encoding matrix metalloproteinase-3 (MMP-3), S100 calcium binding protein A8 (S100a8), S100a9 and parathyroid hormone-like hormone (Pthlh) as possible drivers of metastatic progression.

## RESULTS

### *In vitro* and *in vivo* characterisation of EO771.LMB: a new syngeneic model of metastatic breast cancer

EO771.LMB was isolated from a rare spontaneous lung metastasis from an EO771 tumour-bearing mouse as described in the Materials and Methods. Both EO771 and EO771.LMB formed undifferentiated high-grade primary tumours in C57BL/6 mice *in vivo* (supplementary material Fig. S1), as did 67NR and 4T1.2 from the established 4T1 BALB/c model. Following summation of scores for the number of tubules present, nuclear pleomorphism and the number of mitoses, each of the four tumour types were considered high-grade overall. Interestingly, 67NR tumours produced moderate-grade nuclei, rather than the high-grade nuclei observed in the other three tumour types (supplementary material Fig. S1).

**Fig. 1.**
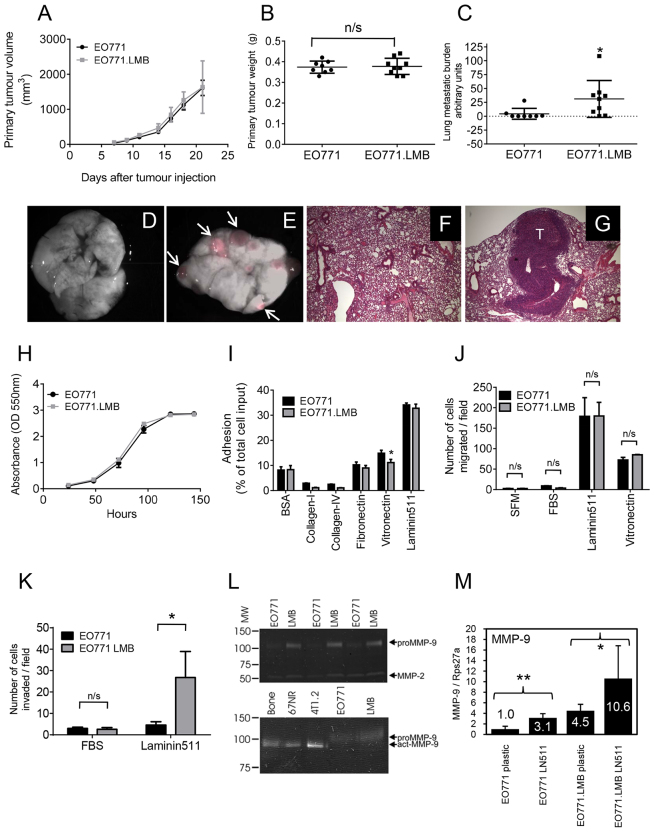
**Functional characterisation of the parental EO771 tumour line and its metastatic variant, EO771.LMB.** (A) Parental EO771 (*n*=10) and metastatic variant EO771.LMB (*n*=5) primary tumour volumes were measured three times per week following implantation of cells into the mammary gland of C57BL/6 mice [mean tumour volume (mm^3^) ± s.d.]. (B) Primary tumour weights following resection on day 13 after implantation (mean weight ± s.d.; *n*=8, EO771; *n*=9, EO771.LMB). (C) Tumour burden in the lungs (mean ± s.d.) measured by qPCR quantification of mCherry DNA in genomic DNA isolated from whole lungs 2 weeks following primary tumour resection (*n*=8, EO771; *n*=9, EO771.LMB). (D,E) Superimposed brightfield/fluorescent image of lungs from EO771 (D) or EO771.LMB (E) tumour-bearing mice. Arrows indicate tumour nodules. (F,G) Hematoxylin- and eosin-stained lung sections from an EO771- (F) or EO771.LMB- (G) bearing mouse. Magnification: 40×. T: metastatic tumour deposit. (H) Proliferation of EO771 and EO771.LMB cells (mean ± s.d. of 12 replicate wells of one of three representative experiments). (I) Adhesion of EO771 and EO771.LMB cells to different substrates after 30 min. Adhesion is represented as the percentage of total cell input [mean of triplicate wells ± s.d. of a representative experiment (*n*=3) is shown]. (J) Chemotactic migration towards serum-free medium (SFM) or 5% (v/v) FBS after 5 hours and haptotactic migration towards laminin-511 or vitronectin after 4 hours, using Transwell inserts. The number of migrated cells was counted from three fields of view per membrane at 20× magnification (mean ± s.d. of one of three independent experiments). (K) Chemotactic and haptotactic invasion through Matrigel towards 5% (v/v) FBS or laminin-511 using Transwell inserts. The assay was run for 18 hours in triplicate wells and the number of invaded cells counted from three fields of view per membrane at 20× magnification. The data represent the mean number of invaded cells ± s.d. of one of two independent experiments. (L) Gelatin zymography of triplicate EO771 and EO771.LMB (LMB) conditioned medium from cells cultured on plastic (upper panel). The positions of molecular weight (MW) markers are shown on the left. The locations of proMMP-9 and MMP-2 are indicated on the right. Mean proMMP-9 band intensity was sixfold higher for EO771.LMB compared with EO771 (*P*=0.0003). Conditioned medium from primary culture of whole bone explant (bone), 67NR and 4T1.2 cells were used as positive controls for active MMP-9 (act-MMP-9) to distinguish from proMMP-9 produced by EO771 lines (lower panel). (M) qRT-PCR analysis of *MMP-9* mRNA levels in mouse mammary tumour cell lines cultured on plastic or laminin-511 (LN-511, 2 μg/ml). Triplicate cultures were set up for each condition and triplicate PCR reactions run for each culture. Thus, each data point represents the mean ± s.d. from nine PCR reactions. Mean expression in EO771 on plastic was set to one. Statistical significance in B,C,I,J,K and M was determined using the Student’s *t*-test, whereas that in A and H were determined using two-way ANOVA. *P<0.05; **P<0.01; n/s, not significant.

Analysis of EO771 and EO771.LMB mammary tumours revealed similar primary tumour growth rates (Fig. 1A) and, when EO771 and EO771.LMB primary tumours were resected 13 days following implantation, comparable primary tumour weights were recorded (Fig. 1B). Lung metastatic burden was assessed 2 weeks after primary tumour resection using various methods. By quantitative PCR of a reporter gene present only in the tumour cells, EO771.LMB tumour-bearing mice were shown to have increased spontaneous lung metastasis (Fig. 1C). Visible surface nodules were increased in EO771.LMB tumour-bearing mice, but this difference did not reach statistical significance (supplementary material Fig. S2). Fluorescence imaging of the mCherry-positive nodules (Fig. 1D,E) and histological analysis (Fig. 1F,G) revealed the presence of large metastatic nodules in mice with EO771.LMB tumours. Metastasis to bone was not detected (data not shown). It is important to note that counting only visible nodules will miss the inclusion of micrometastases or those within the lung parenchyma. However, the trend towards more visible, and hence larger, nodules on the lungs of EO771LMB-bearing mice might be due to earlier release of tumour cells from the primary tumour, resulting in earlier homing of tumour cells to lung and larger metastatic nodules. No difference in lung colonising ability was found between EO771 and EO771.LMB cells in experimental metastasis assays following intravenous inoculation of cells, with both lines giving rise to extensive lung metastasis (supplementary material Fig. S3), indicating key differences between the two lines in the early steps of metastasis.

Using a suite of *in vitro* surrogate assays for metastasis, EO771.LMB cells were compared to parental EO771 cells. There was no morphological distinction between EO771 and EO771.LMB cell lines cultured *in vitro* (supplementary material Fig. S4), with both lines displaying an undifferentiated spindle-shaped morphology. However, the EO771 and EO771.LMB cells were larger and had a more elongated shape compared with the 4T1.2 cells. As found *in vivo*, EO771 and EO771.LMB cells proliferated at the same rate when cultured *in vitro* (Fig. 1H). Thus, enhanced proliferation does not account for the increased metastatic capacity of EO771.LMB tumours. Similarly, no significant differences were found between the two lines in their ability to grow independent of anchorage in soft agar (supplementary material Fig. S5), nor in their capacity to form mammospheres (supplementary material Fig. S6). The number and size of the mammospheres formed by day 10 as primary cultures or by day 7 as secondary mammosphere cultures was similar between the two lines (supplementary material Fig. S6). Thus, we cannot conclude that there are any differences in potential numbers of metastasis-initiating cells between the two lines.

Interaction with the surrounding stroma is an important factor in metastatic dissemination (Bhowmick et al., 2004; Pouliot et al., 2013; Zetter, 1993). Hence, the ability to adhere to various ECM proteins that are present in the microenvironment was measured (Prince et al., 2002). Both lines showed strongest adhesion to laminin-511, whereas EO771.LMB cells had a slightly reduced ability to adhere to vitronectin (Fig. 1I). Neither EO771 nor EO771.LMB were motile towards fetal bovine serum (FBS) in chemotactic migration assays but demonstrated robust motility in haptotactic migration assays towards laminin-511 and to a lesser extent towards vitronectin (Fig. 1J). However, there were no differences between the two lines on either substrate. Although both lines were poorly invasive toward FBS (Fig. 1K), EO771.LMB showed markedly increased haptotactic invasion through Matrigel toward laminin-511 compared with EO771 (Fig. 1K). As a comparison, metastatic 4T1.2 cells were slightly more adherent than 67NR cells on most substrates, with both lines remaining only weakly adherent to collagen I, collagen IV and fibronectin. However, 4T1.2 adhesion to vitronectin and laminin-511 was elevated approximately fourfold compared with 67NR (supplementary material Fig. S7), consistent with previous results using Matrigel as the substrate (Eckhardt et al., 2005). We have shown previously that 4T1-derived metastatic variants display higher chemotactic migration and invasion than 67NR cells (Eckhardt et al., 2005), and are particularly migratory and invasive towards laminin-511 (Chia et al., 2007; Kusuma et al., 2012). These responses are dependent in part on gelatinase activity (Denoyer et al., 2014; Sloan et al., 2006). Gelatin zymography assays revealed the presence of both MMP-2 and MMP-9 in the culture supernatants of EO771 and EO771.LMB (Fig. 1L, upper panel), with EO771.LMB showing sixfold higher MMP-9 protein levels compared with EO771 cells. Only pro-MMP-9 was detected in EO771 or EO771LMB cells *in vitro* (Fig. 1L, lower panel). The absence of detectable active MMP-9 in EO771 and EO771LMB monocultures is likely to be due to the relatively low abundance of MMP-9 in these cultures compared to the levels in whole bone cultures (a positive control for active MMP-9) and/or to the need for a higher concentration of MMP-3 or other proteases present *in vivo* to fully process MMP-9. As reported previously (Tester et al., 2000), 4T1.2 cells produce more active MMP-9 than do 67NR cells (Fig. 1L, lower panel). Because laminin-511 selectively induced the invasive capacity of EO771.LMB (Fig. 1K), we evaluated whether engagement with this substrate modulated MMP-9 expression. Basal *MMP-9* mRNA levels in cells cultured on plastic were 4.5-fold higher in EO771.LMB compared with EO771 (Fig. 1M), consistent with the higher protein levels. Culturing of cells on laminin-511 significantly increased *MMP-9* mRNA levels by 3.1-fold in EO771 and by 2.4-fold in EO771.LMB relative to the cells seeded on plastic.

### Immunohistochemical profiling of mouse models of breast cancer

Standard clinical phenotyping of human tumours involves immunohistochemical staining for estrogen receptor alpha (ERα), progesterone receptor (PR) and HER2 amplification or high expression, to inform selection of therapy. Additional staining for cytokeratin 5/6 (KRT5/6), epidermal growth factor receptor (EGFR) and Ki-67 has also been used as a surrogate for gene expression profiling to allocate tumours to luminal A, luminal B, HER2 or basal-like molecular subtypes (Blows et al., 2010). Inclusion of the three additional markers better predicts survival (Cheang et al., 2008) and loco-regional relapse (Voduc et al., 2010).

**Fig. 2.**
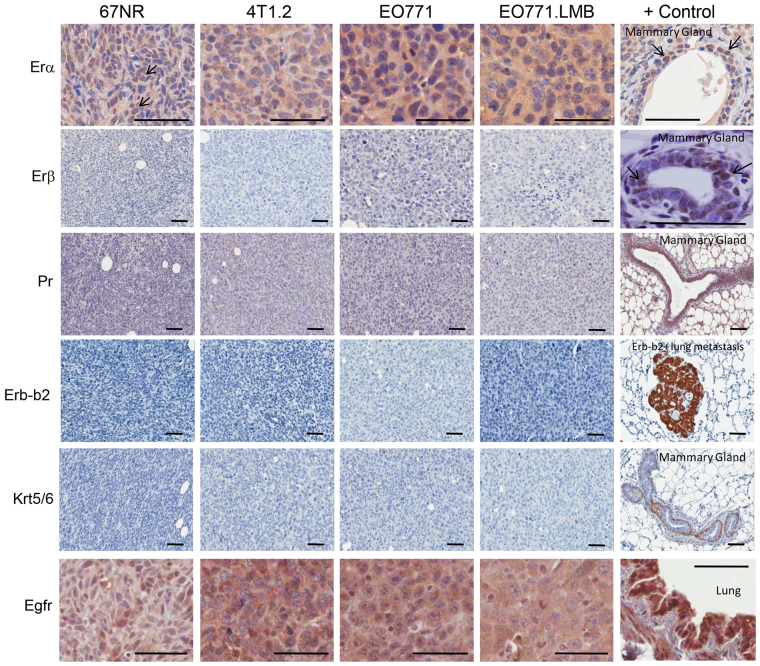
**Immunohistochemical staining of primary murine mammary tumours for markers of human breast cancer subtypes.** From top to bottom: Luminal marker ERα. A normal mammary duct adjacent to a 67NR tumour was used as a positive control. Arrows indicate examples of ERα-positive nuclei in 67NR tumour and mammary duct. Luminal marker ERβ. A normal mammary duct was used as a positive control with arrows indicating examples of ERβ-positive nuclei. Luminal marker PR. A normal mammary gland shows high levels of this protein. Erb-b2/Neu. A metastatic tumour from the lungs of an MMTV-Neu transgenic mouse was used as a positive control. Basal markers cytokeratin 5/6 (Krt5/6) and EGFR. Positive controls (myoepithelial layer of mammary gland for Krt5/6 and mouse lung for EGFR) are shown at the right of each row. Scale bars: 50 μm.

Thus, primary EO771 and EO771.LMB tumours were analysed by immunohistochemistry (Fig. 2) for markers of the luminal A/B subtypes (ERα, ERβ, PR), the HER2 subtype (Erb-b2/Neu) and the basal-like subtype (KRT5/6, EGFR) and compared to 67NR and 4T1.2 tumours. ERα is located in the nuclei of primary human breast cancers (Pertschuk et al., 1985) and ERα-positive MCF7 xenografts, but is absent in the triple-negative MDA-MB-231 tumours (supplementary material Fig. S8). 67NR tumours revealed nuclear ERα positivity as well as diffuse cytoplasmic staining. Nuclear staining was negligible in the other three tumours, which instead showed diffuse cytoplasmic positivity (Fig. 2). Cytoplasmic ERα is also present at low incidence in human breast cancer specimens (Welsh et al., 2012), but the significance of this localisation is not known. The closely related ERβ protein, which is required for normal terminal differentiation of the murine mammary gland (Förster et al., 2002), was not present in any of the tumours (Fig. 2). Similarly, all four tumours were negative for PR and Erb-b2/Neu protein. In humans, tumours are considered basal-like if they are negative for nuclear ERα, PR and HER2 (triple-negative) but positive for KRT5/6 or EGFR (Carey et al., 2006; Cheang et al., 2008; Nielsen et al., 2004). All four tumours were negative for KRT5/6 protein but positive for EGFR staining (Fig. 2). Therefore, immunohistochemical analysis indicates that 4T1.2, EO771 and EO771.LMB have a triple-negative and basal-like phenotype, whereas 67NR, owing to the presence of nuclear ERα, displays a mixed luminal/basal phenotype.

### *p53* status of mouse mammary tumours

**Fig. 3.**
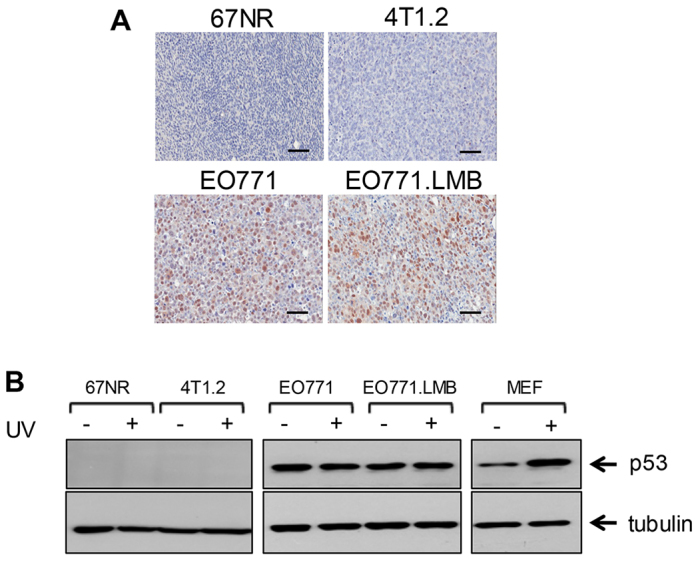
**Evaluation of p53 status.** (A) Immunostaining of primary tumours for tumour suppressor p53. Scale bars: 50μm. (B) Cell lines were exposed to UVC irradiation (+) or sham-irradiated (−) and then assessed 4 hours later for p53 levels by western blot. The blots were re-probed with an antibody for α-tubulin as a loading control. UVC-treated mouse embryonic fibroblasts (MEF) with wild-type p53 were used as a positive control. The data are representative of two independent experiments.

Mutations in p53 are associated with the basal-like subtype of breast cancer (Carey et al., 2006). Because point mutations in *p53* stabilise the protein, a positive signal by immunohistochemistry is commonly used as a surrogate marker of tumours bearing a missense mutation (Wynford-Thomas, 1992; Yemelyanova et al., 2011). Both EO771 and EO771.LMB primary tumours showed uniform nuclear staining for p53, whereas 67NR and 4T1.2 were negative (Fig. 3A). Constitutive p53 expression by cultured EO771 and EO771.LMB cells was confirmed by western blot, and protein levels could not be further enhanced by exposure to ultraviolet (UV) radiation, indicating the presence of mutant p53. In contrast, p53 is inducible in mouse embryonic fibroblasts that express wild-type p53 (Fig. 3B). Both control and UV-radiation-exposed 67NR and 4T1.2 cells were negative for p53, which is in agreement with earlier reports on the 4T1 model (Wang et al., 1998; Yerlikaya and Erin, 2008).

### Responses of murine mammary tumours to tamoxifen

**Fig. 4.**
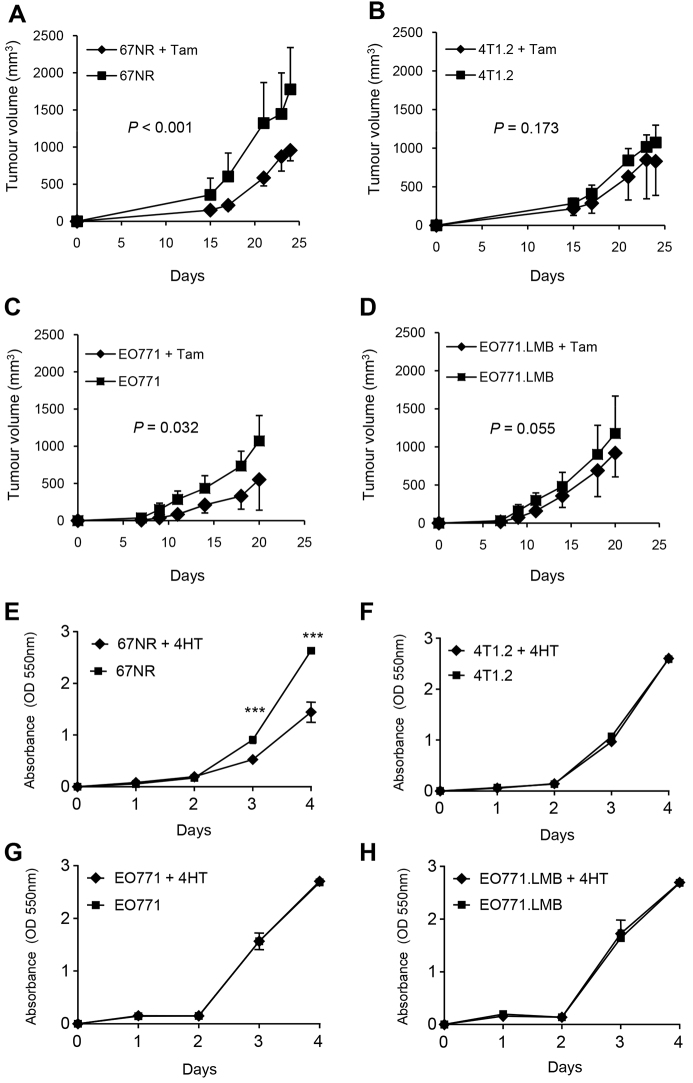
**Response of primary murine mammary tumours and cell lines to treatment with an estrogen receptor antagonist.** Mice were implanted with 67NR (A), 4T1.2 (B), EO771 (C) or EO771.LMB (D) cells and divided into two groups. One group received tamoxifen (Tam, 10 μg/g of chow) beginning on the day of cell implantation and the other did not receive tamoxifen. For 67NR and 4T1.2 tumour-bearing mice, *n*=6/group, for EO771 and EO771.LMB, *n*=10/group. Graphs depict the mean tumour volume (mm^3^) ± s.d. for each group. (E-H) Response of cultured tumour cells to treatment with 4-hydroxytamoxifen (4HT). Cells were incubated with vehicle alone (1% ethanol) or 4HT at 500 nM. Proliferation (mean ± s.d. of six replicate wells) was measured over 4 days. One of two representative experiments is shown. ***P<0.001 by Student’s *t*-test.

Given the lack of nuclear ERα protein but the presence of diffuse cytoplasmic ERα in the EO771-derived tumours and in 4T1.2 tumours, we determined whether their growth was impacted by administration of the ERα antagonist tamoxifen to the mice, with continuous treatment beginning on the day of tumour-cell inoculation. Tamoxifen significantly inhibited growth of 67NR tumours (Fig. 4A), consistent with the presence of nuclear ERα. Tamoxifen administration had no significant effect on the growth of 4T1.2 (Fig. 4B) or EO771.LMB (Fig. 4D), but did reduce growth of EO771 tumours (Fig. 4C). To help gain insight into epithelial versus possible stromal actions of tamoxifen *in vivo*, proliferation *in vitro* was measured in response to the active metabolite 4-hydroxytamoxifen. 4-hydroxytamoxifen significantly abrogated proliferation in 67NR cells (Fig. 4E), but had no effect on the growth rates of 4T1.2, EO771 or EO771.LMB cells (Fig. 4F–H). Thus, response to tamoxifen *in vitro* was correlated with the presence of nuclear ERα; the reduction of EO771 tumour growth and the trend towards decreased growth of EO771.LMB and 4T1.2 tumours *in vivo* upon tamoxifen administration might be attributable to effects on stromal cells.

**Fig. 5.**
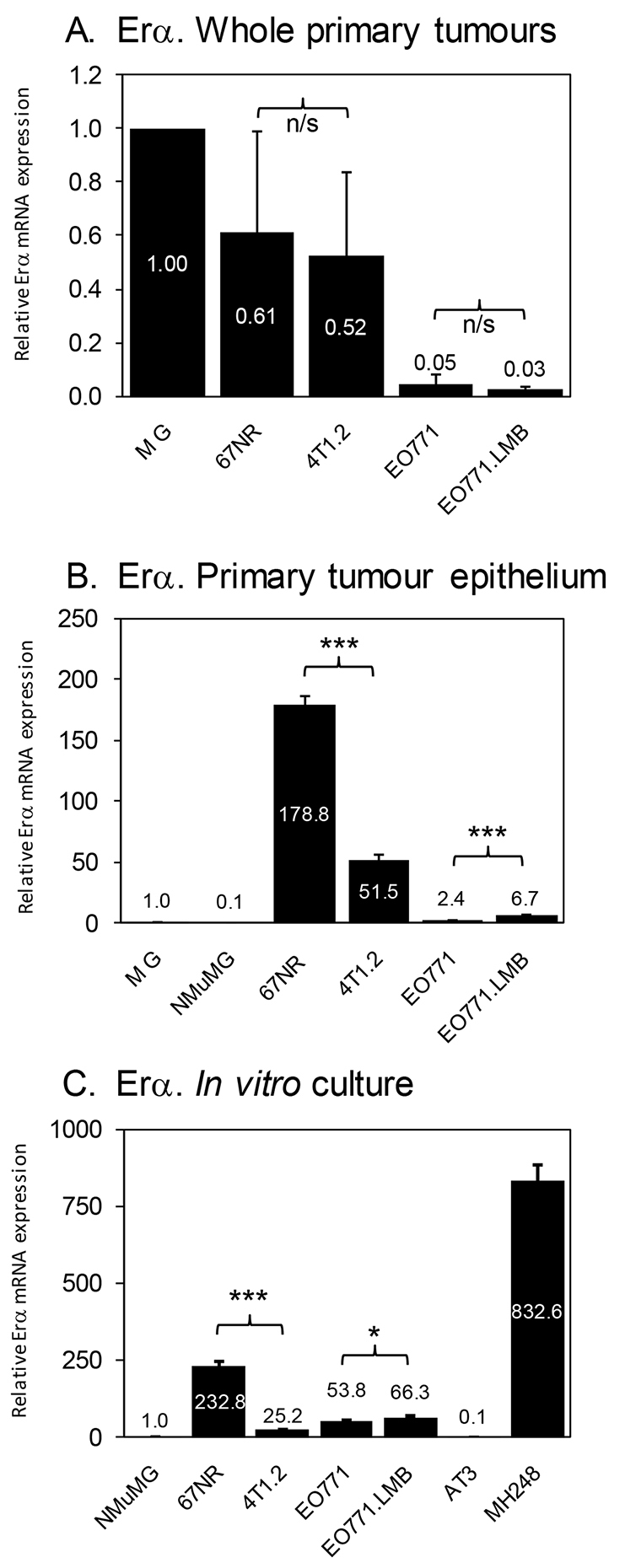
**Analysis of *ERα* mRNA expression.** (A) qRT-PCR analysis of *ERα* mRNA levels in whole primary tumours. Three different primary tumours were analysed in duplicate by qRT-PCR for each tumour model. Thus, each data point represents the mean ± s.d. of six qPCR reactions across three different tumours. Expression in whole mammary gland (MG) was set to 1. (B) qRT-PCR analysis of *ERα* mRNA levels in whole mammary gland (MG), cultured NMuMG cells and in isolated tumour epithelial cells from the indicated primary tumours. RNA was isolated from the primary tumour cells of two mice for each tumour type and pooled. Each data point represents the mean ± s.d. of triplicate reactions. Expression in mammary gland (MG) was set to 1. (C) qRT-PCR analysis of *ERα* mRNA levels in cultured mammary tumour cell lines. Each data point represents the mean ± s.d. of triplicate reactions. Expression in NMuMG cells was set to 1. AT3 and MH248 murine mammary tumour lines were used as negative and positive controls, respectively. *P<0.05; ***P<0.001; n/s, not significant.

The different responses to tamoxifen prompted us to assess *ERα* transcript levels in tumours and cultured cells (Fig. 5). *ERα* mRNA levels in whole 67NR and 4T1.2 tumours were higher than those in EO771 and EO771.LMB tumours, although no significant differences were found between 67NR and 4T1.2, or between EO771 and EO771.LMB pairs (Fig. 5A). However, tumour cells isolated from 67NR primary tumours expressed three- to four-fold higher *ERα* mRNA levels than those from 4T1.2 (Fig. 5B), confirming their intrinsic responsiveness to tamoxifen. Cells isolated from EO771.LMB tumours expressed more *ERα* than did EO771 (Fig. 5B), but the low expression in both lines compared with that in 67NR indicates that the *in vivo* responsiveness of EO771 tumours to tamoxifen is most likely mediated by the stromal compartment. The differential expression of ERα between 67NR and 4T1.2 and between EO771 and EO771.LMB was retained when the cells were cultured *in vitro* (Fig. 5C).

### Gene expression signatures in mouse mammary tumours

To delineate molecular features of the four tumour models (EO771/EO771.LMB and 67NR/4T1.2), whole primary tumours were subjected to array-based gene expression profiling and gene set enrichment analysis (GSEA), completed by applying eight different gene expression signatures relevant to breast cancer biology (supplementary material Tables S1–S3; Fig. S9A–H). According to this genomic analysis, a basal cell expression signature was not enriched in any of the tumours (supplementary material Fig. S9A; Table S1); however, a luminal expression signature was unexpectedly enriched in 4T1.2 tumours (supplementary material Fig. S9B). EO771 and EO771.LMB tumours both displayed enrichment of proliferation genes (supplementary material Fig. S9C), hypoxia-regulated genes (supplementary material Fig. S9D) and upregulation of a subset of interferon-regulated genes (supplementary material Fig. S9E), thus distinguishing them from tumours of the 4T1 model. None of the tumours was significantly enriched for invasion, epithelial-to-mesenchymal transition (EMT) or breast cancer stem cell signatures (supplementary material Fig. S9F–H), indicating that these latter three phenotypes are not the sole determinants of metastatic capacity.

### Gene expression profiling identifies an association of MMP-3, Pthlh and S100a8 with the metastatic phenotype

To identify candidate genes that might be causal in metastatic dissemination, the array data were analysed for transcripts commonly upregulated or downregulated in both 67NR/4T1.2 and EO771/EO771.LMB isogenic pairs (supplementary material Fig. S10; Tables S2, S3). Candidate genes common to both pairs were selected for further interrogation based on fold change, *P*-value and extent of differential expression in the EO771/EO771.LMB pair. Differential gene expression was confirmed by qRT-PCR in whole tumours (Table 1; supplementary material Fig. S11).

**Table 1.**
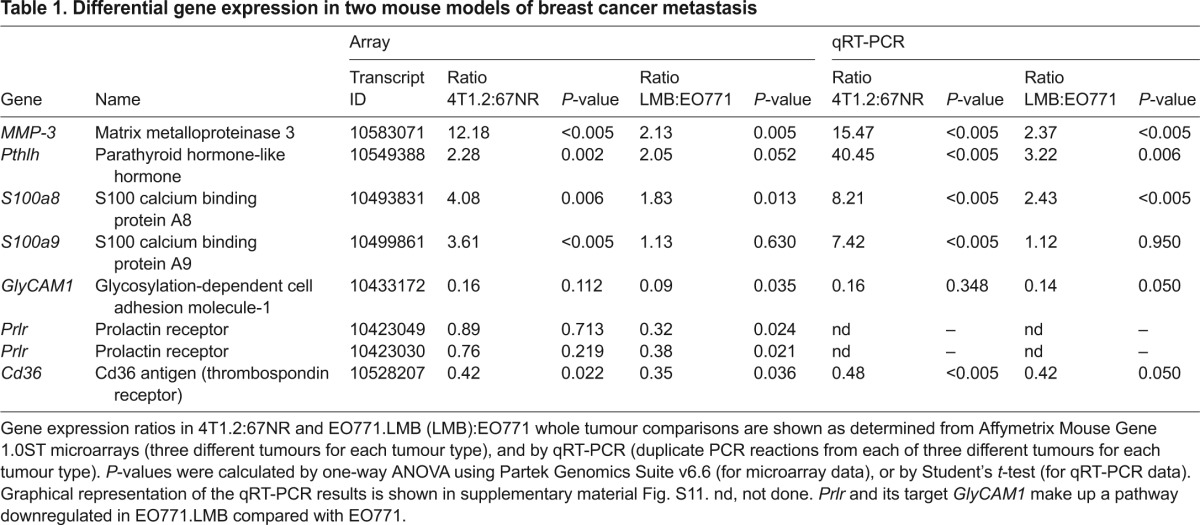
Differential gene expression in two mouse models of breast cancer metastasis

Matrix metalloproteinase-3 (*MMP-3*) and parathyroid hormone-like hormone (*Pthlh*) genes encode secreted factors implicated previously in breast cancer progression (Kremer et al., 2011; Sternlicht et al., 1999). These two genes were upregulated in 4T1.2 and EO771.LMB primary tumours compared with their non-metastatic counterparts, by both microarray and qRT-PCR. The secreted neutrophil chemotactic factor calprotectin is a stable heterodimer of the related S100A8 and S100A9 proteins, although each protein is also able to form homodimers (Ehrchen et al., 2009). Both proteins are co-expressed in human breast cancers, where they are associated with aggressive tumour characteristics (Acharyya et al., 2012; Arai et al., 2008; Moon et al., 2008). Secretion of S100a8 by primary tumours has also been implicated in the formation of the pre-metastatic niche in mouse lung (Hiratsuka et al., 2008). S100a8 was elevated in both 67NR/4T1.2 and EO771/EO771.LMB tumour comparisons, although differential expression of S100a9 was found only in 4T1.2/67NR (Table 1; supplementary material Fig. S11). Two genes downregulated in metastatic tumours were also evaluated. Cd36 is a scavenger receptor for oxidized low-density lipoprotein (LDL) found on the surface of myeloid cells, erythrocytes, endothelium and adipocytes in the tumour microenvironment (Koch et al., 2011). It binds collagen and thrombospondin (Koch et al., 2011), and its loss in tumour stroma is associated with poor outcome in breast cancer (Defilippis et al., 2012). Cd36 was downregulated approximately twofold in whole metastatic tumours from each model (Table 1; supplementary material Fig. S11). Glycosylation-dependent cell adhesion molecule-1 (GlyCAM1) is a secreted proteoglycan found in high endothelial venules of lymph nodes and in murine mammary epithelium during pregnancy and lactation (Hou et al., 2000). GLYCAM1 expression has not been evaluated in human breast cancer (Lister et al., 1998). *GlyCAM1* mRNA levels were strongly reduced in the more metastatic variants of both isogenic pairs but did not reach significance in 67NR/4T1.2 (Table 1; supplementary material Fig. S11). Interestingly, GlyCAM1 is induced by prolactin in murine mammary epithelium (Hou et al., 2000). Accordingly, prolactin receptor (Prlr) expression was also significantly reduced in EO771.LMB compared with EO771 (Table 1), indicating that attenuated Prlr signalling might be responsible for the lower GlyCAM1 levels found in EO771.LMB.

**Fig. 6.**
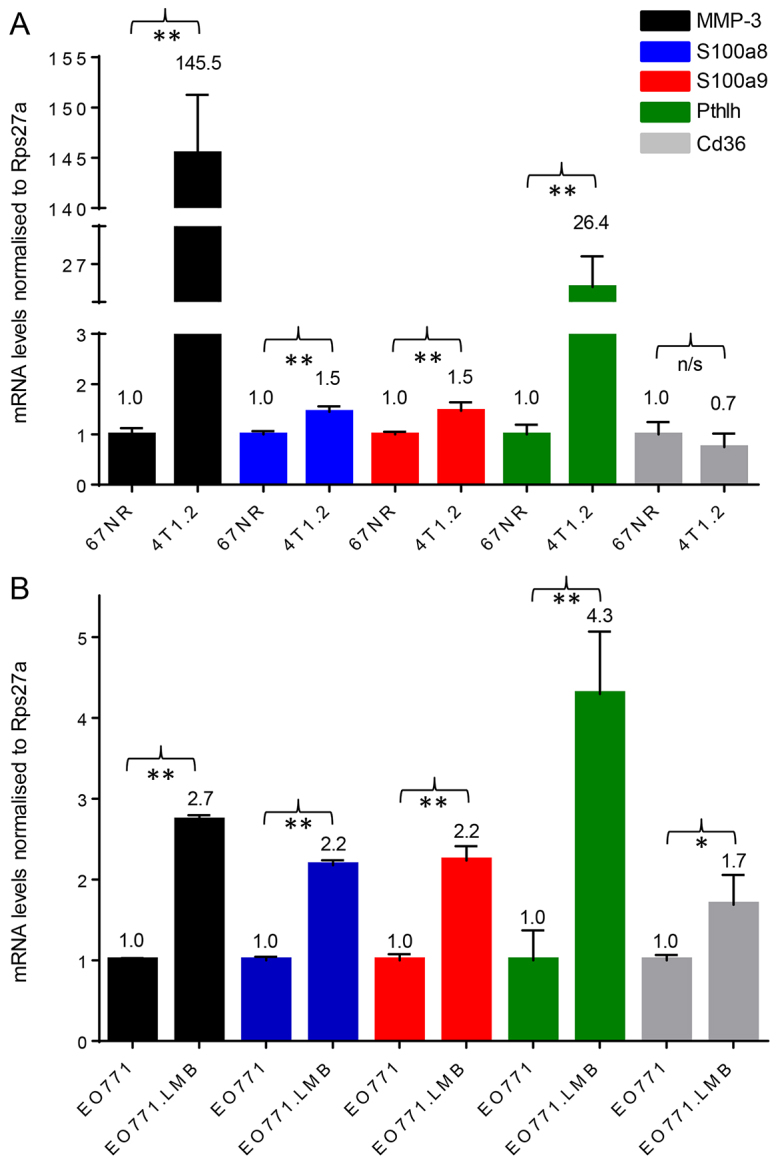
**qRT-PCR analysis of *MMP-3*, *S100a8*, *S100a9*, *Pthlh* and *Cd36* mRNA levels in isolated primary tumour cells.** RNA was isolated from the primary tumour epithelium of two mice for each tumour type and pooled. Each data point represents the mean ± s.d. of triplicate reactions. For the pairwise comparisons, gene expression levels in non-metastatic 67NR and EO771 were set to 1 for each gene analysed. (A) 67NR and 4T1.2. (B) EO771 and EO771.LMB. *P<0.05; **P<0.01; n/s, not significant.

Because differential gene expression was shown in whole tumours, it was unclear whether these genes were expressed by the tumour cells, by the surrounding stroma, or by both. To check this, we evaluated expression levels of selected genes in adenocarcinoma cells isolated directly from the primary tumours by flow cytometry. MMP-3, S100a8, S100a9 and Pthlh transcripts were all upregulated significantly in isolated primary tumour cells of the metastatic variants in both pairs (Fig. 6), reflecting the deregulation observed in whole tumours. However, Cd36 levels were not significantly downregulated in the more metastatic tumour cells (Fig. 6). Thus, the reduction found in whole tumour Cd36 expression is likely to be due to deregulation in the stroma, as suggested previously (DeFilippis et al., 2012).

To determine whether differential gene expression in tumour epithelium requires the unique surroundings of the tumour microenvironment, transcript levels were also measured in cultured cells. As observed for primary tumour cells, upregulation of MMP-3 and S100a8 was also found in the *in vitro* comparisons of both 67NR/4T1.2 and EO771/EO771.LMB (supplementary material Fig. S12), indicating that expression of these genes in tumours is regulated in a cell autonomous way and that tumour epithelium might be the major site of expression *in vivo*. However, the upregulation of Pthlh and S100a9, and the downregulation of Cd36, observed in primary tumour epithelium were not found consistently in cultured tumour cells from both of the isogenic non-metastatic/metastatic pairs (supplementary material Fig. S12).

Genes upregulated in the metastatic murine tumours were evaluated for association with patient outcome in a collection of human breast cancer datasets (Madden et al., 2013). Higher levels of MMP-9, S100A8 and S100A9 transcripts were each significantly associated with worse relapse-free survival over a 20-year interval when all breast cancers were considered (supplementary material Fig. S13). However, none of these genes reached statistical significance when only basal breast cancers were analysed. On the other hand, although MMP-3 was not prognostic when all breast cancers were considered, it was prognostic of poor outcome in basal-like breast cancers (supplementary material Fig. S13). Expression of PTHLH was not associated with patient survival (data not shown).

## DISCUSSION

A range of mouse models of breast cancer that incorporate appropriate stromal elements, immune surveillance and spontaneous metastasis from the mammary gland to distant organs are required for preclinical testing of novel therapeutics that prevent and/or target secondary tumours. Here, we report the establishment of a new syngeneic model of breast cancer metastasis, EO771.LMB in immunocompetent C57BL/6 mice, a background that is inherently resistant to metastasis. Functional assays indicated that the underlying reason for the increased metastatic proclivity of EO771.LMB compared with EO771 was the acquisition of enhanced matrix-dependent invasive ability. EO771.LMB expressed higher levels of MMP-9 and MMP-3 that have been shown to facilitate tumour cell invasion (Bernhard et al., 1994; Huang et al., 2009).

We compared the functional and molecular characteristics of EO771 with those of the well-established 4T1 model. Only 67NR displayed nuclear ERα and an inhibitory response to tamoxifen both *in vitro* and *in vivo*. Despite the lack of nuclear ERα, EO771 primary tumours, but not cells in culture, also responded to tamoxifen. We therefore conclude that tamoxifen might be acting through modulation of ERα activity in the tumour microenvironment in these tumours, as reported previously (Gupta et al., 2007; Pontiggia et al., 2012; Ribas et al., 2011). In luminal human breast cancer, ERα is usually co-expressed with PR (Stierer et al., 1993), and HER2 expression is usually absent. However, all four mouse tumours were negative for both PR and Erb-b2/Neu protein. Therefore, the immunohistochemical data indicate that EO771, EO771.LMB and 4T1.2 are of the core basal phenotype, defined as triple-negative tumours expressing either CK5/6 and/or EGFR (Blows et al., 2010). The 67NR tumour is classified as luminal 1 (similar to luminal A), as defined by Blows et al. (Blows et al., 2010). On the other hand, our genomic analysis demonstrated that only 18% of the human luminal gene signature was significantly upregulated in 67NR tumours, thereby providing discordance between the immunohistochemical classification and the genomic classification. Furthermore, none of the three tumours classified as core basal by immunohistochemistry showed enrichment of basal-like gene expression by GSEA. Despite harbouring a likely point mutation in *p53*, which is associated with a basal-like phenotype in human tumours, the basal-like genes were no more enriched in EO771 and EO771.LMB than in 67NR or 4T1.2. Therefore, taken together, the data show that EO771, EO771.LMB and 4T1.2 are predominantly basal-like, and that 67NR is predominantly luminal-like. An additional point of distinction is that 67NR primary tumours have a moderate level of nuclear pleomorphism, whereas each of the other three tumour types contained high-grade nuclei.

Several studies of human breast cancer also report discordance between immunohistochemical and transcriptional profiles. For example, Gazinska et al. reported that, of 142 triple-negative breast cancers, 116 basal-like cancers were identified by one of three classifiers (histology, immunohistochemistry or PAM50 gene signature), but only 13 cancers were classified as basal-like by all three methods (Gazinska et al., 2013). Hence, depending on the classification method used, the subtyping of breast cancers can vary. Discordance between ERα expression and assessment of molecular subtype by RNA-based methods has also been observed in human breast tumours, with between 1 and 3% of ERα-positive tumours displaying a basal-like phenotype (Perou et al., 2000; Sørlie et al., 2001; Sorlie et al., 2003). Similarly, up to 60% of tumours classified as HER2-positive by immunohistochemistry were classified otherwise by gene expression profiling (de Ronde et al., 2010).

Recent studies have indicated that human basal-like tumours can contain at least three different subgroups, including ‘claudin-low’, ‘molecular apocrine’ and ‘interferon-rich’. Owing to an upregulation of interferon-inducible genes, including *Ifit1*, *Ifit3* and *Bst2*, the EO771 and EO771.LMB tumours might correspond more closely to the interferon-rich subtype of human basal-like tumours (Hu et al., 2006; Teschendorff et al., 2007). The results of these analyses are broadly in agreement with previous studies of transgenic mouse models of breast cancer that found conservation of some but not all features of the principal human breast cancer molecular subtypes (Herschkowitz et al., 2007; Pfefferle et al., 2013). In summary, the molecular subtyping of breast cancers based on their transcriptome varies with the intrinsic gene list or with the single sample predictors used for the classification (Weigelt et al., 2010). For current clinical use, histological features of tumours combined with immunohistochemical assessment of ERα, PR and HER2 are still the main classifiers used to decide on treatment options.

Integrated genomic analysis of microarray data from primary tumours of the two different isogenic pairs revealed that *MMP-3*, *Pthlh* and *S100a8* were commonly upregulated and that *Cd36* was commonly downregulated, indicating that these genes might be causal in tumour cell dissemination. All four genes were expressed by the tumour cells and are also likely to be expressed by several stromal cell lineages in the tumour microenvironment. Elevated expression of MMP-3, Pthlh and S100a8 was confirmed in isolated primary tumour epithelial cells of the two metastatic variants, as was expression of S100a9, a binding partner for S100a8. MMP-3 and S100a8 were also upregulated in the metastatic cells *in vitro*. However, it is likely that stromal cells contribute to *in vivo* expression as well, especially for S100a8 and S100a9. As well as contributing to the recruitment of neutrophils and myeloid-derived suppressor cells (MDSCs) into tumours (Ehrchen et al., 2009), the S100A8-S100A9 heterodimer (calprotectin) is synthesized by MDSCs and regulates their functions (Acharyya et al., 2012; Lukanidin and Sleeman, 2012). MDSC-derived calprotectin enhances survival of human MDA-MB-231 breast cancer xenografts in a paracrine manner at metastatic sites (Acharyya et al., 2012), and elevated levels of calprotectin in lung metastases of breast cancer patients is associated with a worse overall survival (Acharyya et al., 2012). Both S100a8 homodimers and calprotectin signal via Toll-like receptor-4 (Tlr4) (Vogl et al., 2007), consistent with expression of Tlr4 in all four tumour lines (data not shown). Therefore, tumour-cell-derived S100a8/a9 proteins are likely to signal in both autocrine and paracrine ways in these tumours.

Pthlh was upregulated in the metastatic tumours from both models. *PTHLH* encodes a 36-amino-acid mature peptide strongly implicated in multiple aspects of breast cancer progression. However, its roles are complex and yet to be completely elucidated. A germline polymorphism near *PTHLH* was associated with increased risk for the development of both sporadic (Ghoussaini et al., 2012) and *BRCA1*-mutation-associated breast cancer (Antoniou et al., 2012), and tumour expression of PTHLH promotes the formation of osteolytic lesions in bone (Guise et al., 1996; Sloan and Anderson, 2002). However, whereas *Pthlh* activity promoted primary tumour growth and metastatic dissemination in the MMTV-PyMT transgenic mouse model of breast cancer (Li et al., 2011), it delayed tumour initiation in the MMTV-Neu model (Fleming et al., 2009). PTHLH expression in primary human tumours was also reported to be associated with ERα positivity and reduced incidence of bone metastasis (Henderson et al., 2006), as well as improved overall survival (Henderson et al., 2006; Surowiak et al., 2003). However, other reports have indicated that primary-tumour PTHLH-positivity is associated with poorer disease-free survival (Linforth et al., 2002; Yoshida et al., 2000), an effect that is enhanced if co-expressed with the PTHLH receptor (Linforth et al., 2002). The function of PTHLH as either a promoter or attenuator of cancer progression might depend on the nature of the processed PTHLH peptides present in the tumour milieu. Interestingly, a recent report showed that MMP-3, known to have broad substrate specificity (Coussens and Werb, 1996), is capable of processing pro-PTHLH into its mature form as well as further cleaving mature PTHLH into smaller bioactive fragments (Frieling et al., 2012). EO771.LMB cells produce higher MMP-9 levels than do EO771, and we have shown previously that 4T1.2 cells have higher levels of MMP-9 than do 67NR (Tester et al., 2000). MMP-3 is also able to process pro-MMP-9 into its enzymatically active form (Ramos-DeSimone et al., 1999). Thus, MMP-3 might promote spontaneous metastasis in 4T1.2 and EO771.LMB tumours in part via proteolytic processing of PTHLH and MMP-9. Indeed, exposure of mammary epithelial cells to MMP-3 results in the induction of epithelial-to-mesenchymal transition (Lochter et al., 1997a; Lochter et al., 1997b; Radisky et al., 2005; Sternlicht et al., 1999), invasion (Lochter et al., 1997a; Lochter et al., 1997b; Sternlicht et al., 1999), genomic instability (Radisky et al., 2005) and transformation (Lochter et al., 1997a; Sternlicht et al., 1999).

In summary, the EO771-derived isogenic model of spontaneous breast cancer metastasis described here and the 4T1 model described primarily in previous publications (Eckhardt et al., 2005; Lelekakis et al., 1999) have enabled the identification of genes and molecular pathways that might regulate metastasis in two different strains of mice. These tumour models display features of both luminal and basal-like cancers, demonstrating their phenotypic diversity as is seen also in human breast cancer. Because no single model accurately depicts human breast cancer, a diversity of syngeneic preclinical models is required for a more comprehensive analysis of metastasis-regulating genes and for testing new therapies that target metastatic disease. We have provided a characterisation of the EO771 metastasis model in C57BL/6 mice that can be used in addition to other models to improve our ability to understand metastasis and develop therapies for individuals with advanced breast cancer.

## MATERIALS AND METHODS

### Cell lines and cell culture

The EO771 cell line was derived from a spontaneous mammary tumour in a C57BL/6 mouse (Casey et al., 1951) and was stored in liquid nitrogen vapour phase. Early-passage parental EO771 cells were transduced with the pMSCV (murine stem cell virus) retroviral vector expressing the mCherry fluorescent protein (Denoyer et al., 2011). The lungs from a mouse orthotopically implanted with EO771_mCherry cells in our laboratory were excised and sorted by flow cytometry for mCherry-positive cells that were expanded in culture. This sequence of orthotopic growth *in vivo* followed by recovery of mCherry-positive cells from the lung was repeated. Upon the second round of mammary fat-pad injections of these mCherry-positive cells, visible lung nodules were detected. One of these nodules was designated Lung Metastasis nodule B and, after being returned to culture, became the EO771.LMB cell line. 67NR and 4T1 cell lines were derived from a subpopulation of a single mammary tumour that arose in a BALB/c/C3H mouse (Aslakson and Miller, 1992), with the 4T1.2 cell line being derived from a single-cell clone of the 4T1 population (Lelekakis et al., 1999). EMT6.5 (Ellis et al., 2000) is a single-cell clone derived from the EMT6 mammary tumour (Rockwell and Kallman, 1973). NMuMG immortal murine mammary epithelial cells were obtained from ATCC. The *Pik3ca*-mutant murine mammary tumour line MH248 was a kind gift from Dr Wayne Phillips (Tikoo et al., 2012). The murine mammary tumour line AT3, derived from polyoma-middle T antigen transgenic mice (Stewart and Abrams, 2007), was a kind gift from Dr Trina Stewart (Griffith University, Queensland, Australia). 67NR and 4T1.2 mammary adenocarcinoma cells were maintained in Eagle’s minimum essential medium (alpha modification) supplemented with 5% (v/v) fetal bovine serum (FBS) (SAFC Biosciences, Brooklyn, Victoria, Australia) and 1% (v/v) penicillin-streptomycin, whereas EO771, EO771.LMB, EMT6.5, AT3, MH248 and NMuMG cells were maintained in Dulbecco’s modified Eagle’s medium (DMEM) containing HEPES (20 mM) supplemented with 10% (v/v) FBS, penicillin (100 IU/ml) and streptomycin (100 μg/ml). All cells were cultured at 37°C in 5% CO_2_ (v/v) in air and were maintained in culture for a maximum of 4-5 weeks.

### Adhesion assay

Short-term adhesion assays (30 minutes) were completed using the calcein-AM (Life Technologies, Mulgrave, Victoria, Australia) labelling method as described previously (Chia et al., 2007). The experiment was repeated three times with results showing the mean of triplicate wells ± standard deviation (s.d.) of a representative experiment.

### Proliferation assay

Proliferation assays were completed using the sulforhodamine B (SRB) colorimetric assay as described previously (Vichai and Kirtikara, 2006). Cells were seeded into 96-well plates at an initial density of 1×10^3^ cells/well. Proliferation was also assessed in the presence of 500 nM 4-hydroxytamoxifen (4-HT) dissolved in ethanol and diluted to a final ethanol concentration of 1% (v/v).

### Migration and invasion assays

Migration and invasion assays were run in triplicate porous (8-μm pore size) Transwell migration chambers (BD Biosciences, Bedford, MA, USA) as described previously (Chia et al., 2007; Kusuma et al., 2012; Sloan et al., 2006). Transwells were coated with ECM proteins overnight at 4°C (Chia et al., 2007). Recombinant human laminin-511 (alpha5beta1gamma1) was isolated as previously described (Doi et al., 2002), and vitronectin was obtained from Sigma. For migration assays, cells (2×10^5^/200 μl) in serum-free medium (SFM) were seeded into the top chamber of the Transwell. For invasion assays, a cell suspension of 1×10^5^ cells in 50 μl of SFM was mixed with 50 μl Matrigel (BD Biosciences). 80 μl of the mixture was placed in the Transwell and allowed to set for 30 minutes, followed by addition of 100 μl of SFM. Cells were allowed to migrate for 4–5 hours or invade for 18 hours. The data represent the mean number of migrated or invaded cells ± s.d. of a representative experiment (*n*=3).

### Gelatin zymography

Gelatinase assays were completed as described previously (Kusuma et al., 2011), with minor modifications. Briefly, the cells (5×10^5^/400 μl SFM) were incubated for 24 hours at 37°C and secreted proteins in the supernatants separated by SDS-PAGE on 8% polyacrylamide gels supplemented with 1% bovine gelatin. Proteolytic digestion occurred over the next 24 hours. Gels were scanned and clear MMP-2 and MMP-9 signals quantitated by densitometry using ImageJ software (NIH).

### UV irradiation and western blotting

Cultured cells were exposed to 7 J/m^2^ ultraviolet C (UVC) or mock-exposed and whole-cell lysates prepared 4 hours later. Lysates were separated by SDS- PAGE, transferred to a nitrocellulose membrane, and incubated overnight with anti-p53 antibody (1:500, CM5p, Novocastra, Leica Microsystems, North Ryde, NSW, Australia). Blots were stripped and re-probed with a mouse monoclonal anti-α-tubulin antibody (1:10,000, T5168, Sigma). Bands were visualised using a horseradish peroxidase (HRP)-conjugated secondary antibody and an enhanced chemiluminescence-based detection system.

### Quantitative real-time RT-PCR

A representative sample of primary tumour was lysed in Trizol reagent using a FastPrep automated bench-top homogeniser (MP Biomedical, Seven Hills, NSW, Australia), and total RNA isolated in accordance with the manufacturer’s instructions (Life Technologies). Total RNA was subsequently re-purified using RNeasy mini-columns with on-column DNaseI digestion (Qiagen, Doncaster, Victoria, Australia). For cell lines, total RNA was isolated using RNeasy mini-kits with on-column DNaseI digestion (Qiagen). RNA quality was determined using an RNA6000 Nano chip and 2100 Bioanalyzer (Agilent Technologies, Santa Clara, CA, USA). cDNA was synthesised using SuperscriptIII reverse transcriptase (Life Technologies). qPCR was completed using either inventoried TaqMan gene expression assays (ERα, ERβ, PR, Erb-b2, Cd36, MMP-3, Glycam1) in a 15 μl reaction volume (Life Technologies) or using SYBR-green reagent (Life Technologies) in a 15 μl reaction volume in conjunction with the primers listed in supplementary material Table S4, as previously described (Johnstone et al., 2004). Rps27a was used as an internal reference gene for all reactions. PCR reactions were run for 45 cycles in 96-well plates using a StepOne-Plus real-time PCR platform (Life Technologies).

### Primary tumour cell sorting by flow cytometry

The mCherry-expressing 67NR, 4T1.2, EO771 and EO771.LMB primary tumours were disaggregated by collagenase I digestion (Worthington Biochemical Corporation, Lakewood, NJ, USA) and filtered through a series of sieves prior to sorting for mCherry-positive cells using a FACSDiva cell sorter (BD Biosciences). SYTOX green (Invitrogen) was used to exclude non-viable cells.

### Immunohistochemistry

Tissue sections (6 μm) were stained using a standard protocol. Briefly, slides were heated for antigen retrieval by pressure cooker treatment in 0.01 M sodium citrate buffer, pH 6.0 (125°C for 3 minutes, 90°C for 10 seconds). Sections were blocked in 3% (v/v) normal goat serum in 0.05% (v/v) PBS-Tween 20 for 1 hour at room temperature. Primary antibody incubation was conducted in blocking buffer overnight at 4°C. Non-specific rabbit IgG (Dako, Campbellfield, Victoria, Australia) or mouse IgG (Dako) antibodies were used as isotype controls. Biotin-conjugated goat anti-rabbit or anti-mouse secondary antibodies (Dako) were used at 1:250 or 1:300 dilution for 1 hour at room temperature. Specific primary-secondary antibody complexes were detected using ABC reagent (Vector Laboratories, Burlingame, CA, USA) and visualised using a 3, 3′-diaminobenzidine peroxidase substrate kit (Vector Laboratories). Sections were counterstained with hematoxylin, dehydrated and mounted. The primary antibodies and dilutions used were as follows: mouse monoclonal anti-human ERα (1:100, clone 1D5, Dako), chicken polyclonal anti-human ERβ (1:500, a kind gift from Dr Jan-Åke Gustafsson, University of Houston, TX, USA), rabbit polyclonal anti-human PR (1:4500, sc-538, Santa Cruz Biotechnology, Dallas, TX, USA), rabbit polyclonal anti-human HER2 (1:400, A0485, Dako), mouse monoclonal anti-human cytokeratin 5/6 (KRT5/6, 1:100, clone D5/16 B4, Merck Millipore, Kilsyth, Victoria, Australia), rabbit polyclonal anti-EGFR (1:50, ab2430, Abcam, Cambridge, UK) and rabbit polyclonal anti-mouse p53 (1:300, CM5p, Novocastra).

### Tumour growth and analysis

Female BALB/c or C57BL/6 mice (obtained from Walter and Eliza Hall Institute of Medical Research, Parkville, Victoria, Australia) were maintained in a specific pathogen-free environment and fed *ad libitum.* All procedures involving mice conformed to National Health and Medical Research Council animal ethics guidelines and were approved by the Animal Experimentation and Ethics Committee (AEEC) of the Peter MacCallum Cancer Centre. To generate primary tumours, 1×10^5^ cells were implanted into the fourth inguinal mammary gland [in 20 μl of Hank’s balanced salt solution (HBSS)] of 8- to 10-week-old female BALB/c (67NR, 4T1.2 or EMT6.5) or C57BL/6 (EO771 or EO771.LMB) mice. Primary tumour volume was measured three times per week using electronic callipers. The greatest longitudinal diameter (length) and the greatest transverse diameter (width) were measured. Tumour volumes were estimated by the modified ellipsoidal formula: volume=1/2(length×width^2^) (Tomayko and Reynolds, 1989). In resection experiments, primary tumours were excised at a size of 400–600 mm^3^. For experiments involving tamoxifen administration (Xianju Green Leaf Pharmaceutical Factory, Zhejiang, China), mice were administered tamoxifen (10 μg tamoxifen citrate per gram of chow), commencing on the day of tumour cell implantation. Differences in primary tumour growth rates were calculated by determining the area under each curve using the trapezoid rule (Atkinson, 1989), and then comparing the area under the curve values using a two-sample Student’s *t*-test (Bryant, 1983).

### Experimental lung metastasis assay

EO771 or EO771.LMB cells (5×10^5^ cells in 200 μl of HBSS) were injected into C57BL/6 mice via the tail vein using a 26 gauge needle. 19 days later the metastatic burden in the lung was analysed by TaqMan qPCR.

### Analysis of lung metastatic burden

Dissected lungs were stained with India Ink to visualise metastatic nodules and the number of surface nodules enumerated. Alternatively, metastatic burden in lung was determined by TaqMan qPCR-based detection of the mCherry nucleotide sequence in whole lung genomic DNA as described previously (Denoyer et al., 2011; Eckhardt et al., 2005).

### Anchorage independent growth in soft agar

Soft agar assays were conducted in six-well plates as previously described (Mongroo et al., 2004). Colonies were stained with calceinAM dye (Enzo Life Sciences, Farmingdale, NY, USA) and fluorescent images generated (32× magnification) using an Olympus fluorescent dissecting stereomicroscope. The number of colonies >50 μm in size were counted in three fields per well and averaged.

### Generation of mammospheres

Mammosphere cultures were conducted using serum-free DMEM:Ham’s F12 medium containing bFGF, EGF and B27 supplement (Life Technologies) as previously described (Dontu et al., 2003; To et al., 2010). Briefly, 2000 single and viable cells in 2 ml of medium were seeded in triplicate into six-well ultra-low-attachment plates (Corning, In Vitro Technologies, Noble Park North, Victoria, Australia) and primary mammospheres allowed to form over 10 days. After analysis, mammospheres were dissociated into single cells using 0.1% (2×) Trypsin-EDTA at 37°C for 15 min and cell viability determined by Trypan Blue exclusion. Single cells were again seeded into triplicate wells as above and secondary mammospheres allowed to form over 7 days. Mammospheres were imaged using a Leica DM IRB inverted microscope (50× magnification). Only mammospheres with a diameter of >150 μm were counted. Mammosphere area was determined using ImageJ software (NIH).

### Xenograft tumour growth

One million MCF-7 or MDA-MB-231 human breast cancer cells were inoculated into the 4th mammary gland of NOD scid gamma (NSG) mice and allowed to grow for 6 weeks. Mice implanted with MCF-7 cells were supplemented with 1 μM 17β-estradiol in the drinking water *ad libitum*. Tumours were fixed overnight in 10% neutral buffered formalin prior to processing for immunohistochemistry.

### Gene expression profiling

Total RNA was isolated from primary tumours using the procedure described above. In solution DNaseI digestion (TURBO DNase, Ambion, Life Technologies) was completed on a portion of the total RNA, followed by verification of RNA integrity using a RNA6000 Nano chip and 2100 Bioanalyzer (Agilent Technologies). RNA was processed using NuPAGE reagents (Affymetrix, Santa Clara, CA, USA) and applied to GeneChip Mouse Gene 1.0ST Arrays as per the manufacturer’s instructions (Affymetrix). Three different tumours were processed per tumour type (15 tumours in total). A confocal scanner was used to acquire the fluorescence signal after excitation at 570 nm. Background adjustment, quantile normalization and median-polish summarization was completed using the GC Robust Multi-array Average (GCRMA) algorithm. Microarray profiling data were deposited into the Gene Expression Omnibus (G.E.O.) with Accession No. GSE42272 (http://www.ncbi.nlm.nih.gov/geo/).

### Bioinformatics

Affymetrix.cel files were obtained and differential gene expression between 4T1.2/67NR and EO771.LMB/EO771 calculated by one-way ANOVA using Partek Genomics Suite v6.6 (Partek Inc., St Louis, MO, USA). Gene set enrichment analysis was completed using the R program (http://www.R-project.org/). Firstly, the mean expression level of each gene was calculated across 15 different tumours (three each from 67NR, 4T1.2, EO771, EO771.LMB and EMT6.5). Then, the number of genes within each signature that were significantly upregulated or significantly downregulated (*P*<0.05) in 67NR, 4T1.2, EO771 or EO771.LMB tumours was calculated relative to the mean expression level of each gene across all 15 tumours analysed. Gene expression signatures were obtained from the Molecular Signatures Database (http://www.broadinstitute.org/gsea/msigdb/index.jsp) or from the following references: basal epithelial (54 genes) (Huper and Marks, 2007), luminal epithelial (59 genes) (Huper and Marks, 2007), proliferation (97 genes) (Ghazoui et al., 2011), hypoxia-regulated (75 genes) (Dowsett et al., 2011), core EMT markers (91 genes) (Taube et al., 2010), interferon-regulated (27 genes) (Einav et al., 2005), breast cancer stem cells (93 genes) (Creighton et al., 2009) and cancer cell invasion (64 genes) (Kim et al., 2010).

## Supplementary Material

Supplementary Material
