# Supplementary material for: Functional and molecular characterisation of EO771.LMB tumours, a new C57BL/6-mouse-derived model of spontaneously metastatic mammary cancer
Source: Dis Model Mech. 2015 Jan 29;8(3):237–51. doi: 10.1242/dmm.017830 (PMC4348562; doi:10.1242/dmm.017830)
Supplement: Supplementary Material [file supp_8_3_237__index.html]

Functional and molecular characterisation of EO771.LMB tumours, a new C57BL/6-mouse-derived model of spontaneously metastatic mammary cancer — Supplementary Material 

# Functional and molecular characterisation of EO771.LMB tumours, a new C57BL/6-mouse-derived model of spontaneously metastatic mammary cancer

## DMM017830 Supplementary Material

**Files in this Data Supplement:**

- **Supplementary Material**
