# Supplementary material for: Functional and molecular characterisation of EO771.LMB tumours, a new C57BL/6-mouse-derived model of spontaneously metastatic mammary cancer
Source: Dis Model Mech. 2015 Jan 29;8(3):237–51. doi: 10.1242/dmm.017830 (PMC4348562; doi:10.1242/dmm.017830)
Supplement: Supplementary Material [file supp_8.3.237_DMM017830.pdf]

Supplementary Figure Legends

Figure S1: Histological classification of murine mammary tumours. (A) EO771 and EO771.LMB primary tumours were sectioned (3 µm), stained with haematoxylin and eosin (H&E), and compared to 67NR and 4T1.2 primary tumours. H&E stained sections from at least two primary tumours of each tumour type were assessed by a qualified breast pathologist in accordance with the Bloom Richardson Ellis classification system (Elston and Ellis, 2002). Circles indicate highly pleomorphic nuclei. (B) An overall grade was calculated dependent on tubular differentiation/formation (score 1: >75% of tumor area forming glandular/tubular structures, score 2: 10% to 75% and score 3: <10%), nuclear pleomorphism (score 1: nuclei with little increase in size in comparison with normal breast epithelium with minimal variation in size, score 2: larger nuclei with moderate variability and visible nucleoli and score 3: marked nuclear variation with prominent nucleoli) and tumour mitotic count determined by numbers of mitosis seen in 10 consecutive high power fields (score 1: <9 mitosis per 10 HPF, score 2: 9-17 mitosis per 10 HPF, score 3: 18 or more mitosis per 10 HPF). Adding the scores for each parameter determined tumour grade as follows: grade 1 - scores 3,4 and 5, grade 2 - scores 6 and 7, grade 3 - scores 8 and 9.

Black scale bars represent 100 µm. White circles denote highly pleomorphic nuclei.

Figure S2: Whole lungs were dissected from mice inoculated with the indicated tumour lines and stained with India ink. The number of metastatic nodules on the surface of each lung was counted. $P = 0.20$ (Mann-Whitney test).

Figure S3: Experimental lung metastasis assay. EO771 or EO771.LMB cells were inoculated into C57Bl/6 mice via the tail vein. 19 days later, the metastatic burden in the lung was analyzed by TaqMan qPCR. $P = 0.51$ (Student's t test).

Figure S4: The cell lines indicated were cultured on plastic or monomeric type I bovine collagen (PureCol, Advanced Biomatrix, Carlsbad, CA) for at least 2 days. Images (x100 magnification) were generated using a Leica DM IRB inverted microscope (Leica Microsystems, North Ryde, NSW, Australia).

Figure S5: Anchorage independent growth in soft agar. Colonies in soft agar were stained with calcein^{AM} and fluorescent images generated. The number of colonies > 50 µm in size were counted in three fields per well and averaged. The graph depicts the average number of colonies per field across three wells ± SD. Differences in colony forming ability between EO771 and EO771.LMB were not significant (Student's t test).

Figure S6: Mammosphere forming capacity of EO771 and EO771.LMB cells in vitro. Representative images from primary (A) and secondary (B) mammosphere cultures from EO771 and EO771.LMB (LMB) are shown. Scale bar = 200 µm. (C) Mammospheres from primary or secondary cultures were enumerated after 10 or 7 days of growth, respectively, and expressed as the mean number of mammospheres per 10 fields ± SEM. No significant differences in mammosphere number was found between EO771 and EO771.LMB in either primary or secondary cultures. (D) The graph depicts mean mammosphere area in μm^2 ± SEM for primary and secondary cultures after 10 or 7 days of growth, respectively. The number of individual

mammospheres measured in each condition is indicated above the bars. n/s, not significant.

Figure S7: Adhesion of 67NR and 4T1.2 tumour cells to different substrates after 30min. Adhesion is presented as the percentage of total cell input (mean of triplicate wells ± SD of one of three representative experiments). *, $P < 0.05$.

Figure S8: ERα-positive human breast cancer cells (MCF7, top panels) or ERα-negative human breast cancer cells (MDA-MB-231, bottom panels) were grown as xenografts in the mammary glands of immunodeficient mice. Tumour sections were stained with anti-human ERα antibody (left panels) or an isotype control antibody (right panels). Scale bars represent 100μm.

Figure S9: Distribution of eight gene expression signatures across 67NR, 4T1.2, EO771, and EO771.LMB primary tumours displayed as heat maps. Triplicate tumour specimens are indicated along the X axes and individual genes are shown on the Y axes. Gene expression signatures were obtained from the following references or from the Molecular Signatures Database (http://www.broadinstitute.org/gsea/msigdb/index.jsp). (A) Basal epithelial (54 genes) (Huper and Marks, 2007), (B) luminal epithelial (59 genes) (Huper and Marks, 2007), (C) proliferation (97 genes) (Ghazoui et al., 2011), (D) hypoxia-regulated (75 genes) (Ghazoui et al., 2011), (E) interferon-regulated (27 genes) (Einav et al., 2005), (F) cancer invasion (64 genes) (Kim et al., 2010), (G) EMT (91 genes) (Taube et al., 2010), (H) breast cancer stem cells (94 genes) (Creighton et al., 2009).

Figure S10: Venn diagrams were generated from Affymetrix array data showing genes significantly (unadjusted $P < 0.05$) upregulated **(A)**, or downregulated **(B)** in 4T1.2 v 67NR (red) and EO771.LMB v EO771 (green). *P* values were determined by one-way ANOVA using Partek Genomics Suite v6.6. 205 genes were commonly upregulated and 220 genes commonly downregulated in both isogenic tumour comparisons.

Figure S11: qRT-PCR data showing mRNA expression levels of MMP-3 (A), Pthrp (B), S100a8 (C), S100a9 (D), Cd36 (E) and GlyCAM1 (F) in whole 67NR, 4T1.2, EO771 and EO771.LMB (LMB) primary tumours. Three different primary tumours were analysed in duplicate for each tumour model. Thus, each data point represents the mean ± SD of 6 qRT-PCR reactions across 3 tumours. Expression levels in normal adult mouse mammary gland (MG) were included for each gene for comparison and set to 1. Values on the Y axis represent mRNA expression levels of the gene of interest normalised to Rps27a. *, $P = 0.05$; **, $P < 0.01$; ***, $P < 0.005$; n/s, not significant.

Figure S12: qRT-PCR data showing mRNA expression levels of MMP-3 (A), Pthrp (B), S100a8 (C), S100a9 (D), Cd36 (E) and GlyCAM1 (F) in 67NR, 4T1.2, EO771 and EO771.LMB (LMB) cell cultures. Each data point represents the mean ± SD of triplicate qRT-PCR reactions. Expression levels in the immortalised mouse mammary epithelial cell line NMuMG were included for comparison and set to 1. Values on the Y axis represent mRNA expression levels of the gene of interest normalised to Rps27a. *, $P < 0.05$; **, $P < 0.01$; n/s, not significant; n/e, not expressed.

Figure S13: Kaplan-Meier survival curves (disease-free survival) for selected genes in primary human breast cancer samples. The BreastMark database (http://glados.ucd.ie/BreastMark/index.html) was analyzed for the individual genes indicated above the curves for all patients (all, upper row) or in basal-like tumours only (basal, lower row). Gene expression was measured in primary tumours using Affymetrix microarrays (Madden et al., 2013) A median split was used to allocate 50% of tumours to the "high expression" category (blue curve) and 50% to the "low expression" category (red curve). Patient clinical follow-up was for at least 200 months (X axes). A logrank P value ≤ 0.05 was considered statistically significant. s, significant, n/s, not significant.

Supplementary Table 1: Eight different expression signatures were analysed across four murine mammary cancer models (67NR, 4T1.2, EO771, EO771.LMB) using the R program. The Table depicts the number of genes and percentage of genes (in parentheses) within each signature that were significantly ($P < 0.05$) upregulated (UP), or significantly ($P < 0.05$) downregulated (DOWN) in 67NR, 4T1.2, EO771, or EO771.LMB tumours relative to the mean expression level of each gene across all 15 tumours analyzed (see Materials and Methods). The number and percentage of genes within each signature that were not significantly deregulated is also indicated. Yellow highlighting indicates tumour types where ≥ 25% of the genes within the signature were significantly upregulated, and were thereby considered enriched for the signature. The enriched gene expression signatures are summarised at the bottom of the Table.

Supplementary Table 2: Genes significantly **downregulated** (unadjusted *P* < 0.05, n=220) in both 4T1.2 v 67NR and EO771.LMB v EO771 comparisons. *P* values were determined by one-way ANOVA using Partek Genomics Suite v6.6.

Supplementary Table 3: Genes significantly **upregulated** (unadjusted *P* < 0.05, n=205) in both 4T1.2 v 67NR and EO771.LMB v EO771 comparisons. *P* values were determined by one-way ANOVA using Partek Genomics Suite v6.6.

Supplementary Table 4: Sequences of oligonucleotide primers used for SYBR-green qRT-PCR.

Supplementary References

Creighton, C. J., Li, X., Landis, M., Dixon, J. M., Neumeister, V. M., Sjolund, A., Rimm, D. L., Wong, H., Rodriguez, A., Herschkowitz, J. I. et al. (2009). Residual breast cancers after conventional therapy display mesenchymal as well as tumor-initiating features. *Proc. Natl. Acad. Sci. U. S. A.* **106**, 13820-5.

Einav, U., Tabach, Y., Getz, G., Yitzhaky, A., Ozbek, U., Amariglio, N., Izraeli, S., Rechavi, G. and Domany, E. (2005). Gene expression analysis reveals a strong signature of an interferon-induced pathway in childhood lymphoblastic leukemia as well as in breast and ovarian cancer. *Oncogene* **24**, 6367-75.

Elston, C. W. and Ellis, I. O. (2002). Pathological prognostic factors in breast cancer. I. The value of histological grade in breast cancer: experience from a large study with long-term follow-up. *Histopathology* **41**, 154-61.

Ghazoui, Z., Buffa, F. M., Dunbier, A. K., Anderson, H., Dexter, T., Detre, S., Salter, J., Smith, I. E., Harris, A. L. and Dowsett, M. (2011). Close and stable relationship between proliferation and a hypoxia metagene in aromatase inhibitor-treated ER-positive breast cancer. *Clin. Cancer Res.* **17**, 3005-12.

Huper, G. and Marks, J. R. (2007). Isogenic normal basal and luminal mammary epithelial isolated by a novel method show a differential response to ionizing radiation. *Cancer Res.* **67**, 2990-3001.

Kim, H., Watkinson, J., Varadan, V. and Anastassiou, D. (2010). Multi-cancer computational analysis reveals invasion-associated variant of desmoplastic reaction involving INHBA, THBS2 and COL11A1. *BMC Med. Genomics* **3**, 51.

Madden, S. F., Clarke, C., Gaule, P., Aherne, S. T., O'Donovan, N., Clynes, M., Crown, J. and Gallagher, W. M. (2013). BreastMark: an integrated approach to mining publicly available transcriptomic datasets relating to breast cancer outcome. *Breast Cancer Res.* **15**, R52.

Taube, J. H., Herschkowitz, J. I., Komurov, K., Zhou, A. Y., Gupta, S., Yang, J., Hartwell, K., Onder, T. T., Gupta, P. B., Evans, K. W. et al. (2010). Core epithelial-to-mesenchymal transition interactome gene-expression signature is associated with claudin-low and metaplastic breast cancer subtypes. *Proc. Natl. Acad. Sci. U. S. A.* **107**, 15449-54.

A

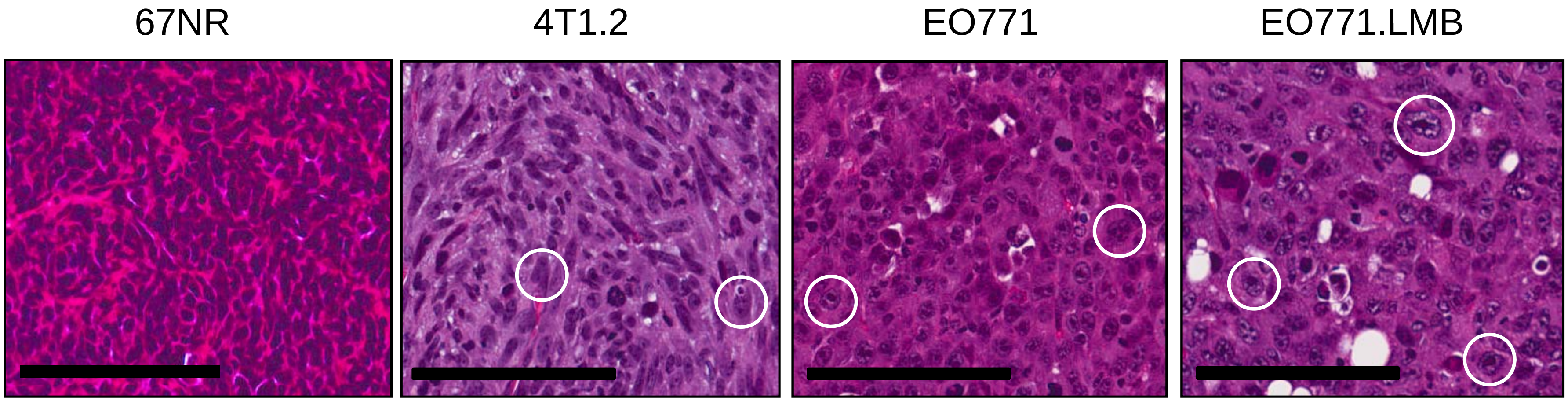

B

Tumour	Overall Grade	Tubule Formation Score	Nuclear Pleomorphism Score	Mitosis Score	Mitoses per 10 HPF
67NR-A	3	3	2	3	29
67NR-B	3	3	2	3	32
4T1.2-A	3	3	3	2	15
4T1.2-B	3	3	3	3	42
**EO771-A**	3	3	3	3	27
**EO771-B**	3	3	3	3	90
**EO771.LMB-A**	3	3	3	3	57
**EO771.LMB-B**	3	3	3	3	66

Supp. Figure 1

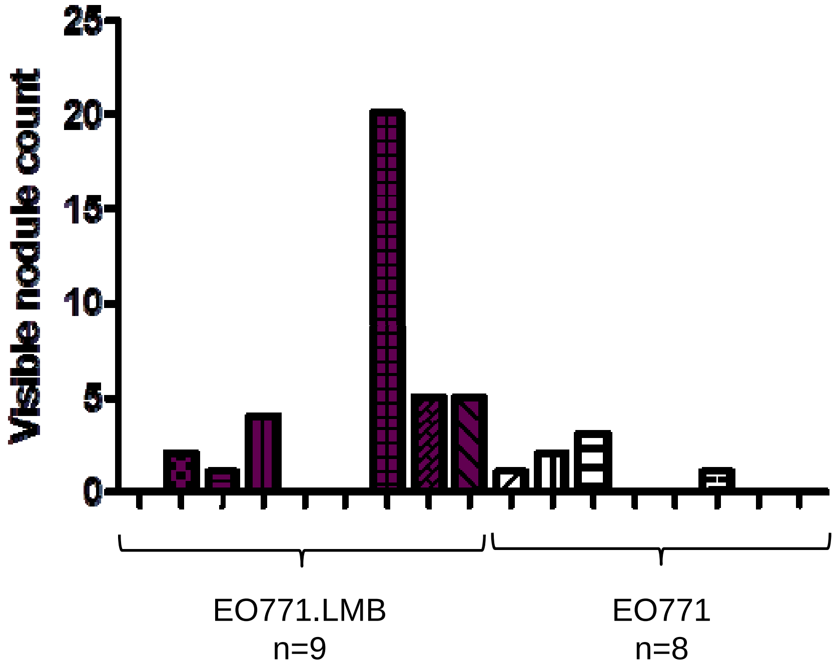

P = 0.20

Supp. Fig. 2

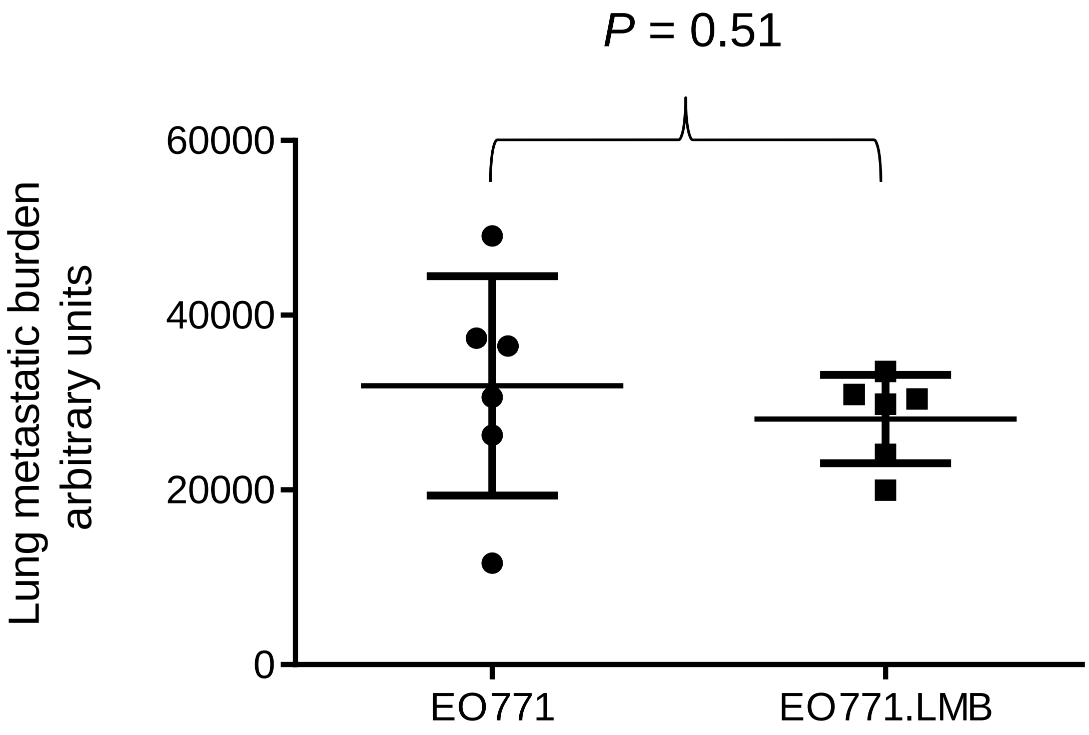

Supp. Figure 3

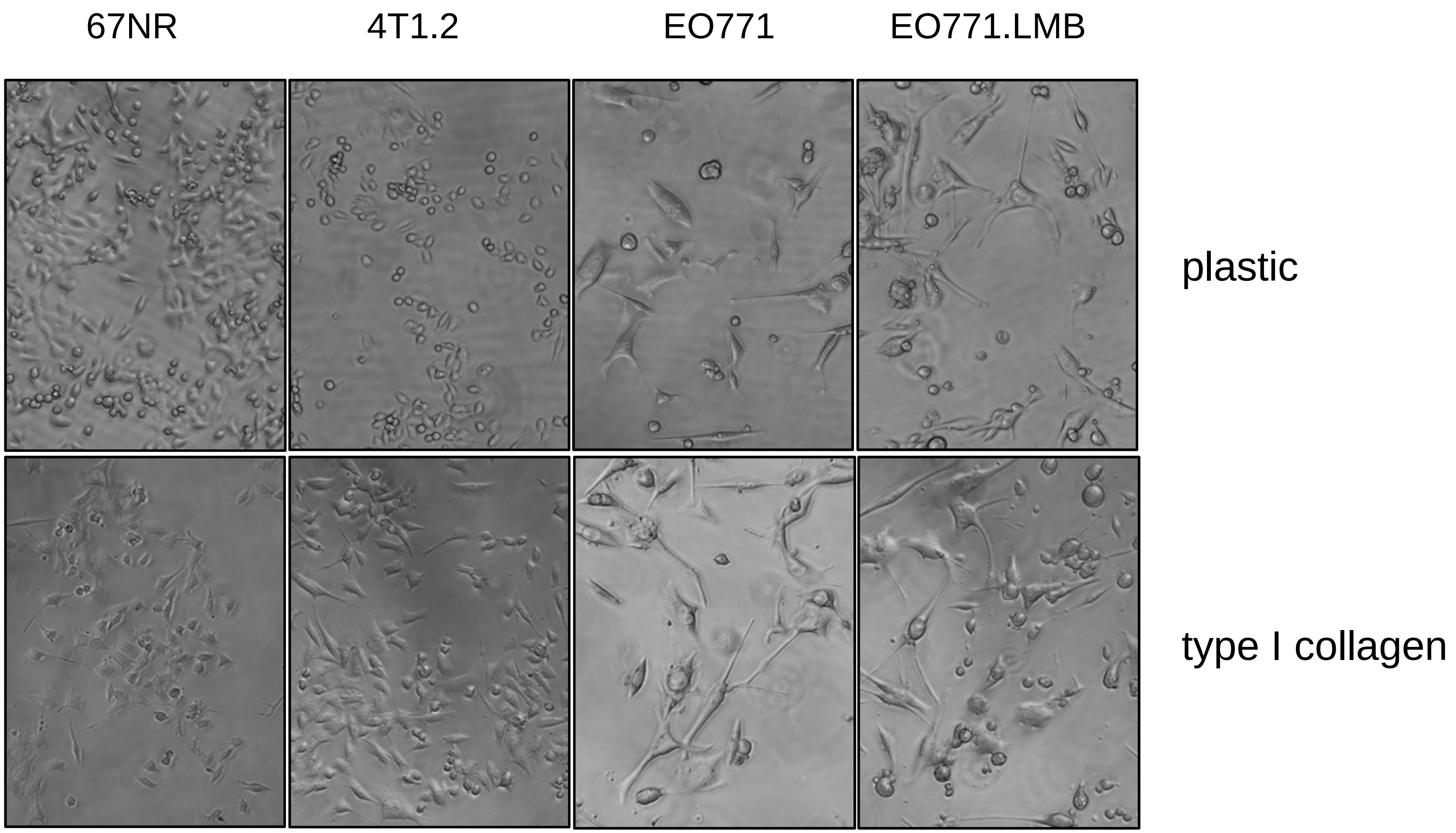

Supp. Figure 4

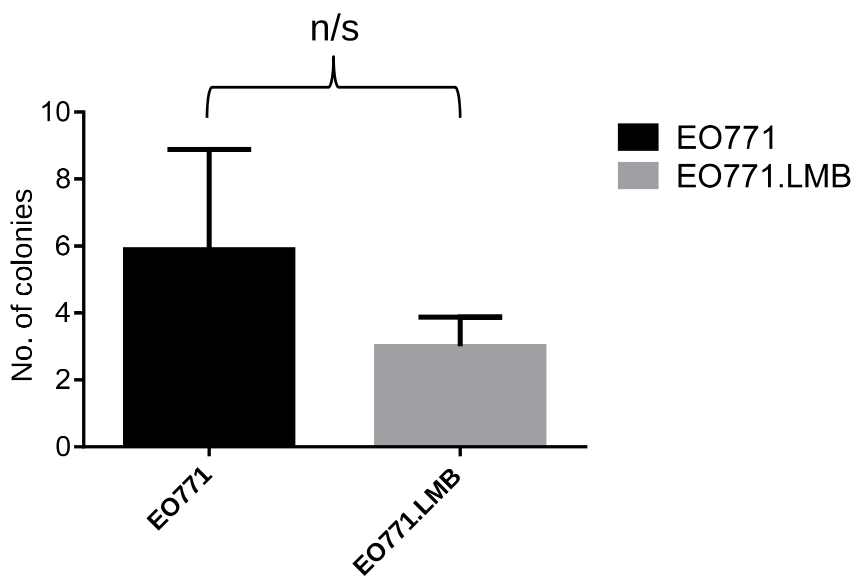

Supp. Figure 5

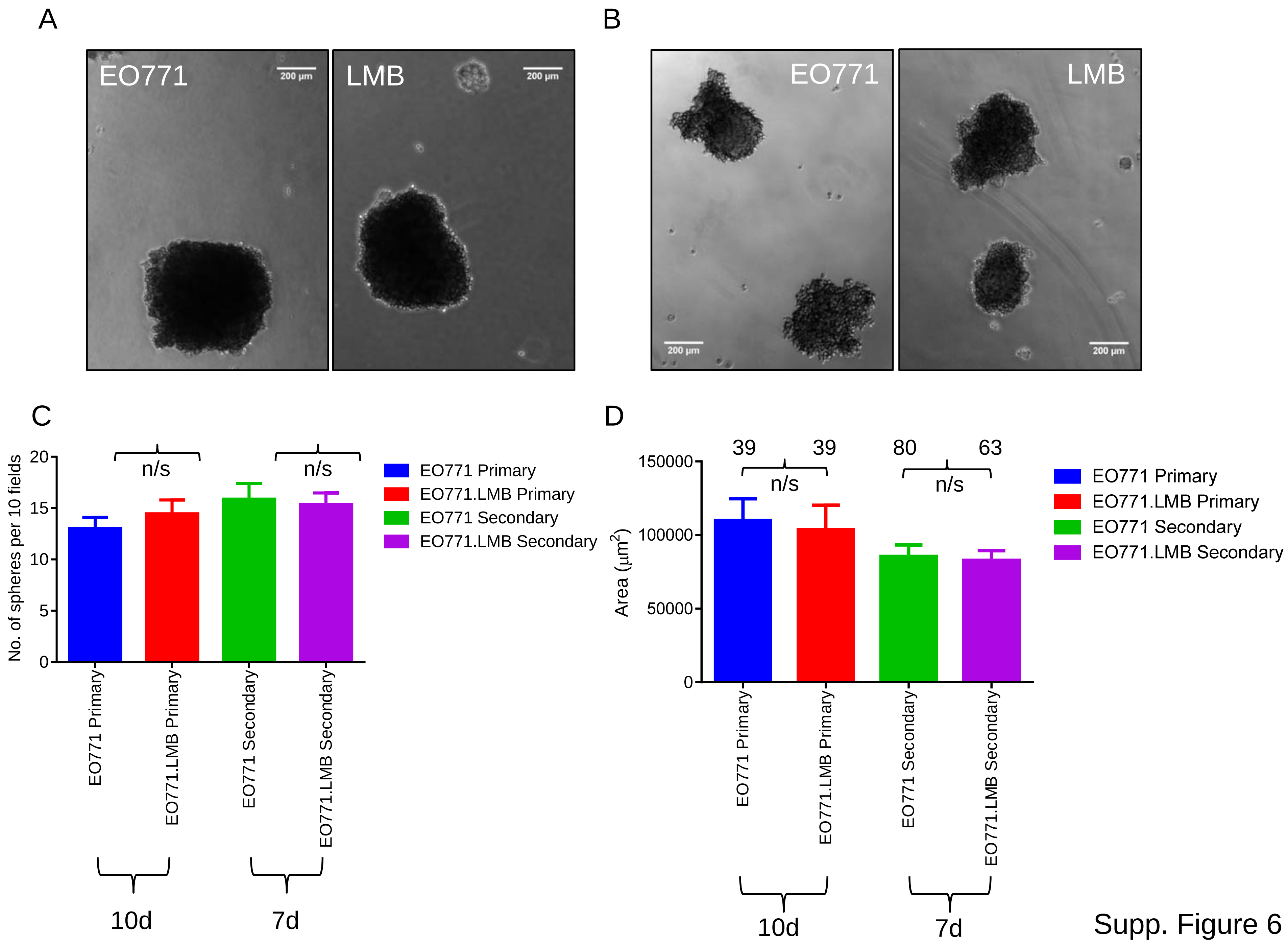

Supp. Figure 6

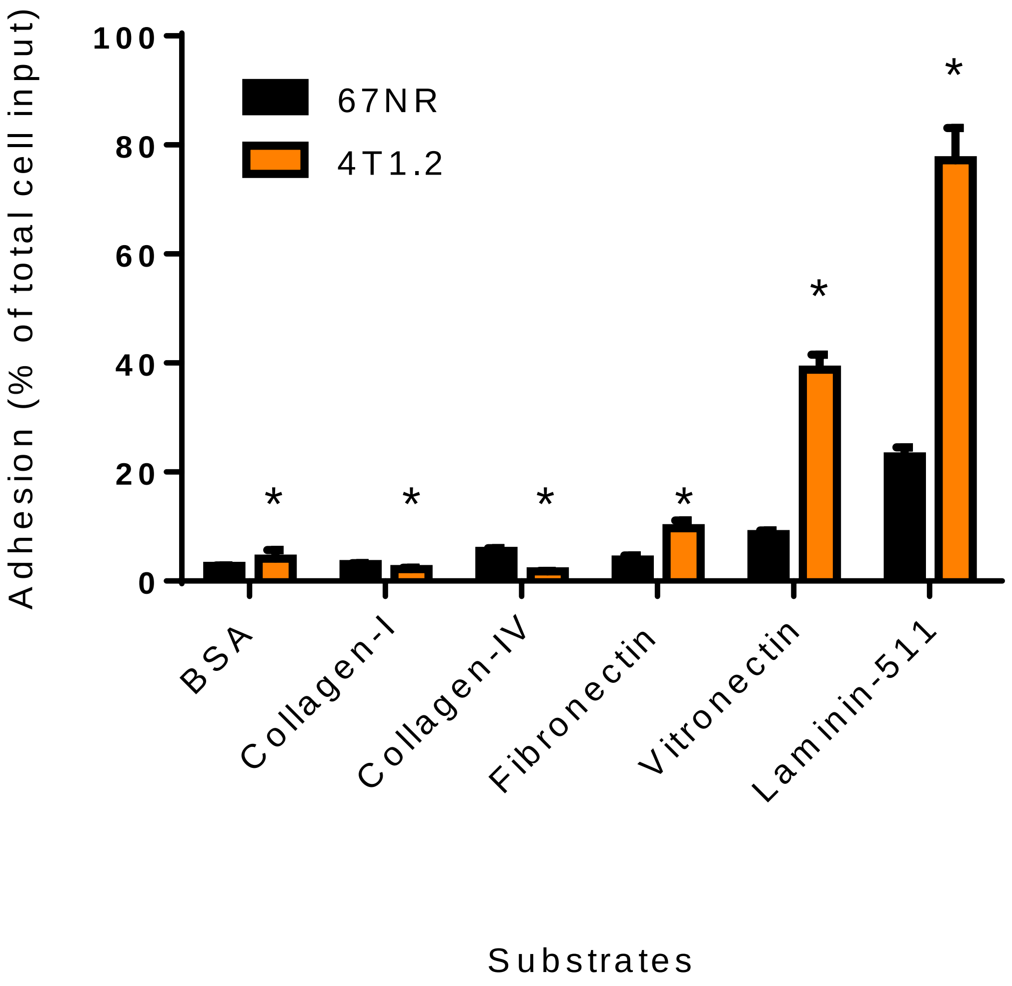

Supp. Figure. 7

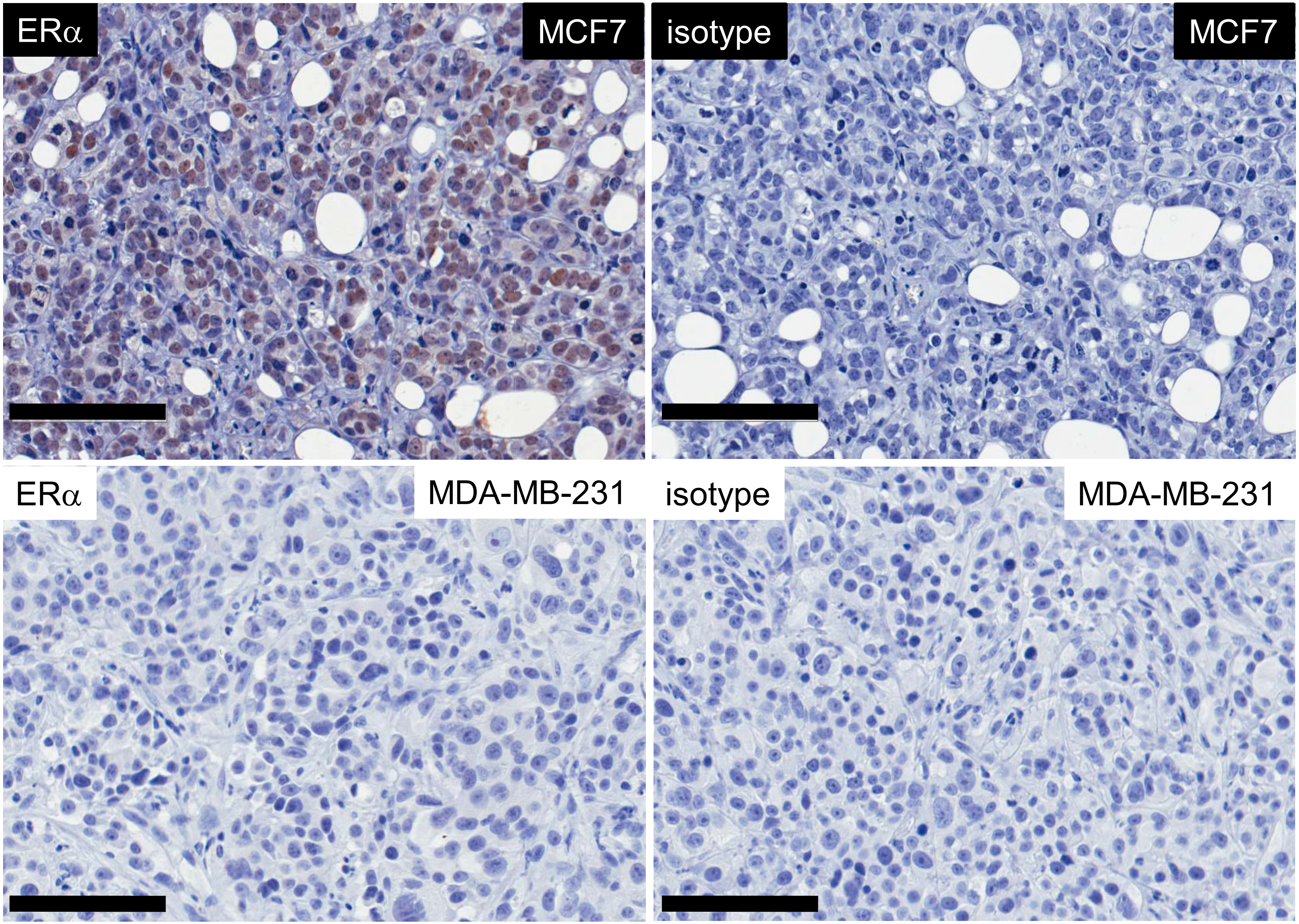

Supplementary Figure 8

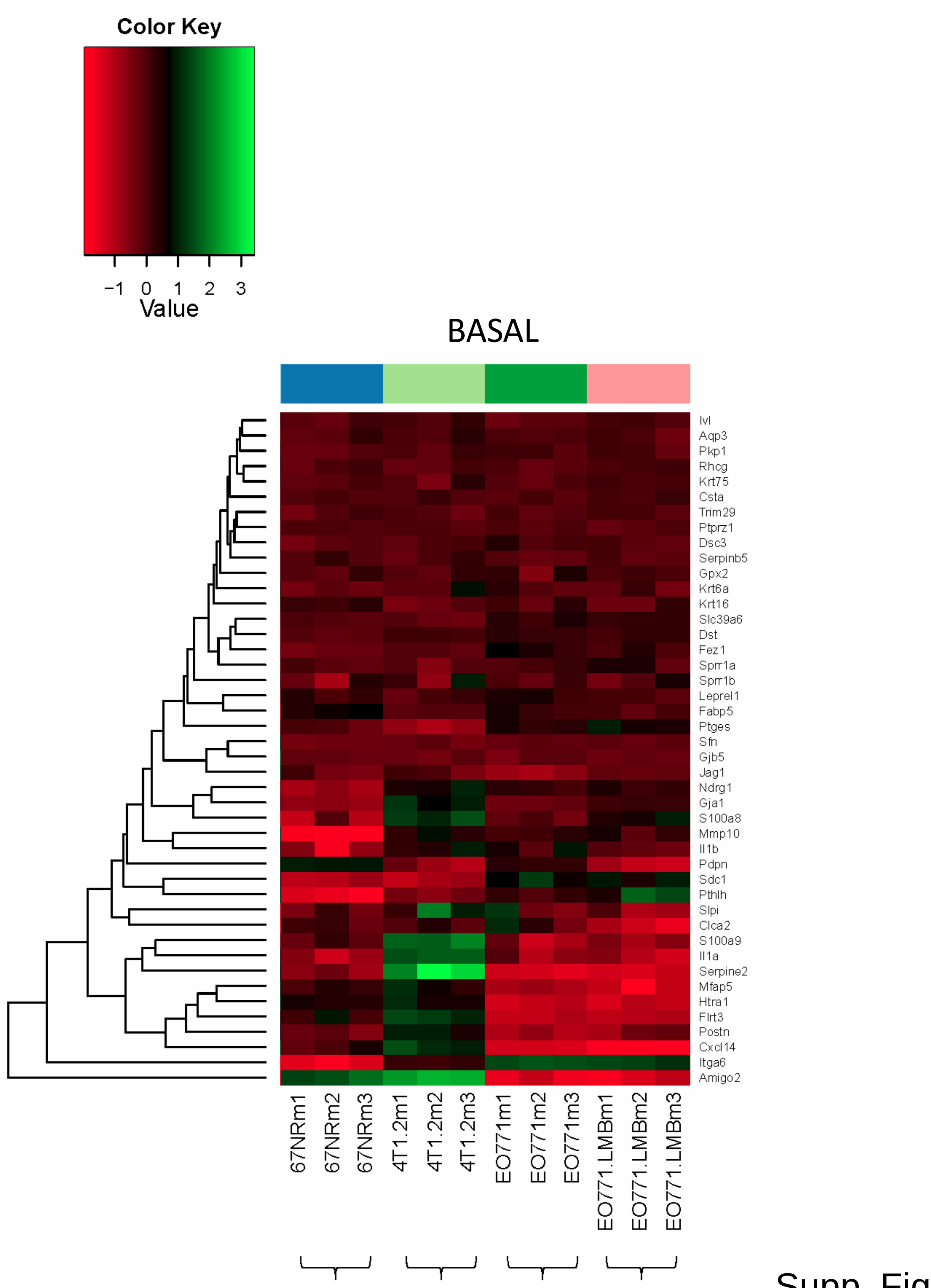

Supp. Figure 9A

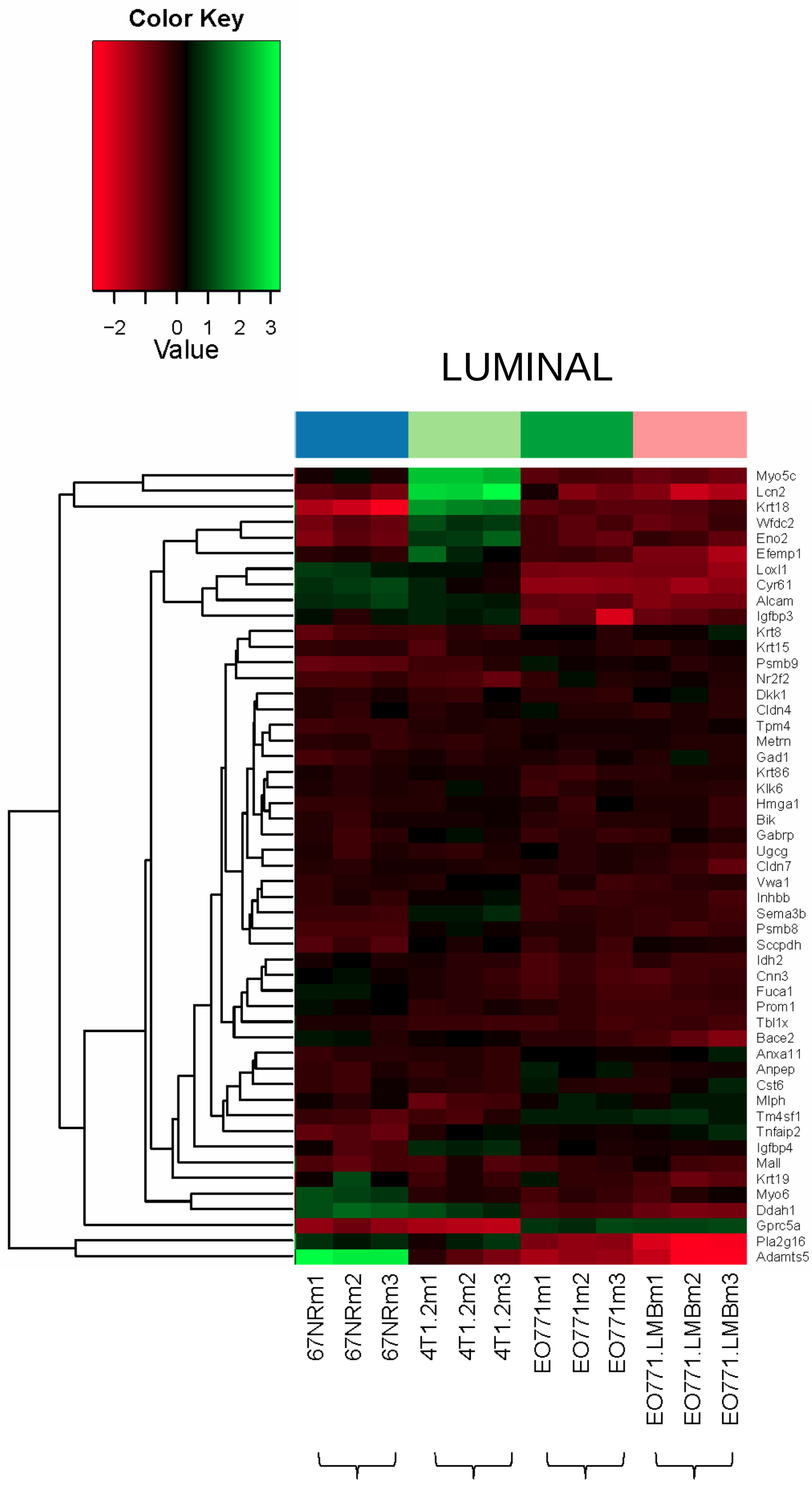

Supp. Figure 9B

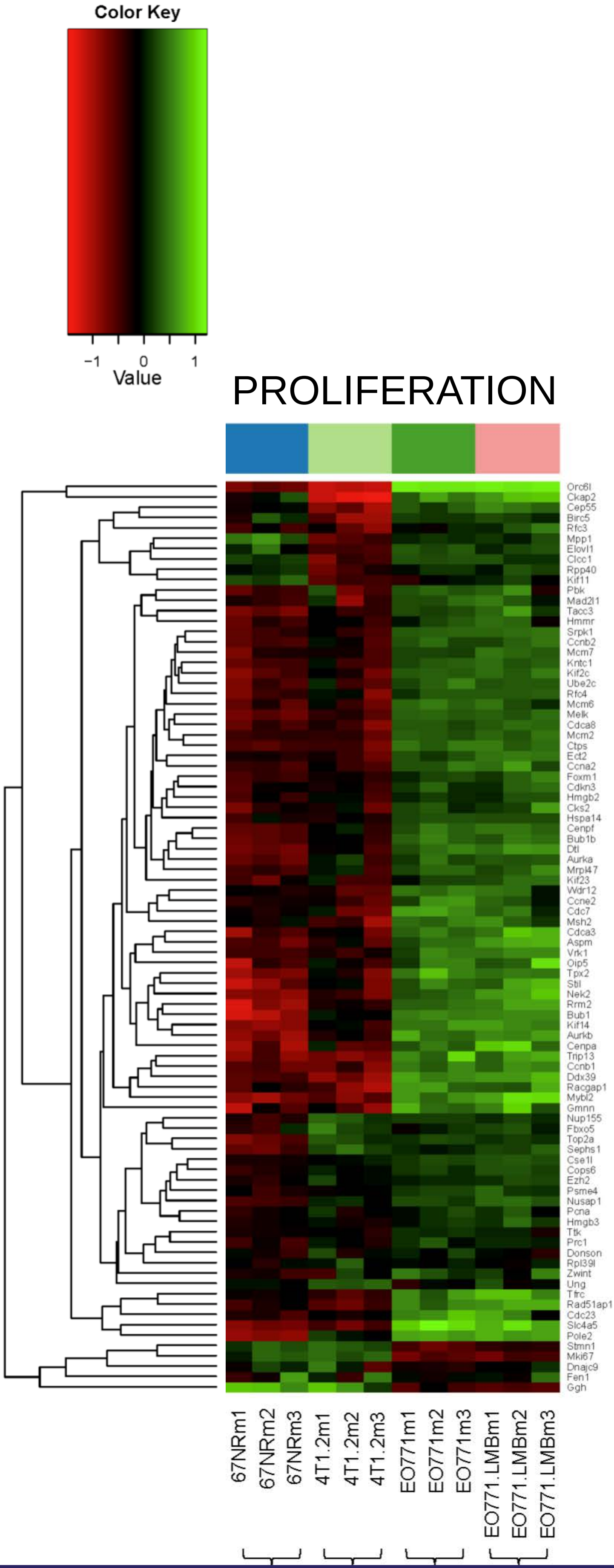

Supp. Figure 9C

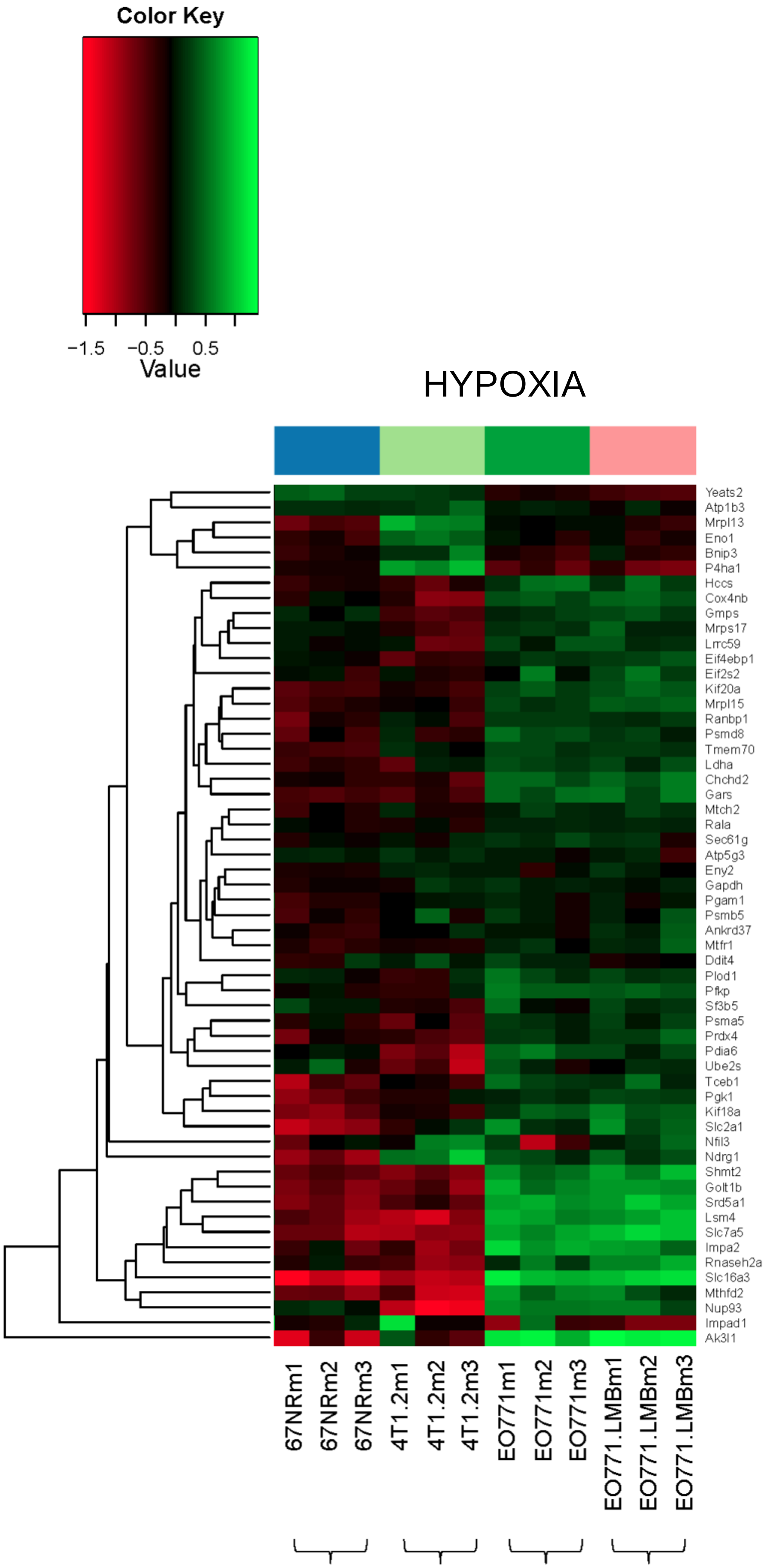

Supp. Figure 9D

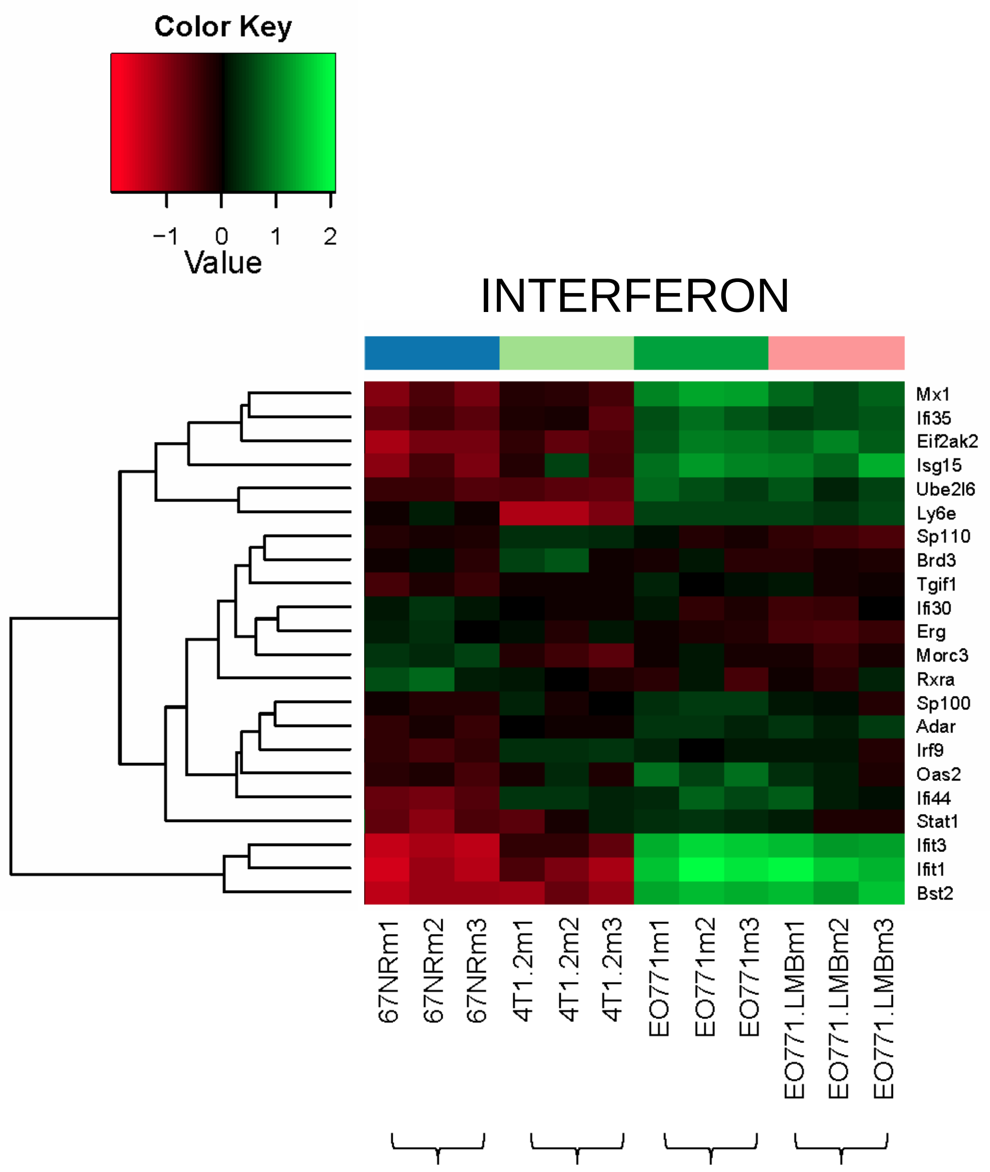

Supp. Figure 9E

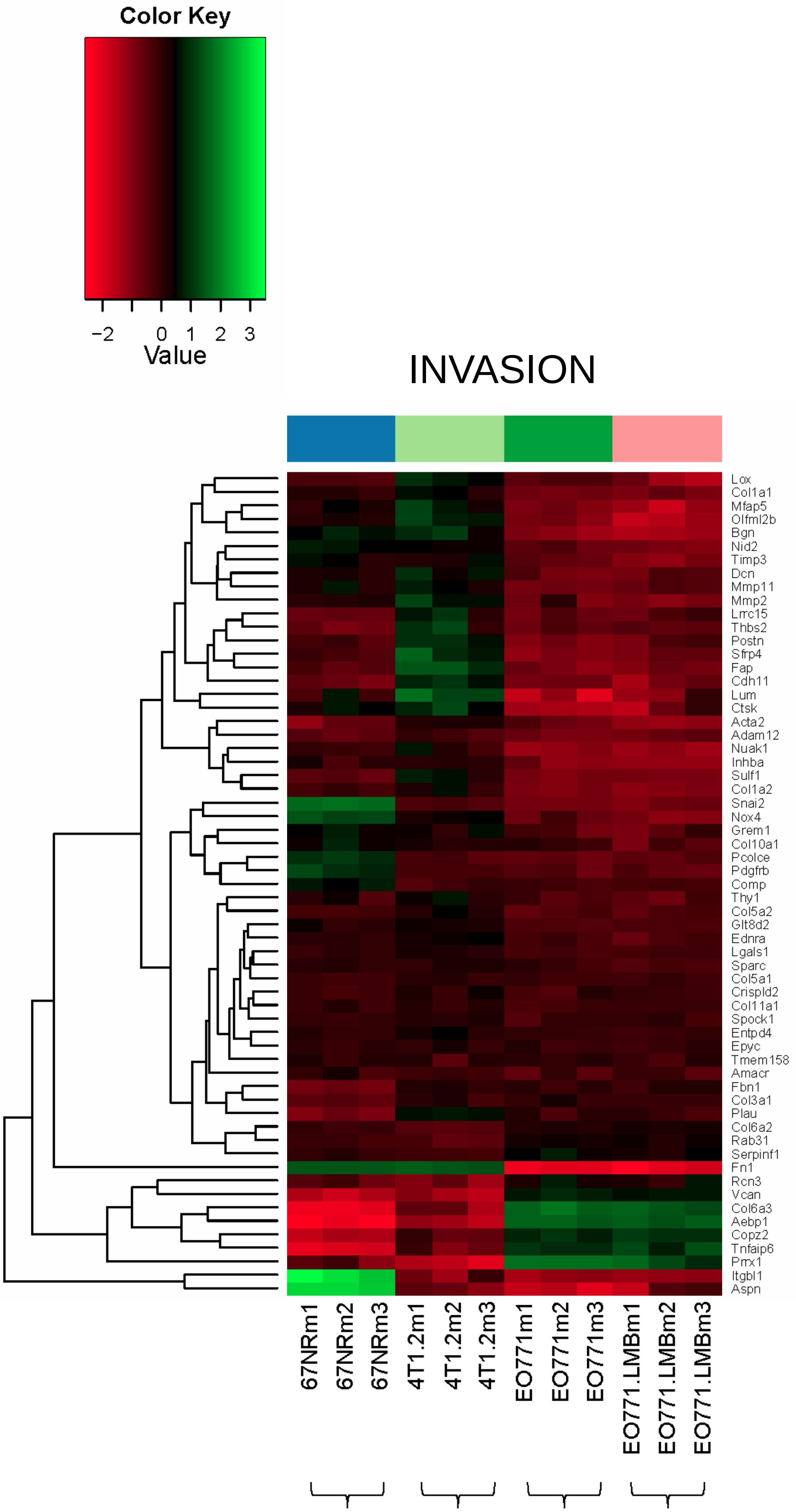

Supp. Figure 9F

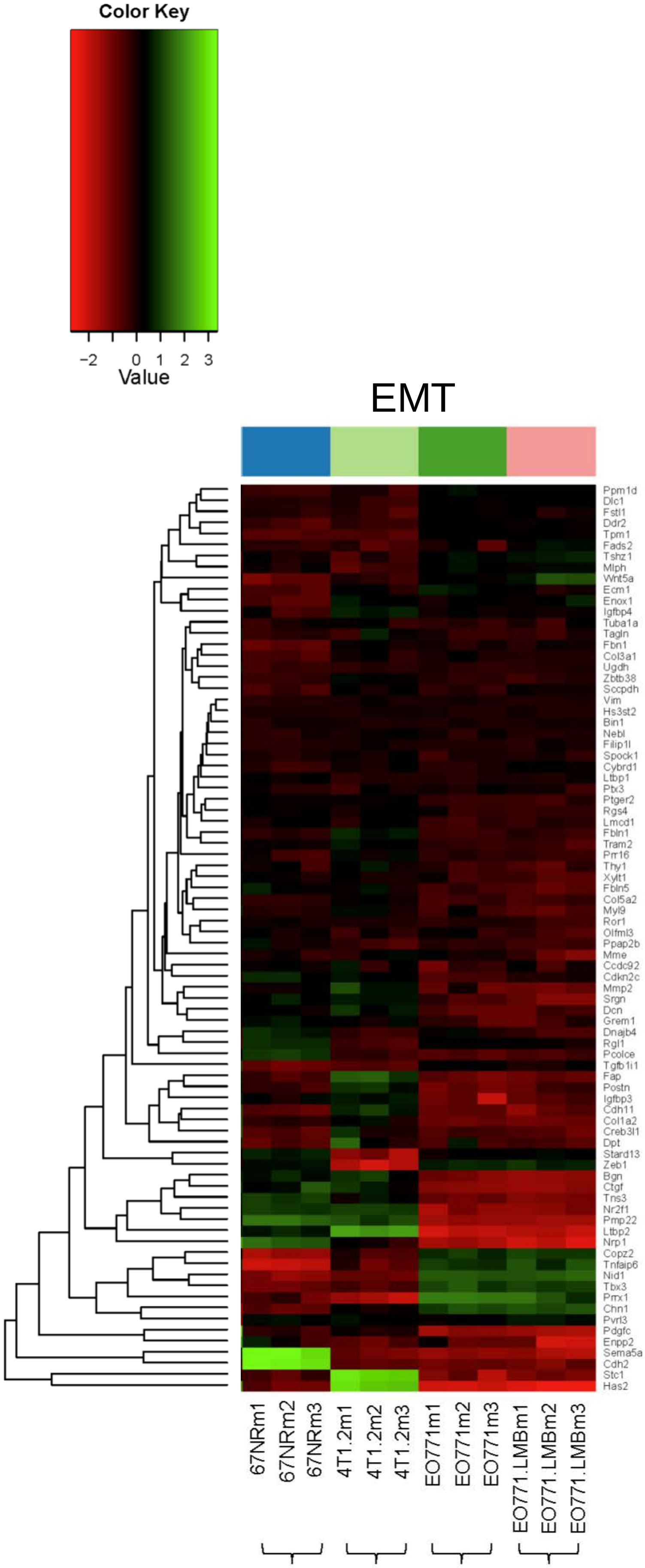

Supp. Figure 9G

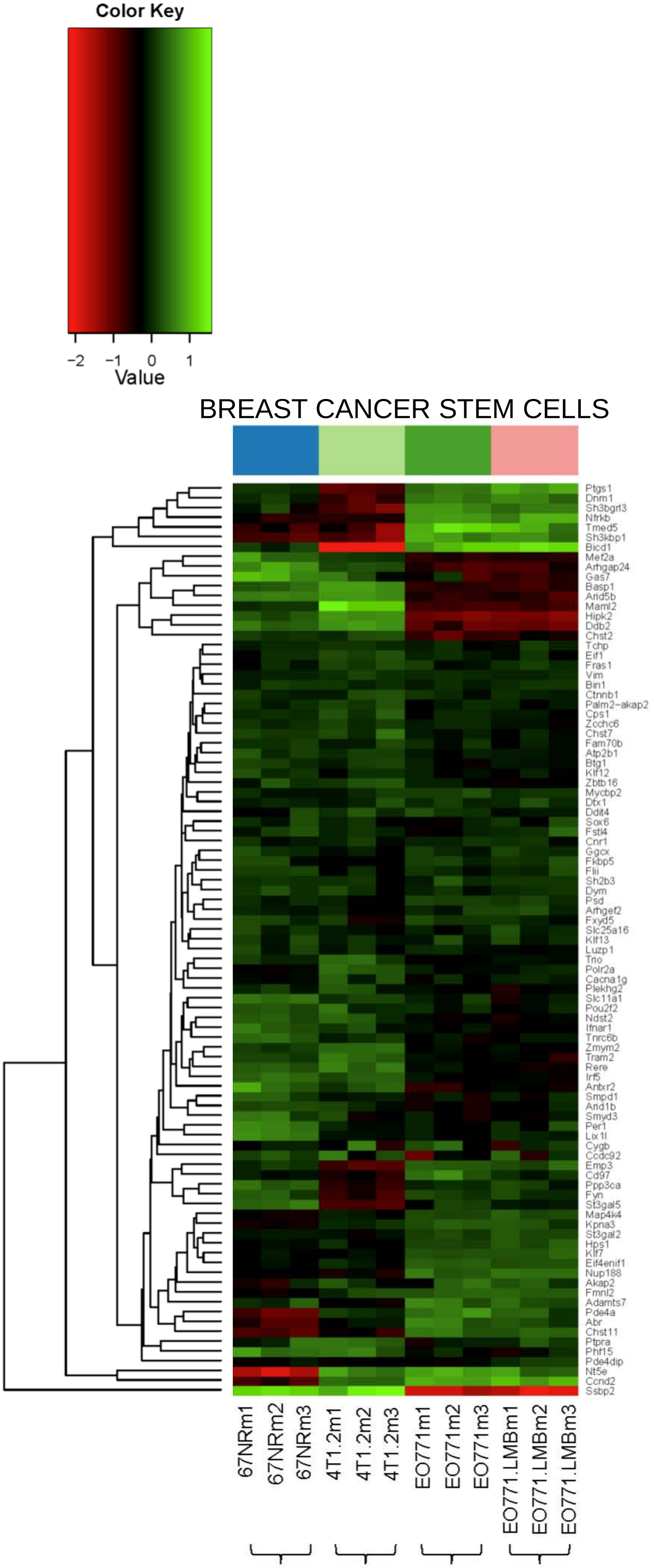

Supp. Figure 9H

Supplementary Figure 10

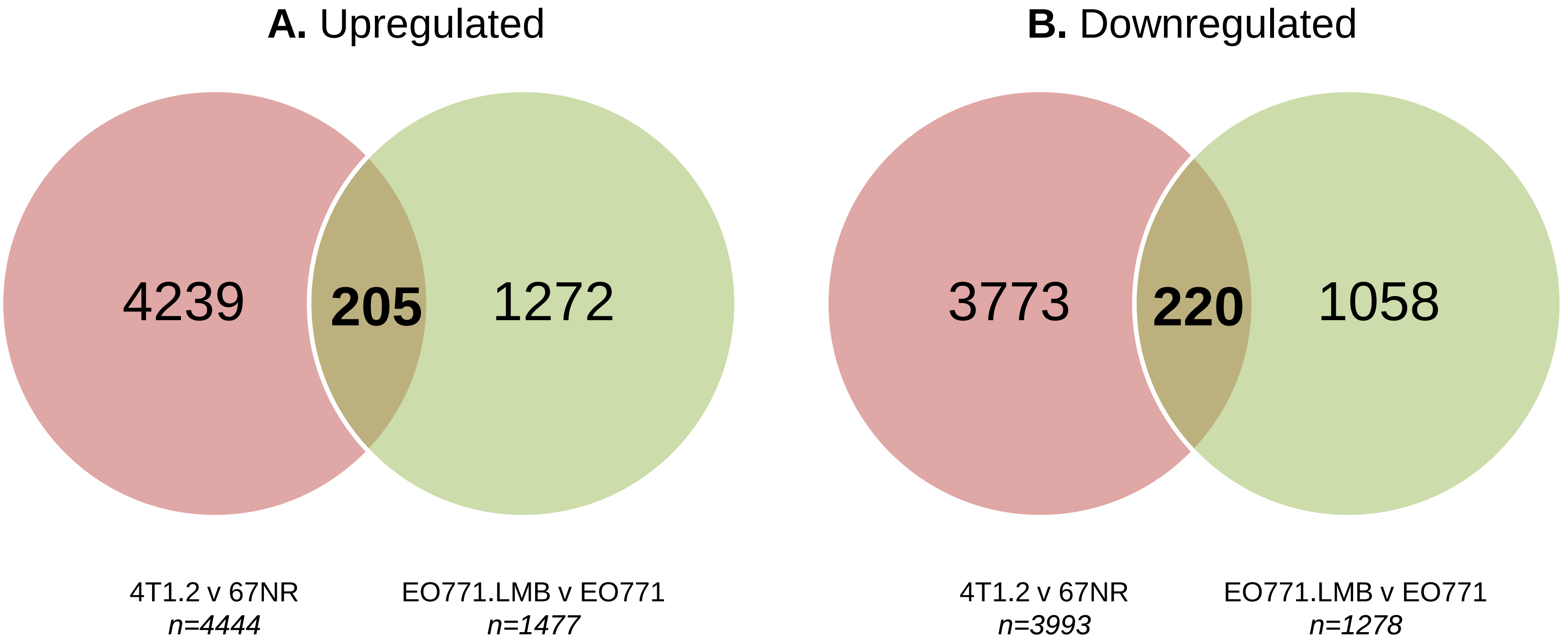

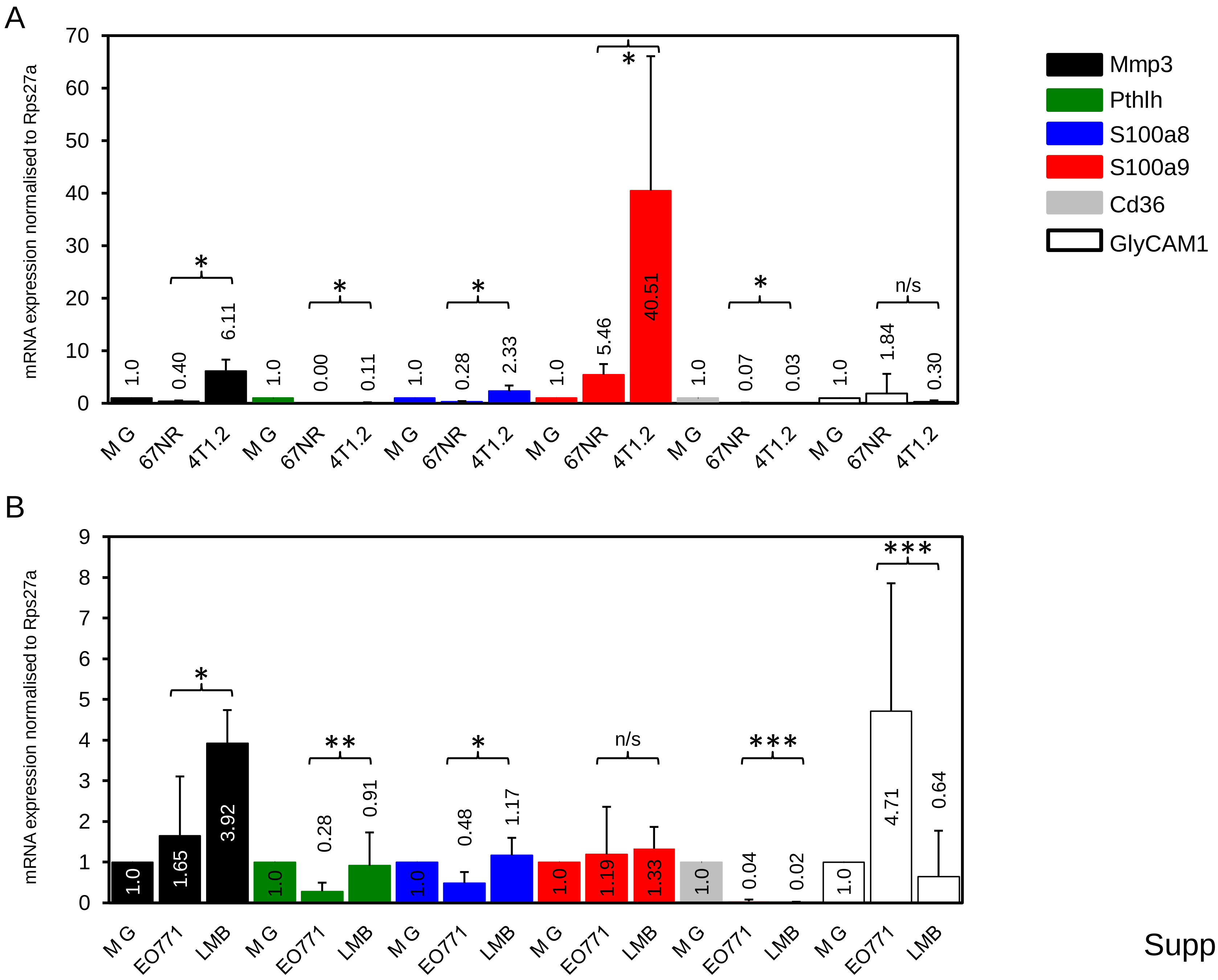

Supp. Figure 11

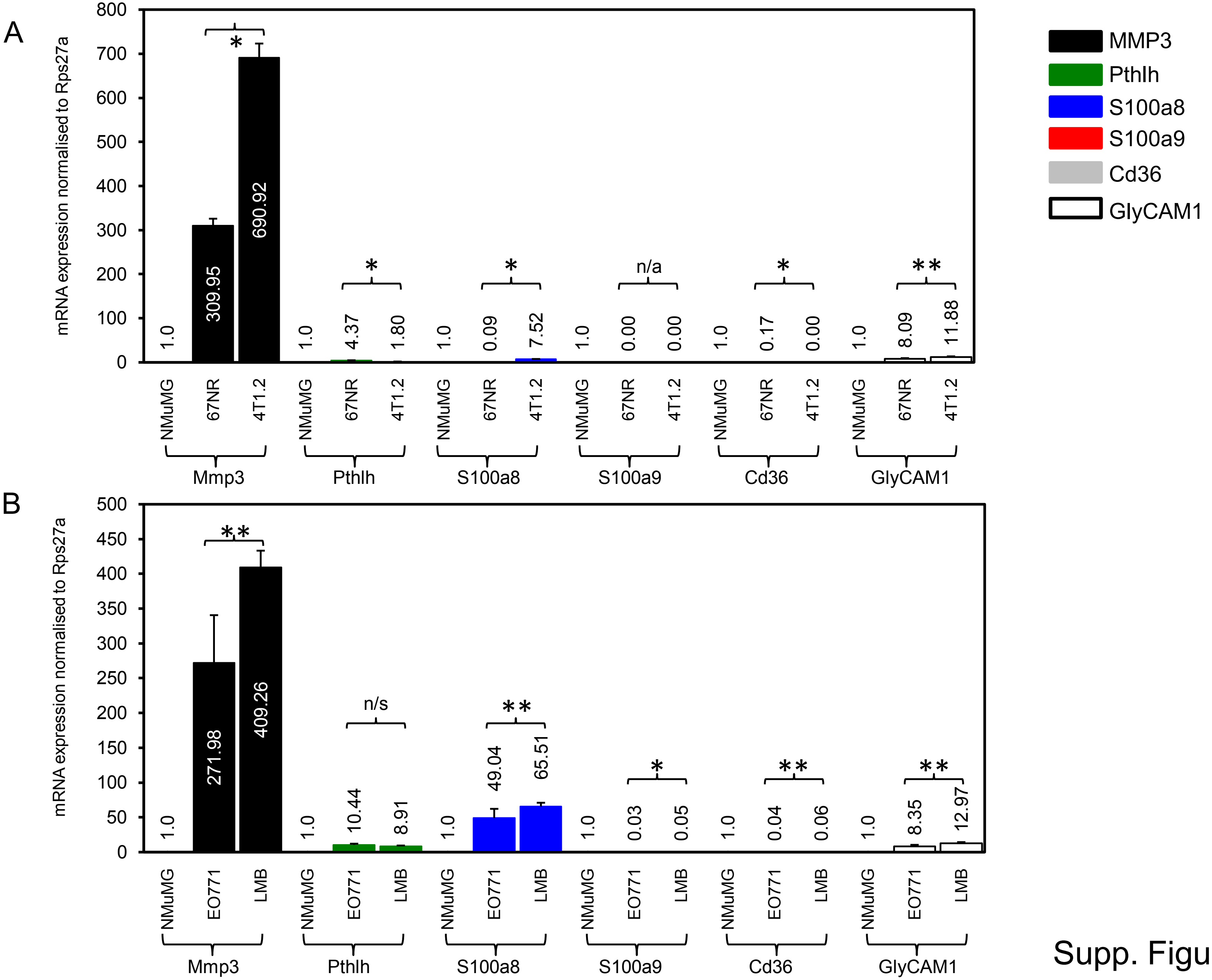

Supp. Figure 12

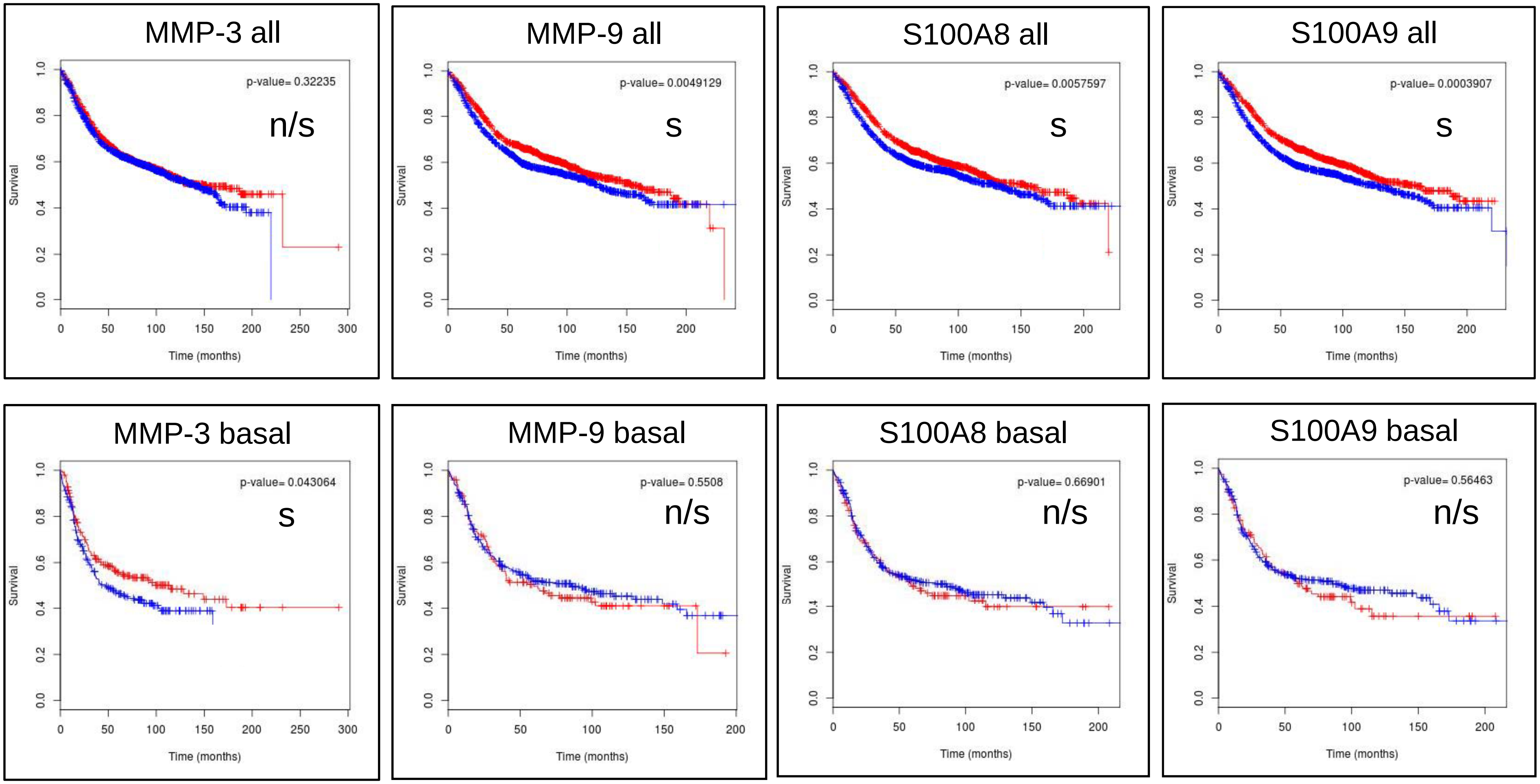

Supplementary Figure 13

Supplementary Table 1

Signature	67NR	4T1.2	EO771	EO771.LMB
BASAL sig-UP	4 (9%)	11 (23%)	5 (11%)	5 (11%)
BASAL sig-DOWN	11 (23%)	3 (6%)	11 (23%)	10 (21%)
BASAL insignificant	32 (68%)	33 (70%)	31 (66%)	32 (68%)
LUMINAL sig-UP	10 (18%)	**15 (27%)**	4 (7%)	8 (15%)
LUMINAL sig-DOWN	14 (25%)	3 (5%)	17 (31%)	15 (27%)
LUMINAL insignificant	31 (56%)	37 (67%)	34 (62%)	32 (58%)
PROLIFERATION sig-UP	6 (6%)	4 (4%)	**50 (48%)**	**57 (54%)**
PROLIFERATION sig-DOWN	61 (58%)	24 (23%)	5 (5%)	4 (4%)
PROLIFERATION insignificant	38 (36%)	77 (73%)	50 (48%)	44 (42%)
HYPOXIA sig-UP	3 (3%)	6 (5%)	**28 (25%)**	**29 (25%)**
HYPOXIA sig-DOWN	43 (38%)	17 (15%)	5 (4%)	2 (2%)
HYPOXIA insignificant	68 (60%)	91 (80%)	81 (71%)	83 (73%)
EMT sig-UP	22 (24%)	16 (17%)	16 (17%)	15 (17%)
EMT sig-DOWN	27 (29%)	13 (14%)	29 (31%)	28 (30%)
EMT insignificant	44 (47%)	64 (69%)	48 (52%)	50 (54%)
INTERFERON sig-UP	1 (4%)	3 (12%)	**9 (35%)**	**13 (50%)**
INTERFERON sig-DOWN	13 (50%)	3 (12%)	3 (12%)	1 (4%)
INTERFERON insignificant	12 (46%)	20 (77%)	14 (54%)	12 (46%)
CANCER STEM CELLS sig-UP	23 (23%)	20 (20%)	17 (17%)	20 (20%)
CANCER STEM CELLS sig-DOWN	15 (15%)	17 (17%)	18 (18%)	18 (18%)
CANCER STEM CELLS insignificant	62 (62%)	63 (63%)	65 (65%)	62 (62%)
INVASION sig-UP	13 (20%)	14 (22%)	8 (13%)	9 (14%)
INVASION sig-DOWN	17 (27%)	10 (16%)	30 (47%)	31 (48%)
INVASION insignificant	34 (53%)	40 (63%)	26 (41%)	24 (38%)
**SIGNIFICANT GENE EXPRESSION SIGNATURES**		**LUMINAL**	**PROLIFERATION HYPOXIA INTERFERON**	**PROLIFERATION HYPOXIA INTERFERON**

Supplementary Table 2: Commonly down-regulated genes [with unadjusted P value of 0.05 n= 220]

Transcript Cluster ID	Gene name	Gene Symbol	Ref Seq ID	Unadjusted *P* value (4T1.2 vs 67NR)	Adjusted *P* value (4T1.2 vs 67NR)	Ratio (4T1.2 vs 67NR)	Fold Change (4T1.2 vs 67NR)	Unadjusted *P* value (LMB vs EO771)	Adjusted *P* value (LMB vs EO771)	Ratio (LMB vs EO771)	Fold Change (LMB vs EO771)
10356271	NM_213615 // A530032D15Rik // RIKEN cDNA A530032D15Rik gene // 1 C5 // 381287 //	A530032D15Rik	NM_213615	0.0385619	0.1829850	0.9220660	-1.0845200	0.0107124	0.5584920	0.8553480	-1.1691100
10344333	---		---	0.0366596	0.1775120	0.8996910	-1.1114900	0.0470203	0.6381170	0.8025940	-1.2459600
10562663	NM_001081036 // EG668668 // predicted gene, EG668668 // 7 B4 // 668668 /// NM_00	EG668668	NM_001081036	0.0115267	0.0953788	0.8962650	-1.1157400	0.0251239	0.5966630	0.9380840	-1.0660000
10424126	NM_001037937 // Depdc6 // DEP domain containing 6 // 15 D1|15 6.7 cM // 97998 //	Depdc6	NM_001037937	0.0421835	0.1919230	0.8819190	-1.1338900	0.0273462	0.6015850	0.7166970	-1.3952900
10453887	NM_001146287 // Cables1 // CDK5 and Abl enzyme substrate 1 // 18 A2 // 63955 ///	Cables1	NM_001146287	0.0130114	0.1019690	0.8796160	-1.1368600	0.0149245	0.5813830	0.8855230	-1.1292800
10412921	NM_008695 // Nid2 // nidogen 2 // 14 A3 // 18074 /// ENSMUST00000022340 // Nid2	Nid2	NM_008695	0.0463027	0.2024550	0.8792130	-1.1373800	0.0491529	0.6427990	0.7854890	-1.2730900
10453423	ENSMUST00000095186 // ENSMUSG00000071036 // predicted gene, ENSMUSG00000071036 /	ENSMUSG00000071036	ENSMUST00000095186	0.0030392	0.0475628	0.8792000	-1.1374000	0.0247002	0.5966630	0.8873440	-1.1269600
10532310	`	4930522L14Rik	BC065392	0.0379159	0.1809340	0.8788750	-1.1378200	0.0358695	0.6185970	0.7748910	-1.2905000
10598474	NM_078484 // Slc35a2 // solute carrier family 35 (UDP-galactose transporter), me	Slc35a2	NM_078484	0.0242192	0.1412160	0.8744540	-1.1435700	0.0312777	0.6097130	0.8577940	-1.1657800
10451110	NM_008302 // Hsp90ab1 // heat shock protein 90 alpha (cytosolic), class B member	Hsp90ab1	NM_008302	0.0070422	0.0736881	0.8704940	-1.1487700	0.0389446	0.6222000	0.8869040	-1.1275200
10344147	---		---	0.0391451	0.1844600	0.8687690	-1.1510500	0.0453700	0.6350840	0.6549850	-1.5267500
10382795	XR_034731 // OTTMUSG00000003903 // predicted gene, OTTMUSG00000003903 // 11 E2 /	OTTMUSG00000003903	XR_034731	0.0443309	0.1980190	0.8530370	-1.1722800	0.0316434	0.6111430	0.8903750	-1.1231200
10542378	NM_019426 // Atf7ip // activating transcription factor 7 interacting protein //	Atf7ip	NM_019426	0.0178499	0.1205250	0.8477130	-1.1796500	0.0054561	0.5233400	0.8679100	-1.1521900
10362314	NM_008983 // Ptprk // protein tyrosine phosphatase, receptor type, K // 10 A4|10	Ptprk	NM_008983	0.0021997	0.0401273	0.8313830	-1.2028200	0.0158261	0.5813830	0.6462970	-1.5472800
10445239	ENSMUST00000071458 // EG546797 // predicted gene, EG546797 // 17 B3 // 546797	EG546797	ENSMUST00000071458	0.0034113	0.0504212	0.8240110	-1.2135800	0.0338228	0.6152010	0.9103630	-1.0984600
10604072	NM_026055 // Rpl39 // ribosomal protein L39 // X F3 // 67248 /// ENSMUST00000038	Rpl39	NM_026055	0.0047097	0.0592196	0.8158100	-1.2257800	0.0115066	0.5666630	0.8211190	-1.2178500
10564343	NM_009386 // Tjp1 // tight junction protein 1 // 7 C|7 28.5 cM // 21872 /// ENSM	Tjp1	NM_009386	0.0194309	0.1258900	0.8117430	-1.2319200	0.0303330	0.6077970	0.8500690	-1.1763700
10407192	NM_178746 // Slc38a9 // solute carrier family 38, member 9 // 13 D2.2 // 268706	Slc38a9	NM_178746	0.0220926	0.1347150	0.8110340	-1.2329900	0.0013727	0.4796000	0.7235930	-1.3819900
10554945	NM_028243 // Prcp // prolylcarboxypeptidase (angiotensinase C) // 7 E2 // 72461	Prcp	NM_028243	0.0244857	0.1421870	0.8072590	-1.2387600	0.0154323	0.5813830	0.7776160	-1.2859800
10471571	NM_177345 // Mapkap1 // mitogen-activated protein kinase associated protein 1 //	Mapkap1	NM_177345	0.0187471	0.1237270	0.8024420	-1.2462000	0.0154123	0.5813830	0.9599770	-1.0416900
10538082	NM_133764 // Atp6v0e2 // ATPase, H+ transporting, lysosomal V0 subunit E2 // 6 B	Atp6v0e2	NM_133764	0.0208515	0.1304130	0.7963670	-1.2557000	0.0067593	0.5233400	0.7662910	-1.3049900
10518333	---		---	0.0228839	0.1370960	0.7880130	-1.2690200	0.0202108	0.5901910	0.8320610	-1.2018300
10534303	NM_020044 // Lat2 // linker for activation of T cells family, member 2 // 5 G2 /	Lat2	NM_020044	0.0419174	0.1911520	0.7854660	-1.2731300	0.0380896	0.6199630	0.8154190	-1.2263600
10381708	NM_019679 // Fmnl1 // formin-like 1 // --- // 57778 /// NM_001077698 // Fmnl1 //	Fmnl1	NM_019679	0.0125882	0.1000640	0.7797010	-1.2825400	0.0368613	0.6187790	0.8988110	-1.1125800
10380534	NM_025287 // Spop // speckle-type POZ protein // 11 D|11 55.6 cM // 20747 /// EN	Spop	NM_025287	0.0310741	0.1623020	0.7787850	-1.2840500	0.0497049	0.6440630	0.8809310	-1.1351600
10473793	NM_008948 // Psmc3 // proteasome (prosome, macropain) 26S subunit, ATPase 3 // 2	Psmc3	NM_008948	0.0184391	0.1227070	0.7777730	-1.2857200	0.0180633	0.5851120	0.7596290	-1.3164300
10382360	BC048942 // D11Wsu47e // DNA segment, Chr 11, Wayne State University 47, express	D11Wsu47e	BC048942	0.0311325	0.1623810	0.7732250	-1.2932800	0.0396543	0.6224180	0.7944940	-1.2586600
10580033	NM_011925 // Cd97 // CD97 antigen // 8 C2|8 38.0 cM // 26364 /// ENSMUST00000075	Cd97	NM_011925	0.0184057	0.1226070	0.7726830	-1.2941900	0.0223883	0.5966630	0.6926700	-1.4436900
10530319	NM_001038999 // Atp8a1 // ATPase, aminophospholipid transporter (APLT), class I,	Atp8a1	NM_001038999	0.0258910	0.1466130	0.7710290	-1.2969700	0.0401914	0.6253770	0.7595220	-1.3166200
10355532	NM_027884 // Tns1 // tensin 1 // 1 C3|1 44.5 cM // 21961	Tns1	NM_027884	0.0055110	0.0639465	0.7705420	-1.2977900	0.0432096	0.6340740	0.8862400	-1.1283600
10583363	---		---	0.0199996	0.1277090	0.7692570	-1.2999600	0.0452221	0.6350840	0.9209560	-1.0858300
10447294	NM_011104 // Prkce // protein kinase C, epsilon // 17 E4 // 18754 /// ENSMUST000	Prkce	NM_011104	0.0457340	0.2011030	0.7639310	-1.3090200	0.0084374	0.5362340	0.6677030	-1.4976700
10574438	NM_009868 // Cdh5 // cadherin 5 // 8 D3|8 51.0 cM // 12562 /// ENSMUST0000003433	Cdh5	NM_009868	0.0189500	0.1243150	0.7599420	-1.3158900	0.0004973	0.3925740	0.3425640	-2.9191600
10339762	---		---	0.0454879	0.2005170	0.7554900	-1.3236400	0.0062709	0.5233400	0.6508700	-1.5364000
10535780	NM_010229 // Flt3 // FMS-like tyrosine kinase 3 // 5 G3|5 82.0 cM // 14255 /// E	Flt3	NM_010229	0.0051741	0.0619426	0.7539740	-1.3263100	0.0130601	0.5778020	0.7193200	-1.3902000
10422598	NM_009155 // Sepp1 // selenoprotein P, plasma, 1 // 15 A1|15 5.9 cM // 20363 ///	Sepp1	NM_009155	0.0069277	0.0730701	0.7523870	-1.3291000	0.0347851	0.6172700	0.5538770	-1.8054600
10494395	NM_013549 // Hist2h2aa1 // histone cluster 2, H2aa1 // 3 F1-F2|3 45.2 cM // 1526	Hist2h2aa1	NM_013549	0.0494360	0.2093550	0.7478320	-1.3372000	0.0375598	0.6190170	0.7326150	-1.3649700
10360028	NM_001077189 // Fcgr2b // Fc receptor, IgG, low affinity IIb // 1 H3|1 92.3 cM /	Fcgr2b	NM_001077189	0.0259917	0.1468790	0.7450400	-1.3422100	0.0136859	0.5778020	0.7727500	-1.2940800
10440849	ENSMUST00000023696 // Synj1 // synaptojanin 1 // 16 C3-C4 // 104015 /// AB093272	Synj1	ENSMUST00000023696	0.0012538	0.0301754	0.7438770	-1.3443100	0.0418079	0.6296050	0.8195190	-1.2202300
10394124	NM_178664 // B3gntl1 // UDP-GlcNAc:betaGal beta-1,3-N-acetylglucosaminyltransfer	B3gntl1	NM_178664	0.0247316	0.1431480	0.7434660	-1.3450500	0.0101745	0.5506320	0.8307680	-1.2037100
10347919	BC100303 // A530040E14Rik // RIKEN cDNA A530040E14 gene // 1 C5 // 621875 /// EN	A530040E14Rik	BC100303	0.0013001	0.0308285	0.7382190	-1.3546100	0.0293678	0.6053730	0.8122030	-1.2312200
10356291	BC100303 // A530040E14Rik // RIKEN cDNA A530040E14 gene // 1 C5 // 621875 /// EN	A530040E14Rik	BC100303	0.0013001	0.0308285	0.7382190	-1.3546100	0.0293676	0.6053730	0.8122020	-1.2312200
10357875	NM_007570 // Btg2 // B-cell translocation gene 2, anti-proliferative // 1 E4|1 7	Btg2	NM_007570	0.0223608	0.1356400	0.7369570	-1.3569300	0.0025844	0.4958410	0.6310650	-1.5846200
10441864	NM_010806 // Mllt4 // myeloid/lymphoid or mixed-lineage leukemia (trithorax homo	Mllt4	NM_010806	0.0043799	0.0574443	0.7348520	-1.3608200	0.0130930	0.5778020	0.8658310	-1.1549600
10582860	BC100303 // A530040E14Rik // RIKEN cDNA A530040E14 gene // 1 C5 // 621875 /// EN	A530040E14Rik	BC100303	0.0011007	0.0282764	0.7340520	-1.3623000	0.0266394	0.6007050	0.8020220	-1.2468500
10582877	BC100303 // A530040E14Rik // RIKEN cDNA A530040E14 gene // 1 C5 // 621875 /// EN	A530040E14Rik	BC100303	0.0011006	0.0282764	0.7340510	-1.3623000	0.0266396	0.6007050	0.8020210	-1.2468500
10371067	NM_021501 // Pias4 // protein inhibitor of activated STAT 4 // 10 C1 // 59004 //	Pias4	NM_021501	0.0072388	0.0747596	0.7332240	-1.3638400	0.0356758	0.6172700	0.7935990	-1.2600800
10530536	NM_001113460 // Tec // tec protein tyrosine kinase // 5 C3.2|5 41.0 cM // 21682	Tec	NM_001113460	0.0096519	0.0866334	0.7329720	-1.3643100	0.0166797	0.5851120	0.5957840	-1.6784600
10385118	NM_033374 // Dock2 // dedicator of cyto-kinesis 2 // 11 A5 // 94176 /// ENSMUST0	Dock2	NM_033374	0.0257950	0.1462070	0.7327970	-1.3646300	0.0013170	0.4796000	0.6982540	-1.4321400
10347921	ENSMUST00000093501 // A530040E14Rik // RIKEN cDNA A530040E14 gene // 1 C5 // 621	A530040E14Rik	ENSMUST00000093501	0.0035933	0.0517261	0.7314720	-1.3671100	0.0457408	0.6350840	0.6966180	-1.4355100
10368888	NM_019740 // Foxo3 // forkhead box O3 // 10 B2|10 30.0 cM // 56484 /// ENSMUST00	Foxo3	NM_019740	0.0071371	0.0742654	0.7304680	-1.3689900	0.0409829	0.6284950	0.8679640	-1.1521200
10481574	NM_175511 // Fam78a // family with sequence similarity 78, member A // 2 B // 24	Fam78a	NM_175511	0.0342932	0.1713690	0.7267940	-1.3759100	0.0298956	0.6071280	0.8566920	-1.1672800
10559446	NM_011095 // Lilrb3 // leukocyte immunoglobulin-like receptor, subfamily B (with	Lilrb3	NM_011095	0.0358460	0.1754120	0.7263800	-1.3766900	0.0097436	0.5455800	0.7409440	-1.3496300
10496813	NM_028836 // Ctbs // chitobiase, di-N-acetyl- // 3 H3 // 74245 /// NM_027617 //	Ctbs	NM_028836	0.0088522	0.0827903	0.7252240	-1.3788800	0.0273459	0.6015850	0.8723610	-1.1463100
10438232	NM_010048 // Dgcr2 // DiGeorge syndrome critical region gene 2 // 16 A-B1|16 10.	Dgcr2	NM_010048	0.0343138	0.1713810	0.7225640	-1.3839600	0.0223188	0.5966630	0.8971480	-1.1146400
10518346	NM_001085522 // OTTMUSG00000011097 // predicted gene, OTTMUSG00000011097 // 4 E1	OTTMUSG00000011097	NM_001085522	0.0325416	0.1666980	0.7138190	-1.4009100	0.0067474	0.5233400	0.7757470	-1.2890800
10492049	BC052702 // Fam48a // family with sequence similarity 48, member A // 3 D // 567	Fam48a	BC052702	0.0016694	0.0349768	0.7124660	-1.4035800	0.0332972	0.6144630	0.9296950	-1.0756200
10445338	NM_032003 // Enpp5 // ectonucleotide pyrophosphatase/phosphodiesterase 5 // 17 C	Enpp5	NM_032003	0.0004835	0.0187245	0.7119160	-1.4046600	0.0058616	0.5233400	0.5539980	-1.8050600
10460237	NM_019449 // Unc93b1 // unc-93 homolog B1 (C. elegans) // 19 A // 54445 /// ENSM	Unc93b1	NM_019449	0.0468877	0.2035530	0.7111490	-1.4061800	0.0135714	0.5778020	0.8425880	-1.1868200
10433219	NM_029090 // Nat15 // N-acetyltransferase 15 (GCN5-related, putative) // 16 A1 /	Nat15	NM_029090	0.0136886	0.1049280	0.7081840	-1.4120600	0.0485957	0.6420950	0.8856520	-1.1291100
10447361	NM_028639 // Ttc7 // tetratricopeptide repeat domain 7 // 17 E4 // 225049 /// EN	Ttc7	NM_028639	0.0432136	0.1948640	0.7055810	-1.4172700	0.0255963	0.5966630	0.8372610	-1.1943700
10489961	NM_010899 // Nfatc2 // nuclear factor of activated T-cells, cytoplasmic, calcine	Nfatc2	NM_010899	0.0231914	0.1381220	0.7051150	-1.4182100	0.0133102	0.5778020	0.8445700	-1.1840300
10508444	NM_025970 // Zbtb8os // zinc finger and BTB domain containing 8 opposite strand	Zbtb8os	NM_025970	0.0334006	0.1689560	0.7048850	-1.4186700	0.0000786	0.2539610	0.8581240	-1.1653300
10341831	---		---	0.0422601	0.1920990	0.6998640	-1.4288500	0.0202741	0.5901910	0.7027420	-1.4230000
10585767	NM_001122873 // Ubl7 // ubiquitin-like 7 (bone marrow stromal cell-derived) // 9	Ubl7	NM_001122873	0.0438234	0.1966410	0.6997040	-1.4291800	0.0365797	0.6187790	0.7291280	-1.3715000
10377982	NM_153103 // Kif1c // kinesin family member 1C // 11 B3 // 16562 /// ENSMUST0000	Kif1c	NM_153103	0.0144022	0.1076980	0.6975210	-1.4336500	0.0023784	0.4914630	0.8709610	-1.1481600
10491526	NM_172678 // Acad9 // acyl-Coenzyme A dehydrogenase family, member 9 // 3 B // 2	Acad9	NM_172678	0.0067993	0.0721223	0.6974760	-1.4337400	0.0193322	0.5890940	0.9235080	-1.0828300
10567702	NM_144529 // Arhgap17 // Rho GTPase activating protein 17 // 7 F3 // 70497 /// N	Arhgap17	NM_144529	0.0263957	0.1479160	0.6938520	-1.4412300	0.0194128	0.5890940	0.7645710	-1.3079200
10555389	NM_011671 // Ucp2 // uncoupling protein 2 (mitochondrial, proton carrier) // 7 E	Ucp2	NM_011671	0.0056014	0.0644497	0.6921150	-1.4448500	0.0468230	0.6378690	0.6461650	-1.5475900
10475487	NM_172980 // Slc28a2 // solute carrier family 28 (sodium-coupled nucleoside tran	Slc28a2	NM_172980	0.0419310	0.1911900	0.6902990	-1.4486500	0.0300458	0.6077060	0.8212680	-1.2176300
10344434	---		---	0.0403361	0.1872560	0.6901010	-1.4490600	0.0182991	0.5851120	0.5424830	-1.8433800
10524844	NM_001081308 // Taok3 // TAO kinase 3 // 5 F // 330177 /// ENSMUST00000111978 //	Taok3	NM_001081308	0.0473009	0.2046080	0.6898090	-1.4496800	0.0329557	0.6144630	0.8389520	-1.1919600
10384154	NM_178440 // Myo1g // myosin IG // --- // 246177 /// ENSMUST00000003459 // Myo1g	Myo1g	NM_178440	0.0070951	0.0740308	0.6887320	-1.4519400	0.0457277	0.6350840	0.6886110	-1.4522000
10524079	ENSMUST00000089906 // 2310001H12Rik // RIKEN cDNA 2310001H12 gene // 5 F // 6950	2310001H12Rik	ENSMUST00000089906	0.0003476	0.0170714	0.6875850	-1.4543600	0.0184125	0.5851120	0.8357480	-1.1965300
10421581	NM_001033439 // Lrch1 // leucine-rich repeats and calponin homology (CH) domain	Lrch1	NM_001033439	0.0157978	0.1130350	0.6853440	-1.4591200	0.0266675	0.6008800	0.6666560	-1.5000200
10498367	NM_028808 // P2ry13 // purinergic receptor P2Y, G-protein coupled 13 // 3 D // 7	P2ry13	NM_028808	0.0253769	0.1450410	0.6829030	-1.4643400	0.0309669	0.6089260	0.7675460	-1.3028500
10391963	NM_008740 // Nsf // N-ethylmaleimide sensitive fusion protein // 11 E1|11 63.0 c	Nsf	NM_008740	0.0000409	0.0060137	0.6820270	-1.4662200	0.0229261	0.5966630	0.8569250	-1.1669600
10424662	NM_028216 // Psca // prostate stem cell antigen // 15 D3 // 72373 /// ENSMUST000	Psca	NM_028216	0.0223370	0.1355780	0.6800130	-1.4705600	0.0100783	0.5494770	0.8140640	-1.2284000
10379389	NM_172133 // Adap2 // ArfGAP with dual PH domains 2 // 11 B5|11 47.24 cM // 2169	Adap2	NM_172133	0.0010549	0.0277830	0.6719540	-1.4882000	0.0181742	0.5851120	0.7264310	-1.3765900
10504424	NM_016678 // Reck // reversion-inducing-cysteine-rich protein with kazal motifs	Reck	NM_016678	0.0031433	0.0484473	0.6707930	-1.4907700	0.0281347	0.6015850	0.8261120	-1.2104900
10514956	NM_011327 // Scp2 // sterol carrier protein 2, liver // 4 C7|4 52.0 cM // 20280	Scp2	NM_011327	0.0055636	0.0643104	0.6691780	-1.4943700	0.0146229	0.5813830	0.7273400	-1.3748700
10475890	NM_008587 // Mertk // c-mer proto-oncogene tyrosine kinase // 2 F1 // 17289 ///	Mertk	NM_008587	0.0026904	0.0445745	0.6678330	-1.4973800	0.0221137	0.5966630	0.7508240	-1.3318700
10437698	NM_029582 // Txndc11 // thioredoxin domain containing 11 // 16 B1 // 106200 ///	Txndc11	NM_029582	0.0064464	0.0700942	0.6657490	-1.5020700	0.0178127	0.5851120	0.8107210	-1.2334700
10527732	ENSMUST00000087204 // Fry // furry homolog (Drosophila) // 5 G3 // 320365 /// EN	Fry	ENSMUST00000087204	0.0325770	0.1667830	0.6643500	-1.5052300	0.0082367	0.5362340	0.6942130	-1.4404800
10555438	NM_199012 // Fchsd2 // FCH and double SH3 domains 2 // 7 E3 // 207278 /// NM_001	Fchsd2	NM_199012	0.0205518	0.1293350	0.6631900	-1.5078600	0.0204982	0.5913910	0.7648340	-1.3074700
10514221	NM_007408 // Adfp // adipose differentiation related protein // 4 C4|4 38.9 cM /	Adfp	NM_007408	0.0108796	0.0923994	0.6617650	-1.5111100	0.0230295	0.5966630	0.6375270	-1.5685600
10598839	NM_133669 // Rp2h // retinitis pigmentosa 2 homolog (human) // X A2 // 19889 ///	Rp2h	NM_133669	0.0309154	0.1619040	0.6603290	-1.5144000	0.0008683	0.4607930	0.6988820	-1.4308600
10427918	BC052328 // Fam105a // family with sequence similarity 105, member A // 15 B1 //	Fam105a	BC052328	0.0436440	0.1961220	0.6594560	-1.5164000	0.0046315	0.5233400	0.7360270	-1.3586400
10461487	NM_201351 // Cybasc3 // cytochrome b, ascorbate dependent 3 // 19 A // 225912 //	Cybasc3	NM_201351	0.0421609	0.1918440	0.6575360	-1.5208300	0.0263820	0.5980440	0.7616500	-1.3129400
10506488	NM_080555 // Ppap2b // phosphatidic acid phosphatase type 2B // 4 C6|4 52.7 cM /	Ppap2b	NM_080555	0.0492155	0.2088940	0.6527500	-1.5319800	0.0115016	0.5666630	0.8332250	-1.2001600
10405033	NM_001012324 // Ecm2 // extracellular matrix protein 2, female organ and adipocy	Ecm2	NM_001012324	0.0078901	0.0782323	0.6500400	-1.5383700	0.0046997	0.5233400	0.7854180	-1.2732100
10375167	NM_001025382 // EG574403 // predicted gene, EG574403 // 11 A4|11 // 574403 /// E	EG574403	NM_001025382	0.0023237	0.0414976	0.6484330	-1.5421800	0.0339515	0.6166390	0.8581910	-1.1652400
10495186	ENSMUST00000070085 // AI504432 // expressed sequence AI504432 // 3 F2.3 // 22969	AI504432	ENSMUST00000070085	0.0154953	0.1115440	0.6469080	-1.5458100	0.0296600	0.6066280	0.6913370	-1.4464700
10459785	NM_201354 // Gm672 // gene model 672, (NCBI) // 18 E3 // 269037 /// ENSMUST00000	Gm672	NM_201354	0.0036046	0.0517844	0.6443900	-1.5518600	0.0247684	0.5966630	0.8075370	-1.2383300
10494390	NM_013549 // Hist2h2aa1 // histone cluster 2, H2aa1 // 3 F1-F2|3 45.2 cM // 1526	Hist2h2aa1	NM_013549	0.0084123	0.0805943	0.6429900	-1.5552300	0.0181278	0.5851120	0.6214470	-1.6091500
10558921	NM_025802 // Pnpla2 // patatin-like phospholipase domain containing 2 // 7 F5 //	Pnpla2	NM_025802	0.0158343	0.1131470	0.6423800	-1.5567100	0.0212305	0.5966630	0.8719860	-1.1468100
10348558	NM_016717 // Scly // selenocysteine lyase // 1 D // 50880 /// ENSMUST00000027532	Scly	NM_016717	0.0148997	0.1097750	0.6410130	-1.5600300	0.0323176	0.6144630	0.8205820	-1.2186500
10506154	NM_001081264 // Alg6 // asparagine-linked glycosylation 6 homolog (yeast, alpha-	Alg6	NM_001081264	0.0144453	0.1078120	0.6407720	-1.5606200	0.0309963	0.6089260	0.8415650	-1.1882600
10586118	NM_138304 // Calml4 // calmodulin-like 4 // 9 B // 75600 /// NM_001102468 // Cal	Calml4	NM_138304	0.0423549	0.1922600	0.6396550	-1.5633400	0.0334439	0.6144630	0.8689530	-1.1508100
10570573	NM_026792 // Agpat5 // 1-acylglycerol-3-phosphate O-acyltransferase 5 (lysophosp	Agpat5	NM_026792	0.0117296	0.0960362	0.6391390	-1.5646000	0.0254544	0.5966630	0.7298900	-1.3700700
10551696	NM_145149 // Rasgrp4 // RAS guanyl releasing protein 4 // 7 B1|7 20.0 cM // 2330	Rasgrp4	NM_145149	0.0109901	0.0928846	0.6355440	-1.5734500	0.0389381	0.6222000	0.8031200	-1.2451400
10561031	NM_010719 // Lipe // lipase, hormone sensitive // 7 A3|7 5.5 cM // 16890 /// NM_	Lipe	NM_010719	0.0066047	0.0709907	0.6315560	-1.5833900	0.0070989	0.5313900	0.7778690	-1.2855600
10522051	NM_008453 // Klf3 // Kruppel-like factor 3 (basic) // 5 C3.1 // 16599 /// DQ9818	Klf3	NM_008453	0.0311068	0.1623110	0.6258890	-1.5977300	0.0287907	0.6032510	0.8469380	-1.1807200
10345580	NM_030266 // Inpp4a // inositol polyphosphate-4-phosphatase, type I // 1 B|1 19.	Inpp4a	NM_030266	0.0219642	0.1343070	0.6236870	-1.6033700	0.0076508	0.5362340	0.8602090	-1.1625100
10384233	NM_001083587 // Tns3 // tensin 3 // 11 A1 // 319939 /// ENSMUST00000020695 // Tn	Tns3	NM_001083587	0.0157147	0.1125610	0.6208840	-1.6106100	0.0382641	0.6199630	0.8126860	-1.2304900
10367822	NM_026405 // Rab32 // RAB32, member RAS oncogene family // 10 A1 // 67844 /// EN	Rab32	NM_026405	0.0304147	0.1604100	0.6206480	-1.6112200	0.0292306	0.6048710	0.7402640	-1.3508700
10446425	---		---	0.0111108	0.0933494	0.6203820	-1.6119100	0.0455425	0.6350840	0.6923290	-1.4444000
10527638	NM_009663 // Alox5ap // arachidonate 5-lipoxygenase activating protein // --- //	Alox5ap	NM_009663	0.0107352	0.0918209	0.6199320	-1.6130800	0.0366622	0.6187790	0.7357150	-1.3592200
10579052	ENSMUST00000095282 // ENSMUSG00000057924 // predicted gene, ENSMUSG00000057924 /	ENSMUSG00000057924	ENSMUST00000095282	0.0145075	0.1079990	0.6145080	-1.6273200	0.0315532	0.6102110	0.9055620	-1.1042900
10580219	NM_007591 // Calr // calreticulin // 8 C3|8 37.0 cM // 12317 /// ENSMUST00000003	Calr	NM_007591	0.0000244	0.0049109	0.6071630	-1.6470000	0.0360135	0.6185970	0.7884420	-1.2683200
10570144	NM_001113518 // Arhgef7 // Rho guanine nucleotide exchange factor (GEF7) // 8 A1	Arhgef7	NM_001113518	0.0341009	0.1708220	0.6025700	-1.6595600	0.0157898	0.5813830	0.8619220	-1.1602000
10607868	NM_133212 // Tlr8 // toll-like receptor 8 // X F5 // 170744 /// ENSMUST000000490	Tlr8	NM_133212	0.0022872	0.0411146	0.5976770	-1.6731400	0.0246352	0.5966630	0.7523260	-1.3292100
10362097	BC132022 // Raet1b // retinoic acid early transcript beta // 10 A3 // 19369 ///	Raet1b	BC132022	0.0101199	0.0889113	0.5975400	-1.6735300	0.0340016	0.6166390	0.5656850	-1.7677700
10501802	NM_178936 // Tmem56 // transmembrane protein 56 // 3 G3 // 99887 /// ENSMUST0000	Tmem56	NM_178936	0.0188240	0.1238570	0.5958240	-1.6783500	0.0061714	0.5233400	0.3899250	-2.5646000
10408693	NM_028784 // F13a1 // coagulation factor XIII, A1 subunit // 13 A3.3 // 74145 //	F13a1	NM_028784	0.0140870	0.1065170	0.5956500	-1.6788400	0.0176188	0.5851120	0.7052260	-1.4179800
10422822	NM_013584 // Lifr // leukemia inhibitory factor receptor // 15 A1|15 4.6 cM // 1	Lifr	NM_013584	0.0199067	0.1275090	0.5895610	-1.6961800	0.0213885	0.5966630	0.7014430	-1.4256300
10543306	NM_173007 // Tspan12 // tetraspanin 12 // 6 A3.1 // 269831 /// ENSMUST0000003167	Tspan12	NM_173007	0.0408152	0.1884440	0.5891890	-1.6972500	0.0253375	0.5966630	0.6690620	-1.4946300
10366886	NM_146011 // Arhgap9 // Rho GTPase activating protein 9 // 10 D3 // 216445 /// E	Arhgap9	NM_146011	0.0136986	0.1049280	0.5883240	-1.6997400	0.0181526	0.5851120	0.8880150	-1.1261100
10363231	NM_020561 // Smpdl3a // sphingomyelin phosphodiesterase, acid-like 3A // 10 B4 /	Smpdl3a	NM_020561	0.0008888	0.0251222	0.5856830	-1.7074100	0.0173841	0.5851120	0.8277600	-1.2080800
10510178	NM_001037926 // OTTMUSG00000010173 // predicted gene, OTTMUSG00000010173 // 4 E1	OTTMUSG00000010173	NM_001037926	0.0016312	0.0344202	0.5831070	-1.7149500	0.0148065	0.5813830	0.6643770	-1.5051700
10379535	NM_021443 // Ccl8 // chemokine (C-C motif) ligand 8 // 11 C|11 47.0 cM // 20307	Ccl8	NM_021443	0.0417931	0.1908450	0.5820400	-1.7180900	0.0435864	0.6349300	0.5532920	-1.8073600
10538993	NM_001081110 // Cd8a // CD8 antigen, alpha chain // 6 C|6 30.5 cM // 12525 /// N	Cd8a	NM_001081110	0.0044520	0.0578744	0.5816800	-1.7191600	0.0443178	0.6350840	0.5050480	-1.9800100
10601980	NM_175541 // Mum1l1 // melanoma associated antigen (mutated) 1-like 1 // X F1 //	Mum1l1	NM_175541	0.0185865	0.1232270	0.5775210	-1.7315400	0.0224381	0.5966630	0.8266380	-1.2097200
10522335	NR_003966 // Atp10d // ATPase, class V, type 10D // 5 C3.2 // 231287 /// NM_1533	Atp10d	NR_003966	0.0171081	0.1181610	0.5772550	-1.7323400	0.0129040	0.5778020	0.7905270	-1.2649800
10489723	NM_027230 // Zmynd8 // zinc finger, MYND-type containing 8 // 2 H3 // 228880 ///	Zmynd8	NM_027230	0.0021445	0.0395598	0.5770160	-1.7330500	0.0032496	0.5090030	0.9095590	-1.0994300
10354649	ENSMUST00000094950 // Pgap1 // post-GPI attachment to proteins 1 // 1 C1.1 // 24	Pgap1	ENSMUST00000094950	0.0015229	0.0333853	0.5769500	-1.7332500	0.0342863	0.6172700	0.6273610	-1.5939800
10559248	NM_020286 // Tspan32 // tetraspanin 32 // 7 F5|7 69.0 cM // 27027 /// NM_0011280	Tspan32	NM_020286	0.0165992	0.1161130	0.5766440	-1.7341700	0.0076066	0.5357610	0.7546320	-1.3251500
10471945	NM_015753 // Zeb2 // zinc finger E-box binding homeobox 2 // 2 C1 // 24136 /// E	Zeb2	NM_015753	0.0307572	0.1613560	0.5758730	-1.7364900	0.0294654	0.6062910	0.8780060	-1.1389400
10600235	NM_007435 // Abcd1 // ATP-binding cassette, sub-family D (ALD), member 1 // X B|	Abcd1	NM_007435	0.0097986	0.0874273	0.5749210	-1.7393700	0.0111259	0.5628710	0.8568140	-1.1671100
10436841	NM_008349 // Il10rb // interleukin 10 receptor, beta // 16 C3.3|16 63.11 cM // 1	Il10rb	NM_008349	0.0128505	0.1012890	0.5737980	-1.7427700	0.0127289	0.5772810	0.8419840	-1.1876700
10410656	NM_009817 // Cast // calpastatin // 13 C1 // 12380 /// ENSMUST00000065629 // Cas	Cast	NM_009817	0.0050259	0.0614993	0.5673090	-1.7627100	0.0046632	0.5233400	0.7636830	-1.3094400
10498210	NM_030595 // Nbea // neurobeachin // 3 C|3 28.9 cM // 26422 /// ENSMUST000000293	Nbea	NM_030595	0.0007225	0.0227535	0.5635890	-1.7743400	0.0331499	0.6144630	0.7893650	-1.2668400
10598848	NM_199317 // Phf16 // PHD finger protein 16 // X A1.3 // 382207 /// ENSMUST00000	Phf16	NM_199317	0.0028970	0.0463988	0.5632000	-1.7755700	0.0230060	0.5966630	0.6669620	-1.4993300
10421526	NM_009029 // Rb1 // retinoblastoma 1 // 14 D3|14 41.0 cM // 19645 /// ENSMUST000	Rb1	NM_009029	0.0000772	0.0080275	0.5550900	-1.8015100	0.0425054	0.6296050	0.8323030	-1.2014900
10490854	NM_001037926 // OTTMUSG00000010173 // predicted gene, OTTMUSG00000010173 // 4 E1	OTTMUSG00000010173	NM_001037926	0.0007140	0.0225852	0.5548950	-1.8021400	0.0176877	0.5851120	0.6479410	-1.5433500
10510167	NM_001037926 // OTTMUSG00000010173 // predicted gene, OTTMUSG00000010173 // 4 E1	OTTMUSG00000010173	NM_001037926	0.0004407	0.0186104	0.5512880	-1.8139300	0.0262920	0.5977220	0.6420970	-1.5574000
10453797	NM_001083628 // AK220484 // cDNA sequence AK220484 // 18 A1 // 381157 /// ENSMUS	AK220484	NM_001083628	0.0024003	0.0422415	0.5495800	-1.8195700	0.0243277	0.5966630	0.8907090	-1.1227000
10594404	NM_016769 // Smad3 // MAD homolog 3 (Drosophila) // --- // 17127 /// ENSMUST0000	Smad3	NM_016769	0.0214134	0.1325560	0.5493430	-1.8203600	0.0133598	0.5778020	0.6967110	-1.4353200
10517169	NM_080559 // Sh3bgrl3 // SH3 domain binding glutamic acid-rich protein-like 3 //	Sh3bgrl3	NM_080559	0.0196745	0.1267400	0.5430200	-1.8415500	0.0355683	0.6172700	0.8714480	-1.1475100
10356932	BC023951 // D1Ertd622e // DNA segment, Chr 1, ERATO Doi 622, expressed // 1 D|1	D1Ertd622e	BC023951	0.0223271	0.1355410	0.5412620	-1.8475400	0.0302428	0.6077970	0.4227360	-2.3655400
10371220	NM_010304 // Gna15 // guanine nucleotide binding protein, alpha 15 // 10 C1|10 4	Gna15	NM_010304	0.0324647	0.1665210	0.5332250	-1.8753800	0.0215894	0.5966630	0.7079090	-1.4126100
10482500	NM_028810 // Rnd3 // Rho family GTPase 3 // 2 C1.1 // 74194 /// ENSMUST000000172	Rnd3	NM_028810	0.0029082	0.0464697	0.5332080	-1.8754400	0.0183048	0.5851120	0.9231100	-1.0832900
10345037	NM_028829 // Paqr8 // progestin and adipoQ receptor family member VIII // 1 A5 /	Paqr8	NM_028829	0.0110828	0.0932684	0.5245360	-1.9064500	0.0211439	0.5966630	0.8848890	-1.1300900
10577144	NM_001024504 // Dcun1d2 // DCN1, defective in cullin neddylation 1, domain conta	Dcun1d2	NM_001024504	0.0010286	0.0273534	0.5177100	-1.9315800	0.0441962	0.6350840	0.7555970	-1.3234600
10343924	---		---	0.0156896	0.1124800	0.5122450	-1.9521900	0.0095426	0.5399900	0.4923560	-2.0310500
10540207	ENSMUST00000049189 // Adamts9 // a disintegrin-like and metallopeptidase (reprol	Adamts9	ENSMUST00000049189	0.0079633	0.0786513	0.5082740	-1.9674400	0.0084358	0.5362340	0.7936030	-1.2600800
10439744	NM_032465 // Cd96 // CD96 antigen // 16 B5 // 84544 /// ENSMUST00000023336 // Cd	Cd96	NM_032465	0.0272281	0.1507280	0.5033860	-1.9865500	0.0245940	0.5966630	0.7404390	-1.3505500
10521667	NM_009763 // Bst1 // bone marrow stromal cell antigen 1 // 5 B3|5 25.0 cM // 121	Bst1	NM_009763	0.0115913	0.0956522	0.5030700	-1.9877900	0.0439287	0.6349300	0.6097400	-1.6400400
10411611	NM_010870 // Naip5 // NLR family, apoptosis inhibitory protein 5 // 13 D1|13 55.	Naip5	NM_010870	0.0089180	0.0830291	0.5007130	-1.9971500	0.0451177	0.6350840	0.7444140	-1.3433400
10594241	ENSMUST00000063858 // ENSMUSG00000052143 // predicted gene, ENSMUSG00000052143 /	ENSMUSG00000052143	ENSMUST00000063858	0.0053171	0.0627575	0.5001460	-1.9994200	0.0199740	0.5898630	0.7980140	-1.2531100
10502823	NM_025926 // Dnajb4 // DnaJ (Hsp40) homolog, subfamily B, member 4 // 3 H3 // 67	Dnajb4	NM_025926	0.0005028	0.0189972	0.4941420	-2.0237100	0.0271851	0.6015850	0.6404280	-1.5614600
10567825	NM_010689 // Lat // linker for activation of T cells // 7 F3 // 16797 /// ENSMUS	Lat	NM_010689	0.0399476	0.1862310	0.4900570	-2.0405800	0.0273650	0.6015850	0.6271730	-1.5944600
10409857	NM_178098 // 4930486L24Rik // RIKEN cDNA 4930486L24 gene // 13 B2 // 214639 ///	4930486L24Rik	NM_178098	0.0078423	0.0780512	0.4877280	-2.0503200	0.0082809	0.5362340	0.8263810	-1.2101000

10340934	---		---	0.0051460	0.0618542	0.4854810	-2.0598100	0.0138596	0.5778020	0.5865590	-1.7048600
10466659	NM_010266 // Gda // guanine deaminase // 19 B|19 15.5 cM // 14544 /// ENSMUST000	Gda	NM_010266	0.0073030	0.0750915	0.4815320	-2.0767000	0.0040650	0.5169010	0.6224430	-1.6065700
10604175	NM_172930 // Fam70a // family with sequence similarity 70, member A // X A3.3 //	Fam70a	NM_172930	0.0013256	0.0310698	0.4739150	-2.1100800	0.0416374	0.6296050	0.7557280	-1.3232300
10579958	NM_008357 // Il15 // interleukin 15 // 8 C2|8 38.0 cM // 16168 /// ENSMUST000000	Il15	NM_008357	0.0002855	0.0155197	0.4535470	-2.2048400	0.0248056	0.5966630	0.7694350	-1.2996500
10502050	ENSMUST00000029662 // Alpk1 // alpha-kinase 1 // 3 H1 // 71481 /// AK173191 // A	Alpk1	ENSMUST00000029662	0.0006765	0.0219542	0.4530160	-2.2074300	0.0324168	0.6144630	0.6566140	-1.5229600
10435948	NM_026439 // Ccdc80 // coiled-coil domain containing 80 // 16 B5 // 67896 /// EN	Ccdc80	NM_026439	0.0413847	0.1896720	0.4448850	-2.2477700	0.0210825	0.5966630	0.6081840	-1.6442400
10542214	NM_010654 // Klrd1 // killer cell lectin-like receptor, subfamily D, member 1 //	Klrd1	NM_010654	0.0042543	0.0567751	0.4330540	-2.3091800	0.0177135	0.5851120	0.7198590	-1.3891600
10343830	---		---	0.0329630	0.1677690	0.4318800	-2.3154600	0.0425358	0.6296050	0.7057030	-1.4170300
10561008	NM_001039185 // Ceacam1 // carcinoembryonic antigen-related cell adhesion molecu	Ceacam1	NM_001039185	0.0031759	0.0485275	0.4267050	-2.3435400	0.0062151	0.5233400	0.4475960	-2.2341600
10561854	NM_025548 // Tbcb // tubulin folding cofactor B // 7 B1 // 66411 /// ENSMUST0000	Tbcb	NM_025548	0.0004191	0.0185342	0.4264730	-2.3448200	0.0404069	0.6254130	0.9028630	-1.1075900
10528207	NM_001159557 // Cd36 // CD36 antigen // 5 A3|5 2.0 cM // 12491 /// NM_001159558	Cd36	NM_001159557	0.0229149	0.1371890	0.4237860	-2.3596800	0.0358474	0.6185970	0.3520580	-2.8404400
10546454	NM_175314 // Adamts9 // a disintegrin-like and metallopeptidase (reprolysin type	Adamts9	NM_175314	0.0054494	0.0636816	0.4107420	-2.4346200	0.0126129	0.5737140	0.6288850	-1.5901200
10498576	NM_016753 // Lxn // latexin // 3 E1|3 31.6 cM // 17035 /// ENSMUST00000058981 //	Lxn	NM_016753	0.0017708	0.0360116	0.4099140	-2.4395300	0.0152350	0.5813830	0.8632320	-1.1584400
10344240	---		---	0.0264687	0.1482370	0.4055010	-2.4660800	0.0023276	0.4852670	0.6815520	-1.4672400
10420308	NM_013542 // Gzmb // granzyme B // 14 D3|14 20.5 cM // 14939 /// ENSMUST00000015	Gzmb	NM_013542	0.0122919	0.0988317	0.4026290	-2.4836800	0.0171895	0.5851120	0.6286250	-1.5907700
10576639	NM_008737 // Nrp1 // neuropilin 1 // 8 E|8 73.0 cM // 18186 /// ENSMUST000000269	Nrp1	NM_008737	0.0051442	0.0618542	0.3873000	-2.5819800	0.0160799	0.5821440	0.6949470	-1.4389600
10443598	NM_013811 // Dnahc8 // dynein, axonemal, heavy chain 8 // 17 A3.3 // 13417 /// E	Dnahc8	NM_013811	0.0234520	0.1389050	0.3812420	-2.6230000	0.0368101	0.6187790	0.6845600	-1.4607900
10587383	NM_153098 // Cd109 // CD109 antigen // 9 E1 // 235505 /// ENSMUST00000093812 //	Cd109	NM_153098	0.0010659	0.0278679	0.3731840	-2.6796500	0.0089466	0.5362340	0.5625810	-1.7775200
10546432	NM_175314 // Adamts9 // a disintegrin-like and metallopeptidase (reprolysin type	Adamts9	NM_175314	0.0183299	0.1223250	0.3684670	-2.7139500	0.0311069	0.6092170	0.6742470	-1.4831400
10559837	NR_003555 // Vmn2r29 // vomeronasal 2, receptor 29 // 7 A1 // 76229 /// NM_00111	Vmn2r29	NR_003555	0.0007751	0.0234139	0.3633210	-2.7523900	0.0414646	0.6296050	0.7927130	-1.2614900
10479950	NM_001110231 // Cugbp2 // CUG triplet repeat, RNA binding protein 2 // 2 A2-A3 /	Cugbp2	NM_001110231	0.0002242	0.0136448	0.3602010	-2.7762300	0.0015216	0.4796000	0.5127150	-1.9504000
10341019	---		---	0.0005591	0.0199281	0.3489110	-2.8660600	0.0223856	0.5966630	0.7569270	-1.3211300
10518147	NM_010329 // Pdpn // podoplanin // 4 E1 // 14726 /// ENSMUST00000030317 // Pdpn	Pdpn	NM_010329	0.0026244	0.0440359	0.3352070	-2.9832300	0.0004419	0.3925740	0.3710270	-2.6952200
10560744	NM_203489 // V1rd15 // vomeronasal 1 receptor, D15 // 7 A3 // 209824 /// NM_2068	V1rd15	NM_203489	0.0000629	0.0072456	0.3263050	-3.0646100	0.0266428	0.6007050	0.8378820	-1.1934900
10408850	NM_001111324 // Nedd9 // neural precursor cell expressed, developmentally down-r	Nedd9	NM_001111324	0.0001160	0.0097488	0.3090340	-3.2358900	0.0286212	0.6032510	0.6934800	-1.4420000
10510241	NM_001014397 // OTTMUSG00000010673 // predicted gene, OTTMUSG00000010673 // 4 E1	OTTMUSG00000010673	NM_001014397	0.0002927	0.0156983	0.2944470	-3.3961900	0.0046965	0.5233400	0.6813740	-1.4676200
10392522	NM_153145 // Abca8a // ATP-binding cassette, sub-family A (ABC1), member 8a // 1	Abca8a	NM_153145	0.0001879	0.0123509	0.2901660	-3.4463100	0.0262584	0.5977220	0.9176460	-1.0897400
10458663	NM_009468 // Dpysl3 // dihydropyrimidinase-like 3 // 18 B3 // 22240 /// NM_00113	Dpysl3	NM_009468	0.0006472	0.0213673	0.2426510	-4.1211500	0.0000672	0.2539610	0.8719480	-1.1468600
10551393	NM_007434 // Akt2 // thymoma viral proto-oncogene 2 // 7 B1|7 6.5 cM // 11652 //	Akt2	NM_007434	0.0003332	0.0167552	0.2356870	-4.2429200	0.0315035	0.6102110	0.5560880	-1.7982800
10603953	NM_001040669 // 382277 // predicted gene, 382277 // X A2 // 382277 /// NM_001109	382277	NM_001040669	0.0003094	0.0162041	0.2192580	-4.5608300	0.0021973	0.4852520	0.6598100	-1.5155900
10448836	NM_001001183 // Tmem204 // transmembrane protein 204 // 17 A3.3 // 407831 /// EN	Tmem204	NM_001001183	0.0050382	0.0614993	0.2191390	-4.5633200	0.0153889	0.5813830	0.4556420	-2.1947100
10483439	NM_001081088 // Lrp2 // low density lipoprotein receptor-related protein 2 // 2	Lrp2	NM_001081088	0.0000407	0.0060121	0.2180280	-4.5865600	0.0056018	0.5233400	0.6807870	-1.4688900
10462237	NM_011416 // Smarca2 // SWI/SNF related, matrix associated, actin dependent regu	Smarca2	NM_011416	0.0000802	0.0080914	0.2147100	-4.6574500	0.0075649	0.5357610	0.6615210	-1.5116700
10414245	NM_029131 // 4930503E14Rik // RIKEN cDNA 4930503E14 gene // 14 C1 // 74954 /// N	4930503E14Rik	NM_029131	0.0000028	0.0018498	0.2143800	-4.6646100	0.0217319	0.5966630	0.7200830	-1.3887300
10578324	NM_001005863 // Mtus1 // mitochondrial tumor suppressor 1 // 8 A4 // 102103 ///	Mtus1	NM_001005863	0.0000071	0.0028875	0.2115390	-4.7272600	0.0330706	0.6144630	0.8716370	-1.1472700
10550782	NM_030736 // V1rd14 // vomeronasal 1 receptor, D14 // 7 A3 // 81011 /// NM_00112	V1rd14	NM_030736	0.0000154	0.0038803	0.2084330	-4.7977000	0.0258384	0.5967500	0.8093630	-1.2355400
10565193	NM_013886 // Hdgfrp3 // hepatoma-derived growth factor, related protein 3 // 7 D	Hdgfrp3	NM_013886	0.0000059	0.0026675	0.1907490	-5.2424800	0.0209629	0.5966630	0.8010520	-1.2483600
10583870	NM_028472 // Bmper // BMP-binding endothelial regulator // 9 A4 // 73230 /// ENS	Bmper	NM_028472	0.0002251	0.0136569	0.1725750	-5.7945700	0.0313465	0.6097130	0.7520000	-1.3297900
10566934	NM_053247 // Lyve1 // lymphatic vessel endothelial hyaluronan receptor 1 // 7 F2	Lyve1	NM_053247	0.0029938	0.0471840	0.1618480	-6.1786400	0.0498667	0.6445140	0.5216240	-1.9170900
10521759	NM_178804 // Slit2 // slit homolog 2 (Drosophila) // 5 B3 // 20563 /// ENSMUST00	Slit2	NM_178804	0.0002125	0.0132844	0.1170620	-8.5424800	0.0151897	0.5813830	0.6645500	-1.5047800
10420295	NM_010374 // Gzmf // granzyme F // 14 C3|14 20.5 cM // 14943 /// ENSMUST00000022	Gzmf	NM_010374	0.0249082	0.1435250	0.1097960	-9.1078300	0.0461622	0.6354770	0.2504570	-3.9927000
10357870	NM_054077 // Prelp // proline arginine-rich end leucine-rich repeat // 1 E4|1 74	Prelp	NM_054077	0.0000234	0.0048500	0.0892324	-11.2067000	0.0029540	0.5001020	0.6930920	-1.4428100
10440534	NM_011782 // Adamts5 // a disintegrin-like and metallopeptidase (reprolysin type	Adamts5	NM_011782	0.0002570	0.0148111	0.0795736	-12.5670000	0.0213894	0.5966630	0.5146010	-1.9432500
10603962	AK157371 // ENSMUSG00000073266 // predicted gene, ENSMUSG00000073266 // --- // 1	ENSMUSG00000073266	AK157371	0.0000029	0.0018498	0.0641312	-15.5930000	0.0245412	0.5966630	0.8081650	-1.2373700
10603973	ENSMUST00000101657 // RP23-98M14.2 // novel protein similar to Xlr-related, meio	RP23-98M14.2	ENSMUST00000101657	0.0000029	0.0018498	0.0641312	-15.5930000	0.0245412	0.5966630	0.8081650	-1.2373700
10603984	AK157371 // ENSMUSG00000073266 // predicted gene, ENSMUSG00000073266 // --- // 1	ENSMUSG00000073266	AK157371	0.0000029	0.0018498	0.0641312	-15.5930000	0.0245412	0.5966630	0.8081650	-1.2373700
10603995	AK157371 // ENSMUSG00000073266 // predicted gene, ENSMUSG00000073266 // --- // 1	ENSMUSG00000073266	AK157371	0.0000029	0.0018498	0.0641312	-15.5930000	0.0245412	0.5966630	0.8081650	-1.2373700
10604006	ENSMUST00000101657 // RP23-98M14.2 // novel protein similar to Xlr-related, meio	RP23-98M14.2	ENSMUST00000101657	0.0000029	0.0018498	0.0641312	-15.5930000	0.0245412	0.5966630	0.8081650	-1.2373700
10604017	AK157371 // ENSMUSG00000073266 // predicted gene, ENSMUSG00000073266 // --- // 1	ENSMUSG00000073266	AK157371	0.0000029	0.0018498	0.0641312	-15.5930000	0.0245412	0.5966630	0.8081650	-1.2373700
10604021	AK157371 // ENSMUSG00000073266 // predicted gene, ENSMUSG00000073266 // --- // 1	ENSMUSG00000073266	AK157371	0.0000029	0.0018498	0.0641312	-15.5930000	0.0245412	0.5966630	0.8081650	-1.2373700
10599058	AK157371 // ENSMUSG00000073266 // predicted gene, ENSMUSG00000073266 // --- // 1	ENSMUSG00000073266	AK157371	0.0000029	0.0018498	0.0641311	-15.5930000	0.0245412	0.5966630	0.8081650	-1.2373700
10599060	AK157371 // ENSMUSG00000073266 // predicted gene, ENSMUSG00000073266 // --- // 1	ENSMUSG00000073266	AK157371	0.0000029	0.0018498	0.0641311	-15.5930000	0.0245412	0.5966630	0.8081650	-1.2373700
10599062	ENSMUST00000101657 // RP23-98M14.2 // novel protein similar to Xlr-related, meio	RP23-98M14.2	ENSMUST00000101657	0.0000029	0.0018498	0.0641311	-15.5930000	0.0245412	0.5966630	0.8081650	-1.2373700
10599073	ENSMUST00000101657 // RP23-98M14.2 // novel protein similar to Xlr-related, meio	RP23-98M14.2	ENSMUST00000101657	0.0000029	0.0018498	0.0641311	-15.5930000	0.0245412	0.5966630	0.8081650	-1.2373700
10599094	ENSMUST00000101657 // RP23-98M14.2 // novel protein similar to Xlr-related, meio	RP23-98M14.2	ENSMUST00000101657	0.0000029	0.0018498	0.0641311	-15.5930000	0.0245412	0.5966630	0.8081650	-1.2373700
10599105	AK157371 // ENSMUSG00000073266 // predicted gene, ENSMUSG00000073266 // --- // 1	ENSMUSG00000073266	AK157371	0.0000029	0.0018498	0.0641311	-15.5930000	0.0245412	0.5966630	0.8081650	-1.2373700
10496276	NM_028946 // Nhedc1 // Na+/H+ exchanger domain containing 1 // 3 G3 // 74446 ///	Nhedc1	NM_028946	0.0000085	0.0030958	0.0626492	-15.9619000	0.0470207	0.6381170	0.7161730	-1.3963100
10599064	NM_001109970 // RP23-110D11.1 // Xlr-related, meiosis regulated Xmr // X A3.1 //	RP23-110D11.1	NM_001109970	0.0000435	0.0061110	0.0568523	-17.5894000	0.0364612	0.6187790	0.7871180	-1.2704600
10599075	NM_001109970 // RP23-110D11.1 // Xlr-related, meiosis regulated Xmr // X A3.1 //	RP23-110D11.1	NM_001109970	0.0000316	0.0054732	0.0565378	-17.6873000	0.0335189	0.6144630	0.8050080	-1.2422200
10599107	NM_001109970 // RP23-110D11.1 // Xlr-related, meiosis regulated Xmr // X A3.1 //	RP23-110D11.1	NM_001109970	0.0000316	0.0054732	0.0565378	-17.6873000	0.0335189	0.6144630	0.8050080	-1.2422200
10603964	NM_001109970 // RP23-110D11.1 // Xlr-related, meiosis regulated Xmr // X A3.1 //	RP23-110D11.1	NM_001109970	0.0000316	0.0054732	0.0565378	-17.6873000	0.0335190	0.6144630	0.8050080	-1.2422200
10603997	NM_001109970 // RP23-110D11.1 // Xlr-related, meiosis regulated Xmr // X A3.1 //	RP23-110D11.1	NM_001109970	0.0000316	0.0054732	0.0565378	-17.6873000	0.0335190	0.6144630	0.8050080	-1.2422200
10604008	NM_001109970 // RP23-110D11.1 // Xlr-related, meiosis regulated Xmr // X A3.1 //	RP23-110D11.1	NM_001109970	0.0000316	0.0054732	0.0565378	-17.6873000	0.0335190	0.6144630	0.8050080	-1.2422200
10599096	NM_001109970 // RP23-110D11.1 // Xlr-related, meiosis regulated Xmr // X A3.1 //	RP23-110D11.1	NM_001109970	0.0000343	0.0057263	0.0497548	-20.0986000	0.0447258	0.6350840	0.8079570	-1.2376900
10603975	NM_001109970 // RP23-110D11.1 // Xlr-related, meiosis regulated Xmr // X A3.1 //	RP23-110D11.1	NM_001109970	0.0000343	0.0057263	0.0497548	-20.0986000	0.0447257	0.6350840	0.8079570	-1.2376900
10603986	NM_001109970 // RP23-110D11.1 // Xlr-related, meiosis regulated Xmr // X A3.1 //	RP23-110D11.1	NM_001109970	0.0000231	0.0048383	0.0462691	-21.6127000	0.0390473	0.6222000	0.8057210	-1.2411300
10604023	NM_001109970 // RP23-110D11.1 // Xlr-related, meiosis regulated Xmr // X A3.1 //	RP23-110D11.1	NM_001109970	0.0000231	0.0048383	0.0462691	-21.6127000	0.0390473	0.6222000	0.8057210	-1.2411300

Supplementary Table 3: Commonly up-regulated genes [with unadjusted P value of 0.05 n= 205]

Transcript Cluster ID	Gene name	Gene Symbol	Ref Seq ID	Unadjusted *P* value (4T1.2 vs 67NR)	Adjusted *P* value (4T1.2 vs 67NR)	Ratio (4T1.2 vs 67NR)	Fold Change (4T1.2 vs 67NR)	Unadjusted *P* value (LMB vs EO771)	Adjusted *P* value (LMB vs EO771)	Ratio (LMB vs EO771)	Fold Change (LMB vs EO771)
10583071	NM_010809 // Mmp3 // matrix metallopeptidase 3 // 9 A1|9 1.0 cM // 17392 /// ENS	Mmp3	NM_010809	0.0000855	0.0083795	12.1794000	12.1794000	0.0052774	0.5233400	2.1330000	2.1330000
10456545	NM_201362 // Ccdc68 // coiled-coil domain containing 68 // 18 E2 // 381175 /// E	Ccdc68	NM_201362	0.0000888	0.0084006	9.3553700	9.3553700	0.0346355	0.6172700	1.6183200	1.6183200
10423654	NM_054049 // Osr2 // odd-skipped related 2 (Drosophila) // 15 B3.1|15 14.0 cM //	Osr2	NM_054049	0.0008199	0.0243034	8.2308500	8.2308500	0.0176107	0.5851120	1.5929900	1.5929900
10436519	NM_019413 // Robo1 // roundabout homolog 1 (Drosophila) // 16 C3.1 // 19876 ///	Robo1	NM_019413	0.0003404	0.0169262	5.6509900	5.6509900	0.0355921	0.6172700	1.4414900	1.4414900
10483381	NM_016866 // Stk39 // serine/threonine kinase 39, STE20/SPS1 homolog (yeast) //	Stk39	NM_016866	0.0000076	0.0030097	5.2389200	5.2389200	0.0005079	0.3925740	1.1521000	1.1521000
10592336	NM_011449 // Spa17 // sperm autoantigenic protein 17 // 9 B // 20686 /// ENSMUST	Spa17	NM_011449	0.0002304	0.0138606	4.4707800	4.4707800	0.0426096	0.6296870	1.2811500	1.2811500
10584047	NM_013906 // Adamts8 // a disintegrin-like and metallopeptidase (reprolysin type	Adamts8	NM_013906	0.0029231	0.0465858	4.2063300	4.2063300	0.0124250	0.5737140	1.2717400	1.2717400
10493831	NM_013650 // S100a8 // S100 calcium binding protein A8 (calgranulin A) // 3 F1-F	S100a8	NM_013650	0.0062405	0.0688022	4.0832000	4.0832000	0.0125022	0.5737140	1.8282800	1.8282800
10590892	NM_026665 // Cep57 // centrosomal protein 57 // 9 A2 // 74360 /// ENSMUST0000003	Cep57	NM_026665	0.0000027	0.0018498	3.7371200	3.7371200	0.0087698	0.5362340	1.1048500	1.1048500
10608637	---		---	*0.0012699*	*0.0304058*	*3.3842300*	*3.3842300*	*0.0242561*	*0.5966630*	*1.8695900*	*1.8695900*
10363173	NM_010288 // Gja1 // gap junction protein, alpha 1 // 10 B4|10 29.0 cM // 14609	Gja1	NM_010288	0.0004747	0.0186104	3.1443600	3.1443600	0.0002859	0.3777900	1.4341200	1.4341200
10360920	NM_009367 // Tgfb2 // transforming growth factor, beta 2 // 1 H5|1 101.5 cM // 2	Tgfb2	NM_009367	0.0009236	0.0256727	3.1031900	3.1031900	0.0123323	0.5737140	1.1764800	1.1764800
10358668	NM_001024720 // Hmcn1 // hemicentin 1 // 1 G1 // 545370 /// ENSMUST00000074783 /	Hmcn1	NM_001024720	0.0100491	0.0887058	3.0641600	3.0641600	0.0166779	0.5851120	1.6174200	1.6174200
10459496	NM_178793 // Ccbe1 // collagen and calcium binding EGF domains 1 // 18 E1 // 320	Ccbe1	NM_178793	0.0005365	0.0195353	2.7812700	2.7812700	0.0093967	0.5362880	1.7493000	1.7493000
10343865	---		---	0.0190435	0.1246060	2.5975000	2.5975000	0.0224536	0.5966630	1.3772400	1.3772400
10549200	NM_011444 // Sox5 // SRY-box containing gene 5 // 6 G3|6 69.5 cM // 20678 /// NM	Sox5	NM_011444	0.0000975	0.0088428	2.5861500	2.5861500	0.0001570	0.3629660	1.4777300	1.4777300
10549497	BC106181 // 3010003L21Rik // RIKEN cDNA 3010003L21 gene // 6 G3 // 109163 /// NM	3010003L21Rik	BC106181	0.0017123	0.0353768	2.5289500	2.5289500	0.0187037	0.5877510	1.1088100	1.1088100
10377588	NM_009235 // Sox15 // SRY-box containing gene 15 // 11 B3|11 39.0 cM // 20670 //	Sox15	NM_009235	0.0000176	0.0042468	2.4591800	2.4591800	0.0248239	0.5966630	1.0847100	1.0847100
10523511	NM_008926 // Prkg2 // protein kinase, cGMP-dependent, type II // 5 E3|5 53.0 cM	Prkg2	NM_008926	0.0003375	0.0168321	2.3045300	2.3045300	0.0199575	0.5898630	2.1634200	2.1634200
10430400	NM_130859 // Card10 // caspase recruitment domain family, member 10 // 15 E1 //	Card10	NM_130859	0.0000160	0.0039896	2.1518500	2.1518500	0.0381616	0.6199630	1.1291200	1.1291200
10424467	NM_001081409 // Phf20l1 // PHD finger protein 20-like 1 // 15 D2 // 239510 /// E	Phf20l1	NM_001081409	0.0003869	0.0181232	2.1509100	2.1509100	0.0125323	0.5737140	1.1350600	1.1350600
10529957	ENSMUST00000030971 // Gpr125 // G protein-coupled receptor 125 // 5 B3 // 70693	Gpr125	ENSMUST00000030971	0.0019936	0.0381067	2.0653600	2.0653600	0.0370998	0.6187790	1.3426100	1.3426100
10479887	NM_021305 // Sec61a2 // Sec61, alpha subunit 2 (S. cerevisiae) // 2 A1 // 57743	Sec61a2	NM_021305	0.0034939	0.0509763	2.0327400	2.0327400	0.0202584	0.5901910	1.1491200	1.1491200
10354432	NM_010863 // Myo1b // myosin IB // 1 C1.1|1 24.8 cM // 17912 /// ENSMUST00000046	Myo1b	NM_010863	0.0008345	0.0243952	2.0297800	2.0297800	0.0166058	0.5851120	1.7285200	1.7285200
10546510	NM_008377 // Lrig1 // leucine-rich repeats and immunoglobulin-like domains 1 //	Lrig1	NM_008377	0.0018829	0.0371294	2.0129200	2.0129200	0.0400407	0.6244240	1.2354700	1.2354700
10347491	NM_009526 // Wnt6 // wingless-related MMTV integration site 6 // 1 C3 // 22420 /	Wnt6	NM_009526	0.0064517	0.0701307	2.0091300	2.0091300	0.0158026	0.5813830	1.1159600	1.1159600
10423599	NM_016762 // Matn2 // matrilin 2 // 15 B3.3 // 17181 /// ENSMUST00000022947 // M	Matn2	NM_016762	0.0012038	0.0297754	1.9949500	1.9949500	0.0116334	0.5687440	1.7567500	1.7567500
10341729	---		---	0.0176924	0.1200290	1.9506200	1.9506200	0.0188073	0.5877510	1.4459600	1.4459600
10430447	NM_138581 // 1700088E04Rik // RIKEN cDNA 1700088E04 gene // 15 E1|15 46.6 cM //	1700088E04Rik	NM_138581	0.0012444	0.0301602	1.9220200	1.9220200	0.0440364	0.6349300	1.1736200	1.1736200
10553403	NM_001146049 // Htatip2 // HIV-1 tat interactive protein 2, homolog (human) // 7	Htatip2	NM_001146049	0.0004063	0.0183113	1.9111100	1.9111100	0.0273066	0.6015850	1.2018300	1.2018300
10469035	NM_175400 // Sephs1 // selenophosphate synthetase 1 // 2 A1 // 109079 /// ENSMUS	Sephs1	NM_175400	0.0022553	0.0408017	1.8600500	1.8600500	0.0249186	0.5966630	1.1641400	1.1641400
10373452	NM_001033302 // Gm129 // gene model 129, (NCBI) // 3 F2.1 // 229599 /// BC132471	Gm129	NM_001033302	0.0025465	0.0433432	1.8549400	1.8549400	0.0301400	0.6077060	1.3995800	1.3995800
10456622	NM_013594 // Mbd1 // methyl-CpG binding domain protein 1 // 18 E2|18 45.0 cM //	Mbd1	NM_013594	0.0015882	0.0339771	1.8328600	1.8328600	0.0020306	0.4852520	1.1745400	1.1745400
10402073	NM_030172 // 2610021K21Rik // RIKEN cDNA 2610021K21 gene // 12 F1 // 78767 /// B	2610021K21Rik	NM_030172	0.0080414	0.0788949	1.8273100	1.8273100	0.0138261	0.5778020	1.3448300	1.3448300
10487453	NM_016902 // Nphp1 // nephronophthisis 1 (juvenile) homolog (human) // 2 F3 // 5	Nphp1	NM_016902	0.0101284	0.0889153	1.8132400	1.8132400	0.0425481	0.6296050	1.3760500	1.3760500
10396849	NM_009014 // Rad51l1 // RAD51-like 1 (S. cerevisiae) // 12 C3 // 19363 /// U9206	Rad51l1	NM_009014	0.0009331	0.0257794	1.8052200	1.8052200	0.0447184	0.6350840	1.1603900	1.1603900
10396956	NM_018814 // Pcnx // pecanex homolog (Drosophila) // 12 D1 // 54604 /// ENSMUST0	Pcnx	NM_018814	0.0003977	0.0182234	1.7993200	1.7993200	0.0088882	0.5362340	1.1266600	1.1266600
10527285	ENSMUST00000031618 // Rsph10b2 // radial spoke head 10 homolog B (Chlamydomonas)	Rsph10b2	ENSMUST00000031618	0.0018212	0.0364818	1.7862300	1.7862300	0.0027008	0.4958410	1.2626400	1.2626400
10338119	---		---	0.0444505	0.1981800	1.7709400	1.7709400	0.0417560	0.6296050	1.3160000	1.3160000
10343325	---		---	0.0080869	0.0791253	1.7408300	1.7408300	0.0272352	0.6015850	1.3848300	1.3848300
10417065	NM_029519 // Rap2a // RAS related protein 2a // 14 E4 // 76108 /// ENSMUST000000	Rap2a	NM_029519	0.0034505	0.0506831	1.7361300	1.7361300	0.0465490	0.6367960	1.1969000	1.1969000
10395816	NM_011968 // Psma6 // proteasome (prosome, macropain) subunit, alpha type 6 // 1	Psma6	NM_011968	0.0024645	0.0426825	1.7183800	1.7183800	0.0030411	0.5001020	1.0985100	1.0985100
10531133	NM_178700 // Grsf1 // G-rich RNA sequence binding factor 1 // 5 E1|5 48.0 cM //	Grsf1	NM_178700	0.0017183	0.0354179	1.7136500	1.7136500	0.0351091	0.6172700	1.1166600	1.1166600
10540952	NM_031177 // Ift122 // intraflagellar transport 122 homolog (Chlamydomonas) // 6	Ift122	NM_031177	0.0040263	0.0551381	1.7096400	1.7096400	0.0292603	0.6048710	1.3330300	1.3330300
10342346	---		---	0.0148480	0.1095320	1.7023700	1.7023700	0.0386750	0.6222000	1.2629800	1.2629800
10363983	NM_001081412 // Bcr // breakpoint cluster region // 10 B5.3-C1|10 40.5 cM // 110	Bcr	NM_001081412	0.0210762	0.1311730	1.7013300	1.7013300	0.0220726	0.5966630	1.1468400	1.1468400
10459804	NM_027721 // Katnal2 // katanin p60 subunit A-like 2 // 18 E3 // 71206 /// ENSMU	Katnal2	NM_027721	0.0246663	0.1428650	1.6854100	1.6854100	0.0238094	0.5966630	1.3557400	1.3557400
10438347	NM_011532 // Tbx1 // T-box 1 // 16 A3|16 11.35 cM // 21380 /// ENSMUST0000000924	Tbx1	NM_011532	0.0006017	0.0206123	1.6711500	1.6711500	0.0047467	0.5233400	1.2104100	1.2104100
10342324	---		---	0.0004639	0.0186104	1.6600000	1.6600000	0.0049988	0.5233400	1.1892600	1.1892600
10394978	NM_009104 // Rrm2 // ribonucleotide reductase M2 // 12 A1.3|12 7.0 cM // 20135 /	Rrm2	NM_009104	0.0071206	0.0741372	1.6590300	1.6590300	0.0391526	0.6222000	1.1979900	1.1979900
10386909	NM_028448 // Cenpv // centromere protein V // 11 B2 // 73139 /// BC147794 // Cen	Cenpv	NM_028448	0.0131648	0.1025610	1.6509700	1.6509700	0.0047341	0.5233400	1.3721900	1.3721900
10594110	NM_008684 // Neo1 // neogenin // 9 B // 18007 /// NM_001042752 // Neo1 // neogen	Neo1	NM_008684	0.0010403	0.0275286	1.6496400	1.6496400	0.0036246	0.5103990	1.4455900	1.4455900
10537353	NM_153600 // Ttc26 // tetratricopeptide repeat domain 26 // 6 B1 // 264134 /// E	Ttc26	NM_153600	0.0124975	0.0996998	1.6439300	1.6439300	0.0123030	0.5737140	1.2497500	1.2497500
10342735	---		---	0.0103195	0.0899090	1.6342500	1.6342500	0.0436148	0.6349300	1.3347400	1.3347400
10423363	NM_020332 // Ank // progressive ankylosis // 15 B1|15 14.4 cM // 11732 /// ENSMU	Ank	NM_020332	0.0008876	0.0251077	1.6214000	1.6214000	0.0455566	0.6350840	1.1376600	1.1376600
10352178	NM_178653 // Sccpdh // saccharopine dehydrogenase (putative) // 1 H4 // 109232 /	Sccpdh	NM_178653	0.0078274	0.0779724	1.6046300	1.6046300	0.0250269	0.5966630	1.2465000	1.2465000
10358579	NM_001024720 // Hmcn1 // hemicentin 1 // 1 G1 // 545370 /// ENSMUST00000074783 /	Hmcn1	NM_001024720	0.0044775	0.0579974	1.5972700	1.5972700	0.0221034	0.5966630	1.1167100	1.1167100
10496462	AK004863 // Adh6-ps1 // alcohol dehydrogenase 6 (class V), pseudogene 1 // 3 H1	Adh6-ps1	AK004863	0.0388962	0.1837300	1.5951000	1.5951000	0.0273896	0.6015850	1.4967100	1.4967100
10353549	BC070446 // Fam135a // family with sequence similarity 135, member A // 1 A5 //	Fam135a	BC070446	0.0058546	0.0663589	1.5933700	1.5933700	0.0026562	0.4958410	1.3374100	1.3374100
10581538	NM_008706 // Nqo1 // NAD(P)H dehydrogenase, quinone 1 // 8 D3|8 53.3 cM // 18104	Nqo1	NM_008706	0.0001216	0.0099346	1.5780800	1.5780800	0.0160854	0.5821440	1.3486000	1.3486000
10401428	ENSMUST00000110294 // C130039O16Rik // RIKEN cDNA C130039O16 gene // 12 D1 // 23	C130039O16Rik	ENSMUST00000110294	0.0002192	0.0134827	1.5700000	1.5700000	0.0172872	0.5851120	1.2123400	1.2123400
10352267	NM_001103182 // Lin9 // lin-9 homolog (C. elegans) // 1 H4 // 72568 /// NM_17518	Lin9	NM_001103182	0.0097938	0.0874103	1.5691100	1.5691100	0.0039057	0.5169010	1.2313400	1.2313400
10421293	NM_008915 // Ppp3cc // protein phosphatase 3, catalytic subunit, gamma isoform /	Ppp3cc	NM_008915	0.0058306	0.0662553	1.5645800	1.5645800	0.0093144	0.5362880	1.1199300	1.1199300
10469613	NM_177588 // Thnsl1 // threonine synthase-like 1 (bacterial) // 2 A3 // 208967 /	Thnsl1	NM_177588	0.0018910	0.0371887	1.5623800	1.5623800	0.0077613	0.5362340	1.5722900	1.5722900
10388520	NM_026029 // Glod4 // glyoxalase domain containing 4 // 11 B5 // 67201 /// ENSMU	Glod4	NM_026029	0.0147460	0.1091170	1.5618500	1.5618500	0.0379215	0.6190170	1.2597400	1.2597400
10413482	NM_009524 // Wnt5a // wingless-related MMTV integration site 5A // 14 A3|14 7.8	Wnt5a	NM_009524	0.0186135	0.1233130	1.5615600	1.5615600	0.0183658	0.5851120	1.7739900	1.7739900
10401181	NM_021557 // Rdh11 // retinol dehydrogenase 11 // 12 C3|12 32.5 cM // 17252 ///	Rdh11	NM_021557	0.0011192	0.0285265	1.5470800	1.5470800	0.0004236	0.3925740	1.5668400	1.5668400
10499954	NM_025420 // Lce1m // late cornified envelope 1M // --- // 66203 /// ENSMUST0000	Lce1m	NM_025420	0.0424622	0.1926090	1.5444400	1.5444400	0.0472956	0.6395460	1.9481800	1.9481800
10388185	NM_177776 // Smtnl2 // smoothelin-like 2 // 11 B4 // 276829 /// ENSMUST000001085	Smtnl2	NM_177776	0.0092475	0.0845670	1.5420600	1.5420600	0.0450456	0.6350840	1.2327000	1.2327000
10340607	---		---	0.0006026	0.0206211	1.5379500	1.5379500	0.0214457	0.5966630	1.1897600	1.1897600
10469522	NM_001001334 // BC061194 // cDNA sequence BC061194 // 2 A3 // 381350 /// BC04963	BC061194	NM_001001334	0.0072480	0.0747633	1.5318500	1.5318500	0.0497740	0.6441280	1.4633800	1.4633800
10451786	NM_172497 // Efhb // EF hand domain family, member B // 17 C // 211482 /// ENSMU	Efhb	NM_172497	0.0104446	0.0903572	1.5232200	1.5232200	0.0364738	0.6187790	1.8581400	1.8581400
10472128	NM_022989 // Arl6ip6 // ADP-ribosylation factor-like 6 interacting protein 6 //	Arl6ip6	NM_022989	0.0134188	0.1038570	1.5074300	1.5074300	0.0420888	0.6296050	1.1686800	1.1686800
10532275	NM_172429 // Smndc1 // survival motor neuron domain containing 1 // 19 D2 // 764	Smndc1	NM_172429	0.0174734	0.1193030	1.4928000	1.4928000	0.0007904	0.4532570	1.1191800	1.1191800
10418927	NM_009758 // Bmpr1a // bone morphogenetic protein receptor, type 1A // 14 B|14 1	Bmpr1a	NM_009758	0.0195530	0.1262900	1.4869100	1.4869100	0.0050078	0.5233400	1.1666200	1.1666200
10344391	---		---	0.0398298	0.1859620	1.4813600	1.4813600	0.0429329	0.6328870	1.4370000	1.4370000
10514919	ENSMUST00000069271 // Dmrtb1 // DMRT-like family B with proline-rich C-terminal,	Dmrtb1	ENSMUST00000069271	0.0178721	0.1205810	1.4720900	1.4720900	0.0021413	0.4852520	1.2388300	1.2388300
10372116	NM_027945 // Csl // citrate synthase like // 10 D1 // 71832 /// NM_027945 // Csl	Csl	NM_027945	0.0042708	0.0568364	1.4553400	1.4553400	0.0466585	0.6370520	1.6517400	1.6517400
10428070	AK020480 // 9430069I07Rik // RIKEN cDNA 9430069I07 gene // --- // 77358	9430069I07Rik	AK020480	0.0016616	0.0348547	1.4527700	1.4527700	0.0394936	0.6224180	1.3095600	1.3095600
10367691	NM_027391 // Iyd // iodotyrosine deiodinase // 10 A1 // 70337 /// ENSMUST0000001	Iyd	NM_027391	0.0472334	0.2045340	1.4472700	1.4472700	0.0465964	0.6368090	1.2666200	1.2666200
10339689	---		---	0.0452504	0.2000110	1.4408600	1.4408600	0.0345319	0.6172700	1.1485700	1.1485700
10341169	---		---	0.0207686	0.1301690	1.4379300	1.4379300	0.0116905	0.5687440	1.4553500	1.4553500
10408097	NM_019429 // Prss16 // protease, serine, 16 (thymus) // 13 A3-A5|13 10.0 cM // 5	Prss16	NM_019429	0.0409063	0.1885980	1.4353800	1.4353800	0.0102341	0.5509280	1.1005200	1.1005200
10400350	NM_007688 // Cfl2 // cofilin 2, muscle // 12 C1 // 12632 /// ENSMUST00000078124	Cfl2	NM_007688	0.0018056	0.0363741	1.4264900	1.4264900	0.0189849	0.5877510	1.1384500	1.1384500
10339373	---		---	0.0401266	0.1867000	1.4164900	1.4164900	0.0155207	0.5813830	1.3033900	1.3033900
10343967	---		---	0.0165455	0.1159430	1.4163700	1.4163700	0.0049487	0.5233400	1.3219500	1.3219500
10378065	NM_026068 // Med31 // mediator of RNA polymerase II transcription, subunit 31 ho	Med31	NM_026068	0.0064333	0.0699869	1.4098900	1.4098900	0.0332719	0.6144630	1.2739100	1.2739100
10399379	NM_008828 // Pgk1 // phosphoglycerate kinase 1 // X C-D|X 45.0 cM // 18655 /// E	Pgk1	NM_008828	0.0408068	0.1884440	1.4002400	1.4002400	0.0469002	0.6379430	1.0957700	1.0957700
10498313	NM_008828 // Pgk1 // phosphoglycerate kinase 1 // X C-D|X 45.0 cM // 18655 /// E	Pgk1	NM_008828	0.0408068	0.1884440	1.4002400	1.4002400	0.0469002	0.6379430	1.0957700	1.0957700
10461071	NM_175381 // 2700081O15Rik // RIKEN cDNA 2700081O15 gene // 19 A // 108899 /// E	2700081O15Rik	NM_175381	0.0211561	0.1314940	1.3934700	1.3934700	0.0194986	0.5890940	1.1893300	1.1893300
10547641	NM_011401 // Slc2a3 // solute carrier family 2 (facilitated glucose transporter)	Slc2a3	NM_011401	0.0140887	0.1065170	1.3904000	1.3904000	0.0002713	0.3777900	1.2408700	1.2408700
10344111	---		---	0.0113026	0.0943152	1.3851500	1.3851500	0.0247381	0.5966630	1.2034200	1.2034200
10608715	---		---	0.0018853	0.0371376	1.3750000	1.3750000	0.0444246	0.6350840	1.1274500	1.1274500
10398920	NM_145450 // BC022687 // cDNA sequence BC022687 // 12 F1|12 10.93 cM // 217887 /	BC022687	NM_145450	0.0270324	0.1501730	1.3704300	1.3704300	0.0105287	0.5561450	1.1781700	1.1781700
10394018	NM_027745 // Ccdc57 // coiled-coil domain containing 57 // 11 E2 // 71276 /// EN	Ccdc57	NM_027745	0.0005741	0.0202308	1.3667800	1.3667800	0.0076716	0.5362340	1.1870300	1.1870300
10353915	NM_027957 // Fam178b // family with sequence similarity 178, member B // 1 B //	Fam178b	NM_027957	0.0091378	0.0842235	1.3663300	1.3663300	0.0487294	0.6420950	1.2444200	1.2444200
10358615	NM_001024720 // Hmcn1 // hemicentin 1 // 1 G1 // 545370 /// ENSMUST00000074783 /	Hmcn1	NM_001024720	0.0219885	0.1343890	1.3653300	1.3653300	0.0190003	0.5877510	1.2837500	1.2837500
10421573	NM_007624 // Cbx3 // chromobox homolog 3 (Drosophila HP1 gamma) // 6 B-C|6 26.0	Cbx3	NM_007624	0.0065546	0.0707945	1.3635600	1.3635600	0.0348909	0.6172700	1.1812800	1.1812800
10354247	NM_010212 // Fhl2 // four and a half LIM domains 2 // 1 B // 14200 /// ENSMUST00	Fhl2	NM_010212	0.0116890	0.0959280	1.3616600	1.3616600	0.0155909	0.5813830	1.1188600	1.1188600
10519482	ENSMUST00000101627 // EG667705 // predicted gene, EG667705 // 5 A1 // 667705	EG667705	ENSMUST00000101627	0.0009668	0.0262007	1.3605200	1.3605200	0.0251060	0.5966630	1.2457000	1.2457000
10458999	NM_010181 // Fbn2 // fibrillin 2 // 18 D-E1|18 29.0 cM // 14119 /// ENSMUST00000	Fbn2	NM_010181	0.0494738	0.2094710	1.3601200	1.3601200	0.0335262	0.6144630	1.1870400	1.1870400
10499299	NM_022031 // Hapln2 // hyaluronan and proteoglycan link protein 2 // 3 F1|3 42.7	Hapln2	NM_022031	0.0389209	0.1838050	1.3590000	1.3590000	0.0343577	0.6172700	1.1026600	1.1026600
10563715	NM_153101 // Mrgpra2 // MAS-related GPR, member A2 // 7 B4 // 235712 /// NM_2075	Mrgpra2	NM_153101	0.0119338	0.0971650	1.3485200	1.3485200	0.0252392	0.5966630	1.0599700	1.0599700
10404284	NM_172156 // Prl3d3 // prolactin family 3, subfamily d, member 3 // 13 A3.1 // 2	Prl3d3	NM_172156	0.0100491	0.0887058	1.3461400	1.3461400	0.0254440	0.5966630	1.3525400	1.3525400
10458719	ENSMUST00000066328 // 9530002K18Rik // RIKEN cDNA 9530002K18 gene // 18 B3 // 77	9530002K18Rik	ENSMUST00000066328	0.0114195	0.0949101	1.3379200	1.3379200	0.0268800	0.6014670	1.2513700	1.2513700
10514128	NM_027238 // Ttc39b // tetratricopeptide repeat domain 39B // 4 C3 // 69863 ///	Ttc39b	NM_027238	0.0292176	0.1568250	1.3363400	1.3363400	0.0291570	0.6047360	1.1181100	1.1181100
10397216	NM_172582 // Coq6 // coenzyme Q6 homolog (yeast) // 12 D1 // 217707 /// ENSMUST0	Coq6	NM_172582	0.0037934	0.0532539	1.3324000	1.3324000	0.0089045	0.5362340	1.2766000	1.2766000
10501489	NM_144788 // Hectd1 // HECT domain containing 1 // 12 C1 // 207304 /// ENSMUST00	Hectd1	NM_144788	0.0027398	0.0450160	1.3297500	1.3297500	0.0474693	0.6397560	1.0937600	1.0937600
10464015	NM_019658 // Shoc2 // soc-2 (suppressor of clear) homolog (C. elegans) // 19 D2	Shoc2	NM_019658	0.0249772	0.1437310	1.3279100	1.3279100	0.0052543	0.5233400	1.1382300	1.1382300
10529445	NM_013587 // Lrpap1 // low density lipoprotein receptor-related protein associat	Lrpap1	NM_013587	0.0241764	0.1411470	1.3243100	1.3243100	0.0231485	0.5966630	1.1632100	1.1632100
10564573	NM_001081345 // Chd2 // chromodomain helicase DNA binding protein 2 // 7 D1 // 2	Chd2	NM_001081345	0.0144748	0.1078560	1.3207200	1.3207200	0.0001828	0.3629660	1.0779600	1.0779600
10523547	NM_172715 // Agpat9 // 1-acylglycerol-3-phosphate O-acyltransferase 9 // 5 E4 //	Agpat9	NM_172715	0.0112015	0.0937691	1.3161400	1.3161400	0.0318655	0.6124940	1.2943200	1.2943200
10395136	NM_001159743 // Fam150b // family with sequence similarity 150, member B // 12|1	Fam150b	NM_001159743	0.0476936	0.2054750	1.3134600	1.3134600	0.0381093	0.6199630	1.3195500	1.3195500
10363706	NM_207221 // Jmjd1c // jumonji domain containing 1C // 10 B5.1 // 108829 /// ENS	Jmjd1c	NM_207221	0.0308375	0.1616950	1.3112600	1.3112600	0.0013497	0.4796000	1.1066000	1.1066000
10591999	ENSMUST00000068033 // ENSMUSG00000054797 // predicted gene, ENSMUSG00000054797 /	ENSMUSG00000054797	ENSMUST00000068033	0.0108638	0.0923923	1.3062100	1.3062100	0.0358559	0.6185970	1.0723500	1.0723500
10472809	NM_010053 // Dlx1 // distal-less homeobox 1 // 2 C2|2 44.0 cM // 13390 /// ENSMU	Dlx1	NM_010053	0.0146082	0.1084810	1.3054600	1.3054600	0.0048668	0.5233400	1.4382700	1.4382700
10582196	BC027666 // 1190005I06Rik // RIKEN cDNA 1190005I06 gene // 8 E1 // 68918 /// ENS	1190005I06Rik	BC027666	0.0207495	0.1300950	1.2994700	1.2994700	0.0456450	0.6350840	1.2363700	1.2363700
10401398	---		---	0.0270530	0.1501730	1.2990300	1.2990300	0.0408604	0.6273030	1.2709700	1.2709700
10581575	NR_002928 // Gm1943 // gene model 1943, (NCBI) // 8 D3 // 384864 /// NM_0010814C	Gm1943	NR_002928	0.0338663	0.1700780	1.2955500	1.2955500	0.0146073	0.5813830	1.1065300	1.1065300
10344725	NM_175236 // Adhfe1 // alcohol dehydrogenase, iron containing, 1 // 1 A2 // 7618	Adhfe1	NM_175236	0.0087855	0.0825516	1.2940800	1.2940800	0.0179496	0.5851120	1.1940600	1.1940600
10348076	ENSMUST00000086964 // ENSMUSG00000067125 // predicted gene, ENSMUSG00000067125 /	ENSMUSG00000067125	ENSMUST00000086964	0.0338872	0.1701340	1.2938100	1.2938100	0.0374857	0.6190170	1.1303300	1.1303300
10447354	NM_025868 // Txndc14 // thioredoxin domain containing 14 // 2 D // 66958 /// BC1	Txndc14	NM_025868	0.0011974	0.0297754	1.2912100	1.2912100	0.0040051	0.5169010	1.0999500	1.0999500
10549265	NM_028742 // Ifltd1 // intermediate filament tail domain containing 1 // 6 G3|6	Ifltd1	NM_028742	0.0422301	0.1920720	1.2905500	1.2905500	0.0030943	0.5001020	1.3706600	1.3706600
10385310	NM_001099641 // Gabra6 // gamma-aminobutyric acid (GABA) A receptor, subunit alp	Gabra6	NM_001099641	0.0359849	0.1758010	1.2902000	1.2902000	0.0251089	0.5966630	1.2413700	1.2413700
10375713	NM_145926 // Mgat4b // mannoside acetylglucosaminyltransferase 4, isoenzyme B //	Mgat4b	NM_145926	0.0437565	0.1964650	1.2901700	1.2901700	0.0176804	0.5851120	1.3031800	1.3031800
10342958	---		---	0.0155723	0.1119240	1.2887000	1.2887000	0.0048856	0.5233400	1.2462400	1.2462400
10341069	---		---	0.0311612	0.1625060	1.2860200	1.2860200	0.0242021	0.5966630	1.2245100	1.2245100
10420889	ENSMUST00000050338 // 1110020C17Rik // RIKEN cDNA 1110020C17 gene // 14 D1 // 68	1110020C17Rik	ENSMUST00000050338	0.0170433	0.1179430	1.2846000	1.2846000	0.0470452	0.6382070	1.1611700	1.1611700
10358617	NM_001024720 // Hmcn1 // hemicentin 1 // 1 G1 // 545370 /// ENSMUST00000074783 /	Hmcn1	NM_001024720	0.0311055	0.1623110	1.2834100	1.2834100	0.0081030	0.5362340	1.1569500	1.1569500
10590149	NM_001033209 // Xylb // xylulokinase homolog (H. influenzae) // 9 F3 // 102448 /	Xylb	NM_001033209	0.0028865	0.0462717	1.2832800	1.2832800	0.0399703	0.6239170	1.0928400	1.0928400
10575151	ENSMUST00000046116 // C630050I24Rik // RIKEN cDNA C630050I24 gene // 8 D3 // 785	C630050I24Rik	ENSMUST00000046116	0.0147315	0.1090700	1.2812700	1.2812700	0.0034440	0.5103990	1.1604300	1.1604300
10346549	NM_007624 // Cbx3 // chromobox homolog 3 (Drosophila HP1 gamma) // 6 B-C|6 26.0	Cbx3	NM_007624	0.0287367	0.1554210	1.2790200	1.2790200	0.0111030	0.5628710	1.1573500	1.1573500
10566432	NM_147069 // Olfr686 // olfactory receptor 686 // --- // 259072 /// ENSMUST00000	Olfr686	NM_147069	0.0077707	0.0777201	1.2788800	1.2788800	0.0021788	0.4852520	1.0579100	1.0579100
10495332	BC116223 // 1700013F07Rik // RIKEN cDNA 1700013F07 gene // 3 F3 // 75504 /// BC1	1700013F07Rik	BC116223	0.0347156	0.1721780	1.2785600	1.2785600	0.0145689	0.5813830	1.2833100	1.2833100
10568792	NM_021302 // Stk32c // serine/threonine kinase 32C // 7 F4 // 57740 /// ENSMUST0	Stk32c	NM_021302	0.0493468	0.2091270	1.2777500	1.2777500	0.0279801	0.6015850	1.1190300	1.1190300
10419729	NM_001036293 // Nrbf2 // nuclear receptor binding factor 2 // 10 B5.1 // 641340	Nrbf2	NM_001036293	0.0001840	0.0122992	1.2776900	1.2776900	0.0371428	0.6187790	1.1603400	1.1603400
10419731	NM_001036293 // Nrbf2 // nuclear receptor binding factor 2 // 10 B5.1 // 641340	Nrbf2	NM_001036293	0.0001840	0.0122992	1.2776900	1.2776900	0.0371428	0.6187790	1.1603400	1.1603400
10542066	NM_026743 // Tspan11 // tetraspanin 11 // 6 F3 // 68498 /// ENSMUST00000032501 /	Tspan11	NM_026743	0.0427283	0.1933620	1.2775100	1.2775100	0.0403712	0.6254130	1.2379800	1.2379800
10447977	NM_175173 // Wdr27 // WD repeat domain 27 // 17 A2 // 71682 /// ENSMUST000000601	Wdr27	NM_175173	0.0057525	0.0656003	1.2773900	1.2773900	0.0279010	0.6015850	1.1385900	1.1385900
10390381	NM_008942 // Npepps // aminopeptidase puromycin sensitive // 11 D|11 56.0 cM //	Npepps	NM_008942	0.0096823	0.0867822	1.2756800	1.2756800	0.0015066	0.4796000	1.0621000	1.0621000

10606640	NM_172203 // Nox1 // NADPH oxidase 1 // X E3 // 237038 /// ENSMUST00000033610 //	Nox1	NM_172203	0.0441651	0.1974710	1.2752400	1.2752400	0.0456763	0.6350840	1.3105600	1.3105600
10483626	NM_010054 // Dlx2 // distal-less homeobox 2 // 2 C2|2 44.0 cM // 13392 /// ENSMU	Dlx2	NM_010054	0.0243255	0.1415810	1.2740300	1.2740300	0.0309866	0.6089260	1.2621900	1.2621900
10521068	NM_001081101 // 4933407H18Rik // RIKEN cDNA 4933407H18 gene // 5 B1 // 71101 ///	4933407H18Rik	NM_001081101	0.0377119	0.1804450	1.2712400	1.2712400	0.0163674	0.5851120	1.2541300	1.2541300
10460782	NM_130453 // Gpha2 // glycoprotein hormone alpha 2 // 19 A // 170458 /// ENSMUST	Gpha2	NM_130453	0.0397053	0.1855630	1.2695900	1.2695900	0.0003728	0.3867110	1.3764400	1.3764400
10338448	---		---	0.0452720	0.2000110	1.2695400	1.2695400	0.0249379	0.5966630	1.2909000	1.2909000
10445839	NM_177052 // Kif6 // kinesin family member 6 // 17 C // 319991 /// ENSMUST000000	Kif6	NM_177052	0.0154692	0.1114920	1.2686800	1.2686800	0.0244108	0.5966630	1.2277000	1.2277000
10445678	NM_145489 // AI661453 // expressed sequence AI661453 // 17 C // 224833 /// BC006	AI661453	NM_145489	0.0173491	0.1188930	1.2674400	1.2674400	0.0185295	0.5851120	1.1041300	1.1041300
10573008	NM_178267 // Zfp827 // zinc finger protein 827 // 8 C1-C2 // 622675 /// ENSMUST0	Zfp827	NM_178267	0.0396537	0.1854030	1.2674200	1.2674200	0.0194761	0.5890940	1.2349300	1.2349300
10361846	NM_009048 // Reps1 // RalBP1 associated Eps domain containing protein // 10 A3 /	Reps1	NM_009048	0.0096342	0.0865497	1.2670800	1.2670800	0.0226815	0.5966630	1.1247800	1.1247800
10358605	NM_001024720 // Hmcn1 // hemicentin 1 // 1 G1 // 545370 /// ENSMUST00000074783 /	Hmcn1	NM_001024720	0.0472177	0.2045160	1.2652400	1.2652400	0.0489029	0.6420950	1.1660800	1.1660800
10359136	NM_001033180 // 9430070O13Rik // RIKEN cDNA 9430070O13 gene // 1 G3 // 77352 ///	9430070O13Rik	NM_001033180	0.0175703	0.1196340	1.2642200	1.2642200	0.0180074	0.5851120	1.2857200	1.2857200
10498323	NM_001081262 // 4932431H17Rik // RIKEN cDNA 4932431H17 gene // 3 D // 545527 ///	4932431H17Rik	NM_001081262	0.0007842	0.0235685	1.2637500	1.2637500	0.0049377	0.5233400	1.1621200	1.1621200
10472598	NM_177784 // Klhl23 // kelch-like 23 (Drosophila) // 2 C2 // 277396 /// ENSMUST0	Klhl23	NM_177784	0.0141307	0.1066480	1.2589700	1.2589700	0.0009181	0.4730920	1.6846300	1.6846300
10397575	NM_172544 // Nrxn3 // neurexin III // 12D2|12 45.0 cM // 18191 /// ENSMUST000000	Nrxn3	NM_172544	0.0476188	0.2053030	1.2517300	1.2517300	0.0033632	0.5103990	1.2675800	1.2675800
10594825	NM_022026 // Aqp9 // aquaporin 9 // 9 D // 64008 /// ENSMUST00000074465 // Aqp9	Aqp9	NM_022026	0.0219558	0.1343070	1.2494800	1.2494800	0.0325958	0.6144630	1.1198400	1.1198400
10507580	NM_133892 // Lao1 // L-amino acid oxidase 1 // 4 D2.1 // 100470 /// ENSMUST00000	Lao1	NM_133892	0.0338348	0.1700150	1.2469100	1.2469100	0.0370077	0.6187790	1.1848700	1.1848700
10493690	ENSMUST00000029548 // Nup210l // nucleoporin 210-like // 3 F2 // 77595	Nup210l	ENSMUST00000029548	0.0074844	0.0761196	1.2458200	1.2458200	0.0090017	0.5362340	1.3992000	1.3992000
10521136	NM_001081102 // Whsc1 // Wolf-Hirschhorn syndrome candidate 1 (human) // 5 B1 //	Whsc1	NM_001081102	0.0056046	0.0644497	1.2450700	1.2450700	0.0088559	0.5362340	1.2025000	1.2025000
10396278	NM_026102 // Daam1 // dishevelled associated activator of morphogenesis 1 // 12	Daam1	NM_026102	0.0046071	0.0587162	1.2405200	1.2405200	0.0494036	0.6430890	1.1631300	1.1631300
10570585	---		---	0.0055020	0.0638978	1.2402000	1.2402000	0.0030137	0.5001020	1.1851500	1.1851500
10502375	NM_008642 // Mttp // microsomal triglyceride transfer protein // 3 G3|3 66.2 cM	Mttp	NM_008642	0.0117946	0.0964307	1.2367000	1.2367000	0.0368218	0.6187790	1.1612800	1.1612800
10550022	NM_133948 // Psip1 // PC4 and SFRS1 interacting protein 1 // 4 C3 // 101739 ///	Psip1	NM_133948	0.0005901	0.0205357	1.2231400	1.2231400	0.0035854	0.5103990	1.1521900	1.1521900
10564169	AF241256 // Snord116 // small nucleolar RNA, C/D box 116 cluster // 7 C|7 29.0 c	Snord116	AF241256	0.0230070	0.1374940	1.2230500	1.2230500	0.0285367	0.6032410	1.2990600	1.2990600
10446986	NM_015800 // Crim1 // cysteine rich transmembrane BMP regulator 1 (chordin like)	Crim1	NM_015800	0.0492016	0.2088600	1.2225600	1.2225600	0.0032959	0.5103990	1.1685400	1.1685400
10496077	NM_027907 // Agxt2l1 // alanine-glyoxylate aminotransferase 2-like 1 // 3 G3 //	Agxt2l1	NM_027907	0.0363949	0.1767900	1.2213800	1.2213800	0.0016389	0.4825080	1.0607900	1.0607900
10361007	NM_026796 // Smyd2 // SET and MYND domain containing 2 // 1 H6 // 226830 /// ENS	Smyd2	NM_026796	0.0408162	0.1884440	1.2212900	1.2212900	0.0342217	0.6172700	1.2211900	1.2211900
10457382	NM_153086 // Gjd4 // gap junction protein, delta 4 // 18 A1 // 225152 /// ENSMUS	Gjd4	NM_153086	0.0336691	0.1694830	1.2200100	1.2200100	0.0494127	0.6430890	1.0987900	1.0987900
10461856	NM_008137 // Gna14 // guanine nucleotide binding protein, alpha 14 // 19 A-B|19	Gna14	NM_008137	0.0168781	0.1172790	1.2168800	1.2168800	0.0155827	0.5813830	1.2639400	1.2639400
10474207	NM_212433 // Fbxo3 // F-box protein 3 // 2 E2 // 57443 /// NM_020593 // Fbxo3 //	Fbxo3	NM_212433	0.0260184	0.1468810	1.2109700	1.2109700	0.0045122	0.5233400	1.1569100	1.1569100
10359446	BC138379 // AI848100 // expressed sequence AI848100 // 1 H2.1 // 226551 /// ENSM	AI848100	BC138379	0.0451227	0.1998720	1.2061900	1.2061900	0.0053660	0.5233400	1.1639000	1.1639000
10427661	---		---	0.0013679	0.0316233	1.2053400	1.2053400	0.0085097	0.5362340	1.2110300	1.2110300
10410148	NM_001081129 // Cntnap3 // contactin associated protein-like 3 // 13 B3 // 23868	Cntnap3	NM_001081129	0.0084408	0.0806418	1.2031700	1.2031700	0.0347180	0.6172700	1.1258800	1.1258800
10521383	NM_007418 // Adra2c // adrenergic receptor, alpha 2c // 5 B2|5 20.0 cM // 11553	Adra2c	NM_007418	0.0395531	0.1852160	1.2012700	1.2012700	0.0217295	0.5966630	1.0901900	1.0901900
10359422	NM_007453 // Prdx6 // peroxiredoxin 6 // 1 H2.1|1 83.6 cM // 11758 /// ENSMUST00	Prdx6	NM_007453	0.0437698	0.1964750	1.1996900	1.1996900	0.0291687	0.6047360	1.1236600	1.1236600
10540507	NM_008210 // H3f3a // H3 histone, family 3A // 1 D2.3 // 15078 /// BC083353 // H	H3f3a	NM_008210	0.0470108	0.2039680	1.1993500	1.1993500	0.0173517	0.5851120	1.0691600	1.0691600
10386741	ENSMUST00000074908 // 3110043A19Rik // RIKEN cDNA 3110043A19 gene // 11 B2 // 73	3110043A19Rik	ENSMUST00000074908	0.0403655	0.1873190	1.1959700	1.1959700	0.0063170	0.5233400	1.1407800	1.1407800
10438189	NM_144852 // Slc7a4 // solute carrier family 7 (cationic amino acid transporter,	Slc7a4	NM_144852	0.0348129	0.1723970	1.1892900	1.1892900	0.0218423	0.5966630	1.2164200	1.2164200
10517058	NM_001083916 // 1810019J16Rik // RIKEN cDNA 1810019J16 gene // 4 D2.3 // 69073 /	1810019J16Rik	NM_001083916	0.0301141	0.1595320	1.1885500	1.1885500	0.0402122	0.6253770	1.1297100	1.1297100
10591210	NM_146558 // Olfr866 // olfactory receptor 866 // --- // 258551 /// ENSMUST00000	Olfr866	NM_146558	0.0029165	0.0465222	1.1882500	1.1882500	0.0035488	0.5103990	1.1046100	1.1046100
10554140	BC116191 // Zic4 // zinc finger protein of the cerebellum 4 // 9 E3.3|9 61.0 cM	Zic4	BC116191	0.0124328	0.0993844	1.1859600	1.1859600	0.0082483	0.5362340	1.1053000	1.1053000
10373660	NM_146777 // Olfr818 // olfactory receptor 818 // --- // 258773 /// ENSMUST00000	Olfr818	NM_146777	0.0261747	0.1472200	1.1842600	1.1842600	0.0158094	0.5813830	1.1566000	1.1566000
10538571	NM_053231 // V1rc1 // vomeronasal 1 receptor, C1 // 6 B3 // 113858 /// NM_134185	V1rc1	NM_053231	0.0053865	0.0632626	1.1810600	1.1810600	0.0355343	0.6172700	1.1235100	1.1235100
10358631	NM_001024720 // Hmcn1 // hemicentin 1 // 1 G1 // 545370 /// ENSMUST00000074783 /	Hmcn1	NM_001024720	0.0151771	0.1105820	1.1810200	1.1810200	0.0448210	0.6350840	1.0358900	1.0358900
10437737	NM_025546 // Rsl1d1 // ribosomal L1 domain containing 1 // 16 B1 // 66409 /// EN	Rsl1d1	NM_025546	0.0327645	0.1672130	1.1810200	1.1810200	0.0448754	0.6350840	1.0673500	1.0673500
10534253	NM_001081462 // Gtf2ird1 // general transcription factor II I repeat domain-cont	Gtf2ird1	NM_001081462	0.0441150	0.1973850	1.1801600	1.1801600	0.0122261	0.5737140	1.3599800	1.3599800
10408367	NM_008232 // Hdgfl1 // hepatoma derived growth factor-like 1 // 13 A3.1 // 15192	Hdgfl1	NM_008232	0.0201378	0.1282040	1.1775200	1.1775200	0.0018878	0.4852520	1.1927500	1.1927500
10398336	---		---	0.0175408	0.1194790	1.1753200	1.1753200	0.0413185	0.6296050	1.1580200	1.1580200
10477495	NM_001012392 // U46068 // cDNA sequence U46068 // 2 H1 // 228801 /// NM_153418 /	U46068	NM_001012392	0.0434138	0.1954130	1.1746600	1.1746600	0.0000120	0.1067480	1.1877900	1.1877900
10564159	AF241256 // Snord116 // small nucleolar RNA, C/D box 116 cluster // 7 C|7 29.0 c	Snord116	AF241256	0.0279570	0.1530940	1.1733300	1.1733300	0.0378411	0.6190170	1.2204400	1.2204400
10503374	---		---	0.0465304	0.2027730	1.1709600	1.1709600	0.0482236	0.6417260	1.1164400	1.1164400
10601545	---		---	0.0434569	0.1955360	1.1681900	1.1681900	0.0046514	0.5233400	1.1807100	1.1807100
10560270	NM_008718 // Npas1 // neuronal PAS domain protein 1 // 7 A2|7 4.0 cM // 18142 //	Npas1	NM_008718	0.0413704	0.1896550	1.1672100	1.1672100	0.0339938	0.6166390	1.1665800	1.1665800
10460631	NM_009045 // Rela // v-rel reticuloendotheliosis viral oncogene homolog A (avian	Rela	NM_009045	0.0317343	0.1641900	1.1651500	1.1651500	0.0377845	0.6190170	1.1435700	1.1435700
10473270	---		---	0.0327779	0.1672580	1.1606900	1.1606900	0.0459086	0.6351460	1.2043900	1.2043900
10584386	NM_146806 // Olfr143 // olfactory receptor 143 // --- // 258802 /// ENSMUST00000	Olfr143	NM_146806	0.0122221	0.0984748	1.1594200	1.1594200	0.0243690	0.5966630	1.0959700	1.0959700
10471298	NM_001159634 // Bat2l // HLA-B associated transcript 2-like // 2 B // 227723 ///	Bat2l	NM_001159634	0.0064942	0.0703273	1.1580900	1.1580900	0.0252456	0.5966630	1.1493200	1.1493200
10510957	NM_172990 // Pank4 // pantothenate kinase 4 // 4 E2 // 269614 /// ENSMUST0000003	Pank4	NM_172990	0.0120579	0.0977279	1.1467800	1.1467800	0.0304296	0.6077970	1.1789900	1.1789900
10461713	NM_146684 // Olfr1440 // olfactory receptor 1440 // --- // 258679 /// ENSMUST000	Olfr1440	NM_146684	0.0489931	0.2084230	1.1452900	1.1452900	0.0229882	0.5966630	1.1837000	1.1837000
10461818	NM_146636 // Olfr1487 // olfactory receptor 1487 // --- // 258629 /// ENSMUST000	Olfr1487	NM_146636	0.0398115	0.1859130	1.1450900	1.1450900	0.0303615	0.6077970	1.1653900	1.1653900
10368997	AK048022 // C130030K03Rik // RIKEN cDNA C130030K03 gene // --- // 320644	C130030K03Rik	AK048022	0.0345038	0.1717750	1.1406200	1.1406200	0.0039365	0.5169010	1.1765800	1.1765800
10521088	ENSMUST00000101353 // ENSMUSG00000073044 // predicted gene, ENSMUSG00000073044 /	ENSMUSG00000073044	ENSMUST00000101353	0.0056937	0.0651580	1.1352100	1.1352100	0.0494772	0.6431660	1.1585100	1.1585100
10416110	NM_001034881 // EG432870 // predicted gene, EG432870 // 14 D1|14 // 432870 /// E	EG432870	NM_001034881	0.0232150	0.1381480	1.1268700	1.1268700	0.0075418	0.5357610	1.1496700	1.1496700
10492625	---		---	0.0370319	0.1786330	1.1022200	1.1022200	0.0021480	0.4852520	1.1875700	1.1875700
10338558	---		---	0.0149110	0.1098350	1.0998700	1.0998700	0.0055793	0.5233400	1.1096800	1.1096800
10489422	NM_008435 // Kcns1 // K+ voltage-gated channel, subfamily S, 1 // 2 H3 // 16538	Kcns1	NM_008435	0.0228289	0.1369170	1.0599500	1.0599500	0.0048196	0.5233400	1.2466500	1.2466500

Supplementary Table 4: Sequences of oligonucleotide primers used for qRT-PCR

Gene	Forward Sequence	Reverse Sequence
Pthrp	5' TTC GGT GGA GGG GCT TGG CC	5' CGG CGG CGC AAG TCT TGG AT
S100a8	5' CAC CAT GCC CTC TAC AAG AAT G	5' TCA CCA TCG CAA GGA ACT CC
S100a9	5' GGA AGC ACA GTT GGC AAC	5' TCC AGG TCC TCC ATG ATG TCA
Egfr	5' CTG TCG CAA AGT TTG TAA TG	5' GAA TTT CTA GTT CTC GTG GG
MMP-9	5' CCA GAC ACT AAA GGC CAT TCG A	5' TCC CAC TTG AGG CCT TTG AAG
Rps27a	5' GAC CCT TAC GGG GAA AAC CAT	5' AGA CAA AGT CCG GCC ATC TTC
